# Blood pressure during long-term cilostazol-based dual antiplatelet therapy after stroke: a post hoc analysis of the CSPS.com trial

**DOI:** 10.1038/s41440-024-01742-3

**Published:** 2024-07-09

**Authors:** Kazunori Toyoda, Masatoshi Koga, Kenta Tanaka, Shinichiro Uchiyama, Hisato Sunami, Katsuhiro Omae, Kazumi Kimura, Haruhiko Hoshino, Mayumi Fukuda-Doi, Kaori Miwa, Junpei Koge, Yasushi Okada, Nobuyuki Sakai, Kazuo Minematsu, Takenori Yamaguchi, for the CSPS.com Trial Investigators, Takenori Yamaguchi, Takenori Yamaguchi, Kazunori Toyoda, Shinichiro Uchiyama, Haruhiko Hoshino, Kazumi Kimura, Yasushi Okada, Nobuyuki Sakai, Kotaro Tanaka, Kazunori Toyoda, Hiroaki Naritomi, Shinya Goto, Tatsuya Isomura, Kazuo Minematsu, Kiyohiro Houkin, Masayasu Matsumoto, Yasuo Terayama, Hidekazu Tomimoto, Teiji Tominaga, Satoshi Yasuda, Hideki Orikasa, Naoko Kumagai, Akihiro Miyasaki, Masanori Isobe, Yoshitaka Suda, Kazuo Kitagawa, Kazuyuki Nagatsuka, Shuta Toru, Makoto Katsuno, Kenichi Murao, Norio Ikeda, Kazuya Nakashima, Shinichi Okabe, Masanori Kurimoto, Ikuo Ihara, Hideki Matsuoka, Shoji Mabuchi, Hideo Hara, Teruyuki Yoshimoto, Takeshi Matsuoka, Yoshikazu Arai, Yasuyuki Iwasaki, Manabu Hattori, Kazuya Takahashi, Yoshihisa Fukushima, Masayuki Ezura, Yasuaki Takeda, Kimihiro Nakahara, Masahiro Okada, Shingo Mitaki, Kosuke Yoshida, Kenji Kamiyama, Takahiro Kuwashiro, Takeshi Iwanaga, Akira Takahashi, Junichi Maruyama, Teiji Yamamoto, Michiyuki Maruyama, Yoshiharu Taguchi, Kazuhiro Hashidume, Katsumi Takizawa, Yasuyuki Iguchi, Kazuhito Kitajima, Shinichi Yoshimura, Syuji Arakawa, Takeshi Inoue, Hiroyuki Yamaguchi, Susumu Suzuki, Youichi Watanabe, Daisuke Yasutomi, Ryota Tanaka, Takuji Yamamoto, Tetsuo Ando, Yasuhiro Ito, Naoki Hattori, Nobutaka Yamamoto, Tsutomu Takahashi, Syoji Arihiro, Naoaki Kanda, Hirotoshi Hamaguchi, Junji Kasuya, Masaru Honda, Hiroshi Oyama, Hidefumi Yoshida, Satoshi Okuda, Keita Matsuura, Toshiaki Ieda, Takao Kanzawa, Makio Takahashi, Hirokazu Sadahiro, Takahiro Miyahara, Masahiko Yamada, Takeshi Aoki, Taizen Nakase, Katsuhiko Hayashi, Toshitaka Umemura, Yasukuni Tsugu, Fumitaka Miya, Ryo Otani, Keiichi Yamada, Yoshinaga Kajimoto, Hiroshi Nakane, Kiyohito Shinno, Akio Hara, Ryoichi Saito, Yuzo Araki, Toshiho Otsuki, Koji Abe, Shigenari Kin, Takehisa Tsuji, Shota Sakai, Yoshio Tsuboi, Atsushi Kawamorita, Hiroaki Shimizu, Nobuo Araki, Takashi Hata, Hiroshi Ryu, Kazumasa Yamatani, Shinji Minami, Takahiro Maruta, Masaki Eto, Katsutoshi Takayama, Kazuo Hashikawa, Eiichiro Mabuchi, Yoshio Sakagami, Syoji Tsuchimoto, Jiro Kitayama, Kiyoshi Shirakawa, Haruki Takahashi, Syunro Uchinokura, Naohiro Osaka, Ichiro Imafuku, Toshiro Otsuka, Ryo Itabashi, Yuji Kujiraoka, Naohisa Miura, Koichi Nomura, Masahiro Kobari, Keizo Yasui, Susumu Kashino, Hiroto Murata, Kazuhiko Nozaki, Kosuke Yamashita, Katsumi Matsumoto, Yuji Shibata, Atsuo Aoyama, Yoshimasa Watanabe, Toru Eto, Susumu Mekaru, Tsuneo Honda, Masato Seike, Masahiro Kurisaka, Toshio Imaizumi, Kojiro Wada, Norihiro Suzuki, Atsuo Yoshino, Yukiko Hara, Shunya Takizawa, Kaoru Kamimoto, Hiroshi Iizuka, Yasuo Toma, Taro Komuro, Atsushi Sueyoshi, Yoshikazu Nakajima, Takayuki Sakaki, Hiroji Miyake, Masaru Idei, Tsutomu Hitotsumatsu, Shigehiro Nakahara, Masahiko Kawanishi, Takuji Kitaoka, Naoyuki Isobe, Masanobu Hokama, Toshihide Shibata, Kazuhito Tsuruta, Akihito Moriki, Masahiro Makino, Masafumi Otaki, Minoru Ajiki, Takaaki Yamazaki, Kiyohiro Houkin, Nobuyuki Yasui, Koichi Hirata, Hiroyuki Kato, Ichiro Suzuki, Takakazu Kawamata, Yoshikazu Uesaka, Kohei Yamashita, Yukiko Enomoto, Osamu Onodera, Masato Ikeda, Susumu Miyamoto, Manabu Sakaguchi, Hiroyuki Nakase, Yoshiki Yagita, Tetsuhiro Kitahara, Katsumi Irie, Tomohiko Kusuhara, Kazumasa Kawazoe, Shinji Nagahiro, Norikazu Kawada, Akiko Adachi, Toshihiro Fukusako, Wataro Tsuruta, Naoko Fujimura, Takayuki Koizumi, Hiroyuki Tomimitsu, Shigeru Fujimoto, Tsukasa Tsuchiya, Hitoshi Aizawa, Nobutaka Ishizu, Shigeru Nogawa, Hideharu Furumoto, Toshihiro Ueda, Syogo Imae, Teiji Nakayama, Hiroki Namba, Jun Ochiai, Tomoko Yamana, Mitsuhito Mase, Noriyuki Matsukawa, Hisayoshi Niwa, Masatoshi Muramatsu, Yoshio Nakashima, Fuminori Iwamoto, Syunichi Yoneda, Kenji Hashimoto, Tatsuo Matsushita, Takenobu Kunieda, Osamu Masuo, Hirotaka Yamamoto, Naohisa Hosomi, Ryo Ogami, Koichi Kuramoto, Takahiro Matsumoto, Hirotsugu Shinozaki, Hiroshi Sugimori, Yoichiro Hashimoto, Hidenori Suzuki, Masahiro Waza, Yuki Kujyuro, Eiichi Kamei, Yasufumi Uchida, Masao Nagayama, Masahiko Hiroki, Hiroshi Sakura, Tatsuru Noujo, Yasutaka Tajima, Hajime Wada, Akira Hodozuka, Wataru Ide, Yasushi Shibata, Shinji Yamamoto, Masayuki Ishihara, Satsuki Miyata, Yoshiyuki Matsuoka, Yasuhisa Sakurai, Yoshiharu Miura, Takanori Yokota, Satoshi Iwabuchi, Wataro Tsuruta, Hirohiko Arimoto, Sumio Suda, Takashi Ohashi, Katsuhiro Kuroda, Takashi Matsuhisa, Kazutoshi Yokoyama, Eiichi Katada, Kenichiro Fujishiro, Akira Inukai, Yasushi Kobayashi, Hideki Sakai, Kenichi Yamamoto, Ken Asakura, Yuhei Yoshimoto, Yoshikazu Kusano, Ryoichi Takahashi, Sotaro Higashi, Cheho Park, Mitsutoshi Nakada, Makoto Matsui, Yoshinari Nagakane, Akira Yoshioka, Masahiro Makino, Kazuyoshi Yamaguchi, Yasushi Hagihara, Tomonori Yamada, Kenji Hashimoto, Toshiaki Fujita, Tetsuya Kumagai, Masayuki Sumida, Motohiro Morioka, Hiroaki Oboshi, Takanari Kitazono, Yukio Ando, Seiichiro Minato, Masahito Agawa, Takeshi Kono, Tomohiko Izumidani, Tetsuya Ueba, Hiroaki Takeuchi, Syuji Monden, Syoji Shiraishi, Hidehiko Syoji, Tatsuya Nakamura, Naoki Ikawa, Hiroshi Sugihara, Shinichi Toyonaga, Hiroyuki Kon, Yuji Kanamori, Hiroaki Tanaka

**Affiliations:** 1 Department of Cerebrovascular Medicine, National Cerebral and Cardiovascular Center, Suita, Japan; 2 Department of Data Science, National Cerebral and Cardiovascular Center, Suita, Japan; 3 Clinical Research Center for Medicine, International University of Health and Welfare, Center for Brain and Cerebral Vessels, Sanno Medical Center, Tokyo, Japan; 4 Department of Biostatistics, National Cerebral and Cardiovascular Center, Suita, Japan; 5 Department of Neurological Science, Graduate School of Medicine, Nippon Medical School, Tokyo, Japan; 6 Department of Neurology, Tokyo Saiseikai Central Hospital, Tokyo, Japan; 7 Clinical Research Institute and Department of Cerebrovascular Medicine and Neurology, National Hospital Organization Kyushu Medical Center, Fukuoka, Japan; 8 Department of Neurosurgery, Kobe City Medical Center General Hospital, Kobe, Japan; 9 Headquarters of the Iseikai Medical Corporation, Osaka, Japan; 10 Japan Cardiovascular Research Foundation, Suita, Japan; 11 National Cerebral and Cardiovascular Center, Suita, Japan; 12 Sanno Hospital and Sanno Medical Center, Suita, Japan; 13 Tokyo Saiseikai Central Hospital, Tokyo, Japan; 14 Nippon Medical School, Tokyo, Japan; 15 Kyushu Medical Center, Fukuoka, Japan; 16 Kobe City Medical Center General Hospital, Kobe, Japan; 17 University of Toyama, Toyama, Japan; 18 Senri Chuo Hospital, Toyonaka, Japan; 19 Tokai University School of Medicine, Isehara, Japan; 20 Clinical Study Support, Inc., Nagoya, Japan; 21 Medical Corporation Iseikai, Osaka, Japan; 22 Hokkaido University, Sapporo, Japan; 23 Sakai City Medical Center, Sakai, Japan; 24 Iwate Medical University, Morioka, Japan; 25 Mie University, Tsushima, Japan; 26 Tohoku University, Sendai, Japan; 27 Ichinomiya Nishi Hospital, Ichinomiya, Japan; 28 Kushiro Rosai Hospital, Kushiro, Japan; 29 Yuri Kumiai General Hospital, Yuri-Honjo, Japan; 30 Tokyo Women’s Medical University Hospital, Tokyo, Japan; 31 Nitobe Memorial Nakano General Hospital, Tokyo, Japan; 32 Doutou neurosurgery, Kitami, Japan; 33 Shiroyama Hospital, Habikino, Japan; 34 Ube Kohsan Central Hospital, Ube, Japan; 35 Kobe Ekisaikai Hospital, Kobe, Japan; 36 Seirei Memorial Hospital, Hitachi, Japan; 37 Kurobe City Hospital, Kurobe, Japan; 38 Sakurakai Hospital, Osakasayama, Japan; 39 NHO Kagoshima Medical Center, Kagoshima, Japan; 40 Otaru General Hospital, Otaru, Japan; 41 Saga University Hospital, Saga, Japan; 42 Kashiwaba Neurosurgical Hospital, Sapporo, Japan; 43 Date Red Cross Hospital, Date, Japan; 44 Municipal Tsuruga Hospital, Tsuruga, Japan; 45 Toho University Omori Medical Center, Tokyo, Japan; 46 Daido Hospital, Nagoya, Japan; 47 Japanese Red Cross Society Himeji Hospital, Himeji, Japan; 48 St.Mary’s Hospital, Kurume, Japan; 49 Sendai Medical Center, Sendai, Japan; 50 JCHO Tokyo Yamate Medical Center, Tokyo, Japan; 51 Takagi Hospital, Oume, Japan; 52 Sumoto Itsuki Hospital, Sumoto, Japan; 53 Shimane University Hospital, Izumo, Japan; 54 NHO Asahikawa Meidical Center, Asahikawa, Japan; 55 Nakamura Memorial Hospital, Sapporo, Japan; 56 NHO Kyushu Medical Center, Fukuoka, Japan; 57 Japan Red Cross Okayama Hospital, Okayama, Japan; 58 Sapporo Shiroishi Memorial Hospital, Sapporo, Japan; 59 Asahikawa Rehabilitation Hospital, Asahikawa, Japan; 60 Southern TOHOKU General Hospital, Koriyama, Japan; 61 Saiseikai Yokohamashi Tobu Hospital, Yokohama, Japan; 62 Toyama University Hospital, Toyama, Japan; 63 Fujieda Heisei Memorial Hospital, Fujieda, Japan; 64 Japanese Red Cross Asahikawa Hospital, Asahikawa, Japan; 65 Jikei University, Tokyo, Japan; 66 Yokohamashintoshi Neurosurgical Hospital, Yokohama, Japan; 67 Hyogo College of Medicine Hospital, Nishinomiya, Japan; 68 Steel Memorial Yawata Hospital, Kitakyushu, Japan; 69 Kawasaki Hospital, Kurashiki, Japan; 70 Ohkawara Neurosurgical Hospital, Muroran, Japan; 71 Rumoi Central Clinic, Rumoi, Japan; 72 Japanese Red Cross Fukushima Hospital, Fukushima, Japan; 73 Tokyo Medical Center, Tokyo, Japan; 74 Juntendo University Hospital, Tokyo, Japan; 75 Juntendo University Shizuoka Hospital, Shizuoka, Japan; 76 Anjo Kosei Hospital, Anjo, Japan; 77 TOYOTA Memorial Hospital, Toyota, Japan; 78 Toyota Kosei Hospital, Toyota, Japan; 79 Kanazawa Neurosurgical Hospital, Kanazawa, Japan; 80 JCHO Hoshigaoka Medical Center, Hirakata, Japan; 81 JOHAS Kyushu Rosai Hospital, Kitakyushu, Japan; 82 Imamura General Hospital, Kagoshima, Japan; 83 Kita-Harima Medical Center, Ono, Japan; 84 Atsuchi Neurosurgical Hospital, Kagoshima, Japan; 85 Shunan Memorial Hospital, Kudamatsu, Japan; 86 Muroran City General Hospital, Muroran, Japan; 87 Shizuoka General Hospital, Shizuoka, Japan; 88 Nagoya Medical Center, Nagoya, Japan; 89 Suzuka Kaisei Hospital, Suzuka, Japan; 90 Yokkaichi Municipal Hospital, Yokkaichi, Japan; 91 Mihara Memorial Hospital, Isezaki, Japan; 92 Japanese Red Cross Osaka Hospital, Osaka, Japan; 93 Yamaguchi University Hospital, Ube, Japan; 94 Yame General Hospital, Yame, Japan; 95 Kagoshima City Hospital, Kagoshima, Japan; 96 Hokkaido Neurosurgical Memorial Hospital, Sapporo, Japan; 97 Research Institute for Brain and Blood Vessels-Akita, Akita, Japan; 98 Ogaki Tokushukai Hospital, Ogaki, Japan; 99 JOHAS Chubu Rosai Hospital, Nagoya, Japan; 100 Toyokawa City Hospital, Toyokawa, Japan; 101 Japanese Red Cross Ise Hospital, Ise, Japan; 102 NHO Kyoto Medical Center, Kyoto, Japan; 103 Kansai Electric Power Hospital, Osaka, Japan; 104 Osaka Medical College Hospital, Takatsuki, Japan; 105 NHO Fukuoka-higashi Medical Center, Koga, Japan; 106 Tokushima Prefectural Central Hospital, Tokushima, Japan; 107 Yamaga Chuo Hospital, Yamaga, Japan; 108 NHO Kanagawa Hospital, Kanagawa, Japan; 109 Inuyama Chuo General Hospital, Inuyama, Japan; 110 Kindai University Hospital, Osakasayama, Japan; 111 Okayama University Hospital, Okayama, Japan; 112 Fukuoka Shin Mizumaki Hospital, Mizumaki, Japan; 113 Tobata Kyoritsu Hospital, Kitakyushu, Japan; 114 Kokura Kinen Hospital, Kitakyushu, Japan; 115 Fukuoka University Hospital, Fukuoka, Japan; 116 Iwate Prefectural Iwai Hospital, Ichinoseki, Japan; 117 Akita University Hospital, Akita, Japan; 118 Saitama Medical University Hospital, Moroyama, Japan; 119 Shizuoka City Shimizu Hospital, Shizuoka, Japan; 120 Aoyama General Hospital, Toyokawa, Japan; 121 Toyama Red Cross Hospital, Toyama, Japan; 122 Nanto Municipal Hospital, Nanto, Japan; 123 Kanazawa Nishi Hospital, Kanazawa, Japan; 124 Higashiosaka City Medical Center, Higashiosaka, Japan; 125 Ishinkai Yao General Hospital, Yao, Japan; 126 NHO Osaka National Hospital, Osaka, Japan; 127 Takarazuka City Hospital, Takarazuka, Japan; 128 Hyogo Prefectural Awaji Medical Center, Sumoto, Japan; 129 Onomichi Municipal Hospital, Onomichi, Japan; 130 Japanese Red Cross Fukuoka Hospital, Fukuoka, Japan; 131 Sasebo City General Hospital, Sasebo, Japan; 132 Obase Hospital, Kanda, Japan; 133 Miyakonojo Medical Assocciation Hospital, Miyakonojo, Japan; 134 Osaka Neurosurgical Hospital, Totonaka, Japan; 135 JOHAS Yokohama Rosai Hospital, Yokohama, Japan; 136 Ogachi Central Hospital, Yuzawa, Japan; 137 Kohnan Hospital, Sendai, Japan; 138 Ibaraki Prefectural Central Hospital, Kasama, Japan; 139 Itabashi Chuo Medical Center, Tokyo, Japan; 140 Shioda Hospital, Katsuura, Japan; 141 Japanese Red Cross Shizuoka Hospital, Shizuoka, Japan; 142 Japanese Red Cross Nagoya Daini Hospital, Nagoya, Japan; 143 Asuke Hospital, Toyota, Japan; 144 Saiseikai Matsusaka General Hospital, Matsusaka, Japan; 145 Shiga University of Medical Science Hospital, Otsu, Japan; 146 Iseikai Hospital, Osaka, Japan; 147 Nishiwaki Municipal Hospital, Nishiwaki, Japan; 148 Shimane Prefectural Central Hospital, Izumo, Japan; 149 Seiai Rehabilitation Hospital, Fukuioka, Japan; 150 Nishida Hospital, Saiki, Japan; 151 Urazoe General Hospital, Urazoe, Japan; 152 Shirakawa Hospital, Shirakawa, Japan; 153 Izumino Hospital, Bouchikai, Kochi, Japan; 154 Health Care System JINSEI-KAI Hosogi Hospital, Kochi, Japan; 155 Kushiro City General Hospital, Kushiro, Japan; 156 National Defense Medical College, Tokorozawa, Japan; 157 Keio University Hospital, Tokyo, Japan; 158 Nihon University School of Medicine, Tokyo, Japan; 159 Ohkubo Hospital, Tokyo, Japan; 160 Tokai University, Isehara, Japan; 161 Nagoya City East Medical Center, Nagoya, Japan; 162 Komaki City Hospital, Komaki, Japan; 163 Fukui Prefectural Hospital, Fukui, Japan; 164 Nagahama City Hospital, Nagahama, Japan; 165 Uji-Tokushukai Medical Center, Uji, Japan; 166 Hyogo Prefectural Nishinomiya Hospital, Nishinomiya, Japan; 167 Nishinomiya Kyouritsu Neurosurgical Hospital, Nishinomiya, Japan; 168 Kitakyushu General Hospital, Kitakyushu, Japan; 169 Shin Koga Hospital, Kurume, Japan; 170 Sato Daiichi Hospital, Usa, Japan; 171 Kagawa University Hospital, Miki, Japan; 172 Noichi Chuo Hospital, Konan, Japan; 173 JA Onomichi General Hospital, Onomichi, Japan; 174 Shinonoi General Hospital, Nagano, Japan; 175 Iwate Prefectural Kuji Hospital, Kuji, Japan; 176 Junwakai Memorial Hospital, Miyazaki, Japan; 177 Mominoki Hospital, Kochi, Japan; 178 Kyoto Okamoto Memorial Hospital, Kumiyama, Japan; 179 Obihiro-Kosei General Hospital, Obihiro, Japan; 180 NHO Hokkaido Medical Center, Sapporo, Japan; 181 Hakodate Neurosurgical Hospital, Hakodate, Japan; 182 Hokkaido University Graduate School of Medicine, Sapporo, Japan; 183 Sendai East Neurosurgical Hospital, Sendai, Japan; 184 Dokkyo Medical University, Mibu, Japan; 185 International Univesity of Healthcare and Welfare Hospital, Nasushiobara, Japan; 186 Japanese Red Cross Medical Center, Tokyo, Japan; 187 Toranomon Hospital, Tokyo, Japan; 188 Kitakurihama Neurosurgery, Yokosuka, Japan; 189 Gifu University Hospital, Gifu, Japan; 190 Niigata University Medical & Dental Hospital, Niigata, Japan; 191 Kanazawa Municipal Hospital, Kanazawa, Japan; 192 Kyoto University Hospital, Kyoto, Japan; 193 Osaka University Hospital, Suita, Japan; 194 Nara Medical University, Kashiwara, Japan; 195 Kawasaki Medical School, Kurashiki, Japan; 196 Saiseikai Yamaguchi Hospital, Yamaguchi, Japan; 197 Hakujuji Hospital, Sasebo, Japan; 198 Kida Hospital for Neurology, Pulmonology and Internal medicine, Shimabara, Japan; 199 Japanese Red Cross Kagoshima Hospital, Kagoshima, Japan; 200 Tokushima University Hospital, Tokushima, Japan; 201 Matsusaka Chuo General Hospital, Matsusaka, Japan; 202 Hakuai Hospital, Yonago, Japan; 203 Yamaguchi Prefectural Grand Medical Center, Hofu, Japan; 204 University of Tsukuba Hospital, Tsukuba, Japan; 205 Saiseikai Futsukaichi Hospital, Chikushino, Japan; 206 Takeda General Hospital, Takeda, Japan; 207 JA Toride Medical Center, Toride, Japan; 208 Jichi Medical University Hospital, Shimotsuke, Japan; 209 Saitama Medical Center, Saitama, Japan; 210 Tokyo Medical University Hospital, Tokyo, Japan; 211 NHO Tokyo National Hospital, Tokyo, Japan; 212 Tokai University Hachioji Hospital, Hachioji, Japan; 213 NHO Chiba Medical Center, Chiba, Japan; 214 St. Marianna University School of Medicine Toyoko, Kawasaki, Japan; 215 Fujiyoshida Municipal Medical Center, Fujiyoshida, Japan; 216 Hamamatsu Medical Center, Hamamatsu, Japan; 217 Hamamatsu University Hospital, Hamamatsu, Japan; 218 Nagoya Ekisaikai Hospital, Nagoya, Japan; 219 Tsushima City Hospital, Tsushima, Japan; 220 Nagoya City University Hospital, Nagoya, Japan; 221 Kariya Toyota General Hospital, Kariya, Japan; 222 Kuwana City Medical Center, Kuwana, Japan; 223 Toyama Rosai Hospital, Toyama, Japan; 224 JCHO Osaka Hospital, Osaka, Japan; 225 NIPPONBASHI Hospital, Osaka, Japan; 226 Kishiwada City Hospital, Kishiwada, Japan; 227 Takatsuki General Hospital, Takatsuki, Japan; 228 Kansai Medical University Hospital, Hirakata, Japan; 229 Wakayama Medical University, Wakayama, Japan; 230 Itami Kousei Neurosurgical Hospital, Itami, Japan; 231 Hiroshima University Hospital, Hiroshima, Japan; 232 Mazda Hospital, Huchu, Japan; 233 Omuta City Hospital, Omuta, Japan; 234 JCHO Fukuoka Yutaka Central Hospital, Nogata, Japan; 235 Social Insurance Inatsuki Hospital, Kama, Japan; 236 Saga-ken Medical Center Koseikan, Saga, Japan; 237 Kumamoto City Hospital, Kumamoto, Japan; 238 Seidoukai Kakamigahara Rehabilitation Hospital, Kakamigahara, Japan; 239 KKR Tachikawa Hospital, Tachikawa, Japan; 240 Saiseikai Hiroshima Hospital, Hiroshima, Japan; 241 Uchida Neurosurgery Hospital, Kochi, Japan; 242 IUHW Atami Hospital, Atami, Japan; 243 Tsukuba Medical Center Hospital, Tsukuba, Japan; 244 Tokyo Women’s Medical University Medical Center East, Tokyo, Japan; 245 Tomakomai City Hospital, Tomakomai, Japan; 246 Sapporo City General Hospital, Sapporo, Japan; 247 Asahikawa Medical University, Asahikawa, Japan; 248 Kyoujinkai Taisetsu Hospital, Asahikawa, Japan; 249 Hokuto Hospital, Obihiro, Japan; 250 Mito Kyodo General Hospital, Mito, Japan; 251 Tsuchiura Kyodo General Hospital, Tsuchiura, Japan; 252 Tochigi Medical Center, Tochigi, Japan; 253 Haga Red Cross Hospital, Haga, Japan; 254 Saiyu Soka Hospital, Soka, Japan; 255 Mitsui Memorial Hospital, Tokyo, Japan; 256 Tokyo Metropolitan Cancer and Infectious Diseases Center Komagome Hosptal, Tokyo, Japan; 257 Tokyo Medical and Dental University Medical Hospital, Tokyo, Japan; 258 Toho University Ohashi Medical Center, Tokyo, Japan; 259 Mishuku Hospital, Tokyo, Japan; 260 Kimitsu Chuo Hospital, Kimitsu, Japan; 261 Tokyo Women’s Medical University Yachiyo Medical Center Yachiyo, Yachiyo, Japan; 262 Shizuoka Medical Center, Shizuoka, Japan; 263 Tokai Central Hospital, Kakamigahara, Japan; 264 Kizawa Memorial Hospital, Minokamo, Japan; 265 Nagoya City West Medical Center, Nagoya, Japan; 266 JCHO Chukyo Hospital, Nagoya, Japan; 267 NHO Higashinagoya National Hospital, Nagoya, Japan; 268 Okazaki City Hospital, Okazak, Japan; 269 Toyohashi Medical Center, Toyohashi, Japan; 270 Midori Municipal Hospital, Nagoya, Japan; 271 Japanese Red Cross Maebashi Hospital, Maebashi, Japan; 272 Gunma University Hospital, Maebashi, Japan; 273 Nagano Municipal Hospital, Nagano, Japan; 274 Toyama Prefectural Central Hospital, Toyama, Japan; 275 Keiju Medical Center, Nanao, Japan; 276 Noto General Hospital, Nanao, Japan; 277 Kanazawa University Hospital, Kanazawa, Japan; 278 Kanazawa Medical University Hospital, Uchinada, Japan; 279 Japanese Red Cross Kyoto Daini Hospital, Kyoto, Japan; 280 Maizuru Medical Center, Maizuru, Japan; 281 Kyoto Chubu Medical Center, Nantan, Japan; 282 Ishikawa Prefectural Central Hospital, Kanazawa, Japan; 283 Rinku General Hospital Medical Center, Izumisano, Japan; 284 Osaka Minami Medical Center, Kawachinagano, Japan; 285 Matsushita Memorial Hospital, Moriguchi, Japan; 286 Hanwa Memorial Hospital, Osaka, Japan; 287 Kansai Rosai Hospital, Amagasaki, Japan; 288 Hiroshima Red Cross Hospital & Atomic-bomb Survivors Hospital, Hiroshima, Japan; 289 Kurume University Hospital, Kurume, Japan; 290 Fukuoka Dental College Medical & Dental Hospital, Fukuoka, Japan; 291 Kyushu University Hospital, Fukuoka, Japan; 292 Kumamoto University Hospital, Kumamoto, Japan; 293 Miyazaki Prefectural Miyazaki Hospital, Miyazaki, Japan; 294 Tokushima Prefecture Naruto Hospital, Naruto, Japan; 295 Japanese Red Cross Kochi Hospital, Kochi, Japan; 296 Kochi Medical School, Kochi, Japan; 297 Tannan Regional Medical Center, Sabae, Japan; 298 Sera Central Hospital, Sera, Japan; 299 Kumamoto Central Hospital, Kumamoto, Japan; 300 Nishitokyo Central General Hospital, Nishitokyo, Japan; 301 Tano Hospital, Tano, Japan; 302 Kitakasiwa Rehabilitation General Hospital, Kashiwa, Japan; 303 Tosa Municipal Hospital, Tosa, Japan; 304 Seirei Hamamatsu City Rehabilitation Hospital Hamamatsu, Hamamatsu, Japan; 305 Fukuoka Rehabilitation Hospital, Fukuoka, Japan; 306 Yokohama City Minato Red Cross Hospital, Yokohama, Japan

**Keywords:** Cerebral infarction, Hypertension, Stroke prevention, Cilostazol, Antiplatelet therapy

## Abstract

We determined the associations of follow-up blood pressure (BP) after stroke as a time-dependent covariate with the risk of subsequent ischemic stroke, as well as those of BP levels with the difference in the impact of long-term clopidogrel or aspirin monotherapy versus additional cilostazol medication on secondary stroke prevention. In a sub-analysis of a randomized controlled trial (CSPS.com), patients between 8 and 180 days after stroke onset were randomly assigned to receive aspirin or clopidogrel alone, or a combination of cilostazol with aspirin or clopidogrel. The percent changes, differences, and raw values of follow-up BP were examined. The primary efficacy outcome was the first recurrence of ischemic stroke. In a total of 1657 patients (69.5 ± 9.3 years, female 29.1%) with median 1.5-year follow-up, ischemic stroke recurred in 74 patients. The adjusted hazard ratio for ischemic stroke of a 10% systolic BP (SBP) increase from baseline was 1.19 (95% CI 1.03–1.36), that of a 10 mmHg SBP increase was 1.14 (1.03–1.28), and that of SBP as the raw value with the baseline SBP as a fixed (time-independent) covariate was 1.14 (1.00–1.31). Such significant associations were not observed in diastolic BP-derived variables. The estimated adjusted hazard ratio curves for the outcome showed the benefit of dual therapy over a wide SBP range between ≈120 and ≈165 mmHg uniformly. Lower long-term SBP levels after ischemic stroke were associated with a lower risk of subsequent ischemic events. The efficacy of dual antiplatelet therapy including cilostazol for secondary stroke prevention was evident over a wide SBP range.

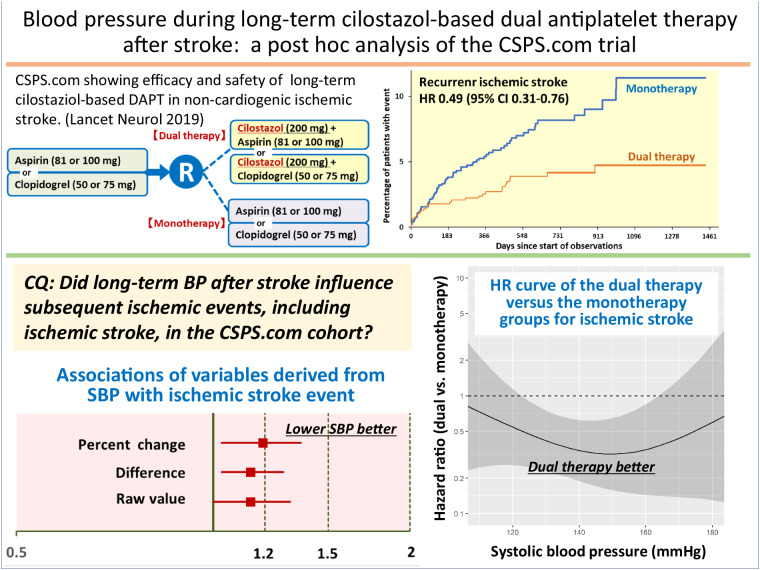

## Introduction

Long-term treatment with cilostazol, a phosphodiesterase 3 inhibitor, coupled with aspirin or clopidogrel was shown to halve the risk of recurrent ischemic stroke and have a similar risk of severe or life-threatening bleeding compared to aspirin or clopidogrel alone in patients at high risk for recurrent noncardioembolic ischemic stroke in a randomized, controlled trial, the Cilostazol Stroke Prevention Study for Antiplatelet Combination (CSPS.com) [1]. Cilostazol is known to have a lower risk of bleeding than the other antiplatelet agents [2, 3], and the result of the safety outcome was within the expectations of the trial investigators. In contrast, the strong efficacy of the dual antiplatelet therapy (DAPT) with half the risk of recurrent ischemic stroke exceeded our assumptions to some extent. The possible clinical conditions causing such clear efficacy need clarification.

The post-stroke blood pressure (BP) level is predictive of recurrent stroke risk [4–7]. Several guidelines recommend intensive BP lowering for chronic stroke to prevent recurrent stroke [8–10]. Appropriate risk-factor modification would strengthen the preventive power of antithrombotic pharmacotherapy. Thus, BP control might have affected the main results of CSPS.com.

The primary research objective of the present sub-study was to determine the associations of long-term BP after stroke as a time-dependent covariate with the risk of subsequent ischemic events, including recurrent ischemic stroke, in the CSPS.com cohort. The secondary objective was to clarify the associations of follow-up BP level with the therapeutic superiority of cilostazol-based DAPT to monotherapy

Point of view

- Clinical relevance
  Lower long-term systolic blood pressure levels during single antiplatelet therapy or cilostazol-based dual antiplatelet therapy in noncardioembolic ischemic stroke patients were associated with a lower risk of subsequent ischemic events. Cilostazol-based dual antiplatelet therapy was superior to single antiplatelet therapy in secondary stroke prevention over a wide systolic blood pressure range.
- Future direction
  The positive results of promising trial treatments would be more clearly identified when the vascular risk factors of the participants, including hypertension, are appropriately controlled.
- Consideration for the Asian population
  Secondary stroke prevention using cilostazol, that is popular in Asia, can be more effective by optimal blood pressure control.

## Methods

### Study design and setting

CSPS.com was a multicenter, randomized, open-label, parallel-group trial, involving participants from 292 sites across Japan registered from December 2013 through March 2017. The trial protocol, statistical analysis plan, design, and main and major post hoc results of CSPS.com have been reported previously [1, 11–15]. CSPS.com was registered in ClinicalTrials.gov NCT01995370 and the University Hospital Medical Information Network clinical trial registry in Japan UMIN 000012180 and approved by the ethics committee at each participating site. All patients gave written, informed consent before randomization. This study followed the Consolidated Standards of Reporting Trials (CONSORT) reporting guidelines.

### Participants

Eligible patients were between 20 and 85 years of age who had a non-cardioembolic ischemic stroke identified on magnetic resonance imaging between 8 and 180 days before the start of the protocol treatment and were taking either aspirin or clopidogrel alone as antiplatelet therapy when providing informed consent. The patients were required to meet at least one of the following three criteria indicating a high risk for stroke recurrence: (a) ≥ 50% stenosis of a major intracranial artery; (b) ≥ 50% stenosis of an extracranial artery; and (c) two or more of the following risk factors (age ≥ 65 years, hypertension, diabetes mellitus, chronic kidney disease, peripheral arterial disease, history of ischemic stroke other than the qualifying one for this trial, history of ischemic heart disease, and current smoking). Additional information regarding the inclusion and exclusion criteria is provided elsewhere [1, 11]. For example, patients with emboligenic heart disease were excluded from the study. In this sub-study, patients with consistent BP data at baseline and at least once during the follow-up period were included.

Patients were randomly assigned in a 1:1 ratio to receive either monotherapy with aspirin (81 or 100 mg) or clopidogrel (50 or 75 mg) once daily or dual therapy with cilostazol (100 mg, twice daily, the recommended dose for stroke prevention in Japan) and either aspirin (81 or 100 mg) or clopidogrel (50 or 75 mg), once daily. Trial medication was continued for half a year or longer, for a maximum of 3.5 years. Changes in these three antiplatelet medications were not permitted after informed consent was obtained. Systolic and diastolic BPs (SBP, DBP) were measured in a sitting position at follow-up visits 1, 3, and 6 months later and every 6 months thereafter.

### Outcomes

The primary efficacy outcome was the first recurrence of ischemic stroke. The secondary efficacy outcome was the first occurrence of a composite of stroke, myocardial infarction, and vascular death. The safety outcome was the first occurrence of severe or life-threatening bleeding as defined in the Global Utilization of Streptokinase and Tissue Plasminogen Activator for Occluded Coronary Arteries classification [16]. These events have been defined elsewhere [1, 11].

### Statistical analysis

Continuous data are reported as medians (interquartile range) or means ± standard deviation, and categorical data are presented as numbers (%). In principle, all BP data were treated as time-dependent covariates. The variables “percent change” and “difference” were derived as the ratio of BP values to baseline BP value and the difference from baseline at each time point, respectively. Note that these variables are still time-dependent covariates.

For example, consider a patient with a baseline BP value of 140 mmHg and follow-up BP values of 150, 140, and 130 mmHg. In the “raw value” analysis, the follow-up values of 150, 140, and 130 mmHg are directly used, with the baseline value of 140 mmHg serving as a fixed (time-independent) adjustment variable. In the “difference” analysis, the changes from the baseline are calculated as 10, 0, and −10 mmHg, respectively. For the “percent change” analysis, the converted values are computed as follows: (150/140)*100, (140/140)*100 and (130/140)*100, yielding percentages of 107%, 100%, and 93% respectively.

A Cox proportional hazards model was used to calculate adjusted hazard ratios (aHRs) and 95% confidence intervals (CIs) of the percent changes, differences, and raw values of BP. For the analysis of raw values, follow-up BP values were treated as time-dependent covariates, while baseline BP value was included as time-independent covariate. HRs are represented as per 10% for percent change and per 10 mmHg for difference and raw value. Multivariable adjustment was performed using sex, age, and variables that were proven to affect the outcomes of CSPS.com, including assigned treatment, antiplatelet agents at randomization, intracranial artery stenosis, and the timing of starting trial treatment [11–14]. The preventive effect of dual therapy relative to monotherapy on outcomes considering its interaction with BP levels was visualized using a generalized additive model. In brief, the hazard function was estimated through a generalized additive model of Poisson regression, which allows for flexible modeling of complex nonlinear relationships, and calculated aHR and 95% CI values were plotted against BP. All statistical analyses were performed using R version 4.2.0 (R Core Team 2022). Fitting the generalized additive model was done with pammtools and mgcv R packages.

## Results

**Fig. 1:**
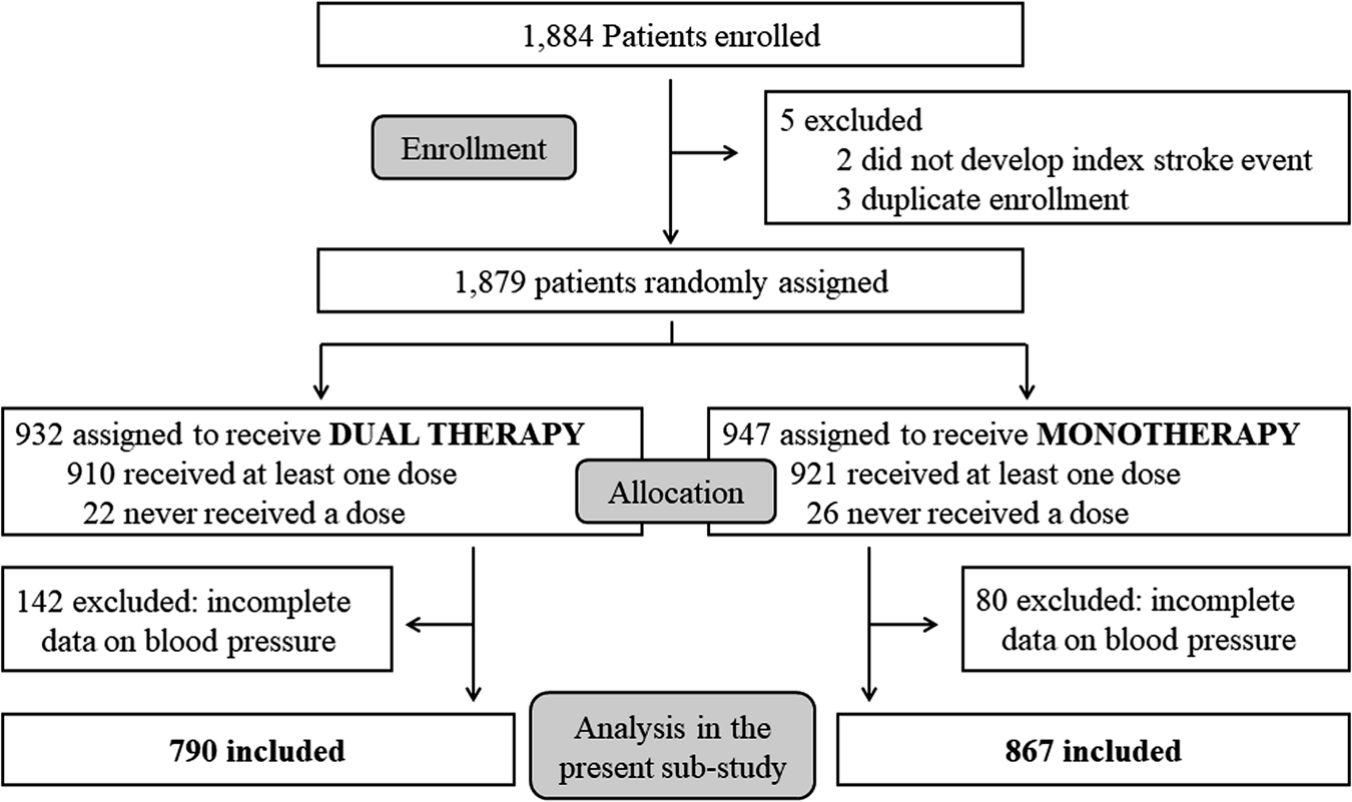
Trial profile

**Fig. 2:**
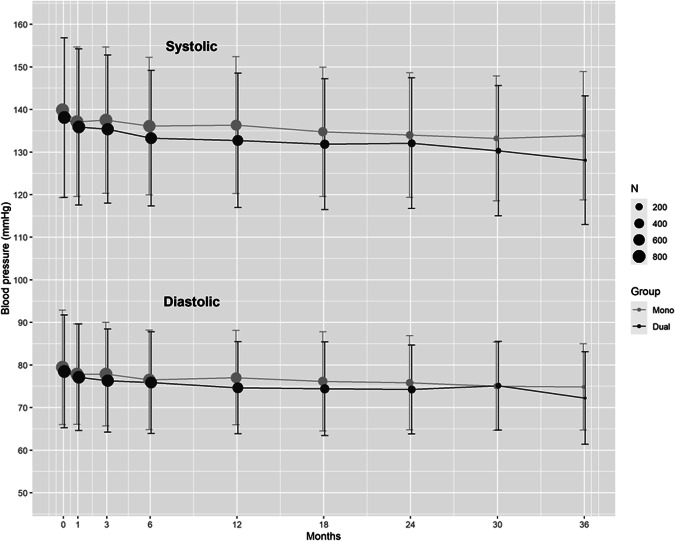
Blood pressure at each visit

Of the total 1879 randomized patients, 222 were excluded from the present study due to lack of sufficient and consistent data on BP (or unclear description between the timing of events onset and that of follow-up BP measurement), and 1657 were finally studied; 790 were assigned to dual therapy and 867 to monotherapy (Fig. 1). Table 1 shows the patients’ baseline characteristics. Overall, average BP was 139.0 ± 19.7/79.0 ± 13.4 mmHg at baseline and 136.1 ± 12.6/77.0 ± 9.2 mmHg at mean follow-up. The median duration of follow-up was 1.5 years overall (interquartile range 1.0–2.3 years), resulting in 2700.9 person-years of follow-up. Ischemic stroke occurred in 74 patients, the composite of stroke, myocardial infarction, and vascular death occurred in 92 patients, and severe or life-threatening bleeding occurred in 18 patients. All severe or life-threatening bleeding events were symptomatic intracranial hemorrhages. Figure 2 shows BP levels at each visit. The median number of BP measurements per patient was 5 (interquartile range: 4–7).

**Table 1:** Patients’ baseline characteristics and blood pressure levels

	Total	Monotherapy	Dual therapy
*N*	1,657	867	790
Age, y	69.5 ± 9.3	69.6 ± 9.2	69.5 ± 9.3
Female sex	482 (29.1)	237 (29.3)	245 (31.0)
Asian ^a^	1,657 (100)	867 (100)	790 (100)
Body mass index, kg/m^2^	23.8 ± 3.5	23.7 ± 3.4	23.9 ± 3.6
Medical history			
Hypertension	1,419 (85.6)	740 (85.4)	679 (85.9)
Dyslipidemia	935 (56.4)	501 (57.8)	434 (54.9)
Diabetes mellitus	632 (38.1)	331 (38.2)	301 (38.1)
Chronic kidney disease	100 (6.0)	46 (5.3)	54 (6.8)
Peripheral arterial disease	46 (2.8)	21 (2.4)	25 (3.2)
History of ischemic stroke^b^	246 (14.8)	136 (15.7)	110 (13.9)
History of ischemic heart disease	87 (5.3)	44 (5.1)	43 (5.4)
Current smoking	476 (28.7)	254 (29.3)	222 (28.1)
Intracranial artery stenosis	493 (29.8)	256 (29.5)	237 (30.0)
Extracranial artery stenosis	236 (14.2)	131 (15.1)	105 (13.3)
Antihypertensive medication			
Calcium channel blockers	817 (49.3)	427 (49.3)	390 (49.4)
Angiotensin-converting enzyme inhibitors/angiotensinb receptor blockers	815 (49.2)	425 (49.0)	390 (49.4)
β-blockers, α/β-blockers	93 (5.6)	43 (5.0)	50 (6.3)
Diuretics	79 (4.8)	46 (5.3)	33 (4.2)
Others	37 (2.2)	21 (2.4)	16 (2.0)
Clopidogrel at randomization	973 (58.7)	518 (59.7)	455 (57.6)
Stroke subtype			
Lacunar	825 (49.8)	427 (49.3)	398 (50.4)
Atherothrombotic	717 (43.3)	375 (43.3)	342 (43.3)
Infarct location			
Supratentorial (purely)	1,243 (75.0)	649 (74.9)	594 (75.2)
Infratentorial (purely)	394 (23.8)	204 (23.5)	190 (24.1)
Both	20 (1.2)	14 (1.6)	6 (0.8)
Time to randomization after index event, days	27 [14–63]	26 [14-62.5]	27 [14–63]
Blood pressure levels, mmHg			
Baseline, systolic	139.0 ± 19.7	139.9 ± 20.6	138.1 ± 18.7
Baseline, diastolic	79.0 ± 13.4	79.5 ± 13.4	78.5 ± 13.2
Mean follow-up, systolic	136.1 ± 12.6	137.1 ± 12.6	135.1 ± 12.6
Mean follow-up, diastolic	77.0 ± 9.2	77.5 ± 9.2	76.5 ± 9.3

Data are numbers (%), medians [interquartile range], or means ± SD

^a^Self-reported

^b^Except the qualifying one for this trial

### Primary outcome of ischemic stroke

Table 2 shows the associations of BP-derived variables with recurrent ischemic stroke. When BP was treated as a time-dependent covariate, the aHR of a 10% SBP increase from baseline was 1.19 (95% CI: 1.03–1.36), and that of a 10 mmHg SBP increase from baseline was 1.14 (1.03–1.28). With the baseline SBP as a fixed (time-independent) covariate, the aHR of SBP was 1.14 (1.00–1.31) per 10 mmHg. On the other hand, no DBP-derived variables showed significant associations with the outcome.

**Table 2:** Associations of variables derived from blood pressure with outcomes

		Crude		Adjusted	
		Hazard ratio (95% confidence interval)	*P*	Hazard ratio (95% confidence interval)	*P*
***Ischemic stroke***					
**Systolic**	Percent change	1.21 (1.05–1.39)	< 0.01	1.19 (1.03–1.36)	0.02
	Difference	1.16 (1.04–1.29)	< 0.01	1.14 (1.03–1.28)	0.01
	Raw value	1.17 (1.02–1.35)	0.02	1.14 (1.00–1.31)	0.06
**Diastolic**	Percent change	1.07 (0.95–1.21)	0.26	1.07 (0.94–1.21)	0.31
	Difference	1.11 (0.94–1.32)	0.21	1.10 (0.93–1.30)	0.26
	Raw value	1.06 (0.86–1.31)	0.57	1.08 (0.87–1.33)	0.50
***Composite events***					
**Systolic**	Percent change	1.20 (1.06–1.36)	< 0.01	1.18 (1.04–1.34)	< 0.01
	Difference	1.16 (1.05–1.28)	< 0.005	1.15 (1.04–1.26)	< 0.01
	Raw value	1.17 (1.03–1.33)	0.01	1.15 (1.01–1.30)	0.03
**Diastolic**	Percent change	1.09 (0.98–1.22)	0.12	1.09 (0.98–1.22)	0.13
	Difference	1.14 (0.98 – 1.32)	0.10	1.13 (0.97–1.32)	0.11
	Raw value	1.14 (0.94–1.37)	0.19	1.14 (0.95–1.38)	0.17
***Intracranial hemorrhage***					
**Systolic**	Percent change	1.11 (0.82 –1.50)	0.51	-	-
	Difference	1.09 (0.87–1.37)	0.44	-	-
	Raw value	1.10 (0.82–1.46)	0.53	-	-
**Diastolic**	Percent change	0.91 (0.68–1.20)	0.50	-	-
	Difference	0.91 (0.64–1.28)	0.58	-	-
	Raw value	1.01 (0.66–1.57)	0.95	-	-

Cox proportional hazards model

Per 10% for “percent change” and per 10 mmHg for “difference” and “raw value”

Baseline blood pressure value was included as time-independent covariate for the analysis of “raw value”.

Adjusted by sex, age, assigned treatment, antiplatelet agents at randomization, intracranial artery stenosis, the time of starting trial treatment

Variables of diastolic blood pressure are analyzed using 1655 patients due to the lack of baseline diastolic blood pressure values for 2 patients

Adjusted analysis of intracranial hemorrhage was not performed due to the small event number

Table 3 shows the associations of SBP-derived variables with recurrent ischemic stroke in subgroups by assigned antiplatelet therapy and those by the antiplatelet agent at randomization. Similar numerical data with those in the overall patients were obtained in all subgroups, with significant associations of the percent change and the difference in SBP in the monotherapy subgroup and the clopidogrel subgroup. Note that the event rate was somewhat higher in patients taking clopidogrel (50/973) than those taking aspirin (24/684). The difference was probably because the physicians preferred clopidogrel to aspirin for patients with history of ischemic stroke and those with intra-/extracranial artery stenosis, that showed clearly higher risk of recurrent ischemic stroke [1, 12].

**Table 3:** Associations of variables derived from systolic blood pressure with ischemic stroke

	Crude		Adjusted	
	Hazard ratio (95% confidence interval)	*P*	Hazard ratio (95% confidence interval)	*P*
Assigned to receive monotherapy (867 patients, 54 events of ischemic stroke)				
Percent change	1.20 (1.02–1.42)	0.03	1.19 (1.00–1.40)	0.04
Difference	1.15 (1.02–1.31)	0.03	1.14 (1.00–1.30)	0.04
Raw value	1.17 (0.99–1.38)	0.06	1.16 (0.98–1.36)	0.08
Assigned to receive dual therapy (790 patients, 20 events)				
Percent change	1.21 (0.92–1.59)	0.16	1.19 (0.92–1.54)	0.19
Difference	1.17 (0.94–1.44)	0.16	1.16 (0.94–1.42)	0.16
Raw value	1.13 (0.87–1.48)	0.35	1.11 (0.85–1.45)	0.46
Clopidogrel at randomization (973 patients, 50 events)				
Percent change	1.24 (1.05 –1.46)	0.01	1.22 (1.03–1.44)	0.02
Difference	1.18 (1.04–1.35)	0.01	1.17 (1.02–1.34)	0.02
Raw value	1.19 (1.00 – 1.41)	0.05	1.16 (0.98–1.38)	0.09
Aspirin at randomization (684 patients, 24 events)				
Percent change	1.13 (0.87–1.46)	0.35	1.13 (0.88–1.46)	0.34
Difference	1.10 (0.91–1.33)	0.33	1.10 (0.91–1.33)	0.31
Raw value	1.13 (0.90–1.43)	0.29	1.10 (0.87–1.38)	0.42

Cox proportional hazards model

Per 10% for “percent change” and per 10 mmHg for “difference” and “raw value”

Baseline blood pressure value was included as time-independent covariate for the analysis of “raw value”

Adjusted by sex, age, assigned treatment, antiplatelet agents at randomization, intracranial artery stenosis, the time of starting trial treatment

**Fig. 3:**
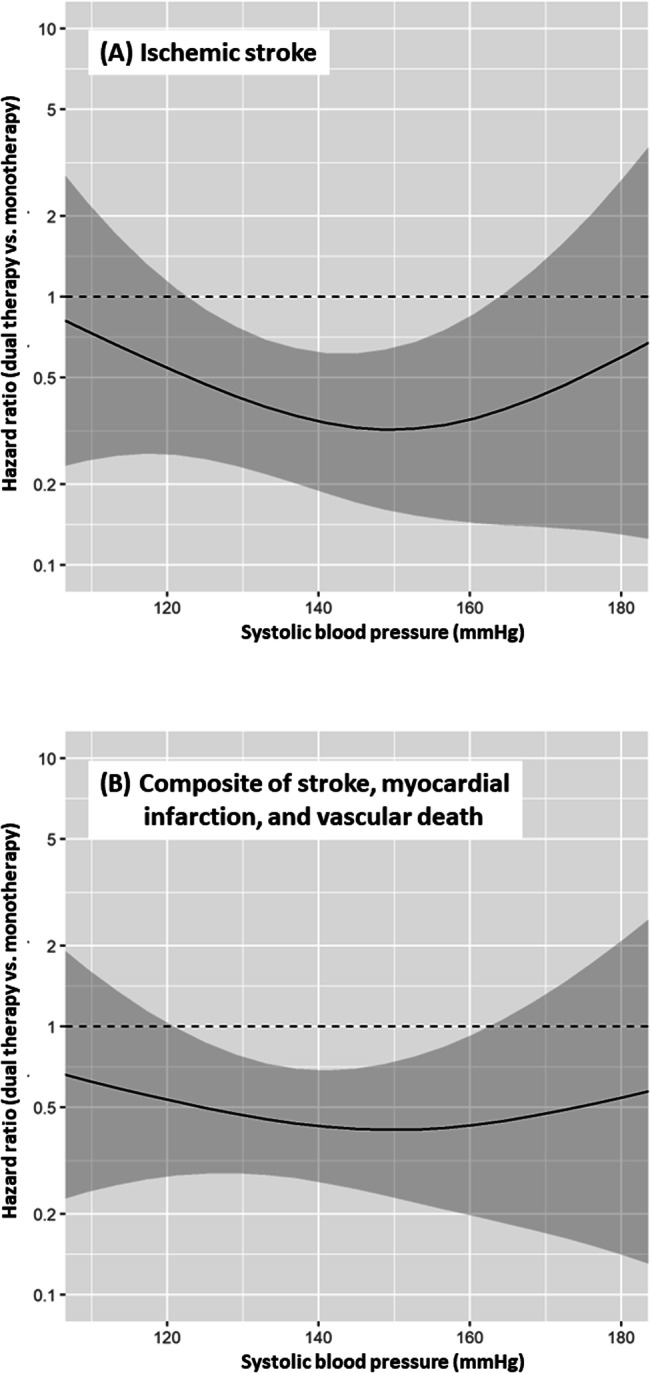
Hazard ratio curve of the dual therapy group versus the monotherapy group. **A** Ischemic stroke, **B** a composite of stroke, myocardial infarction, and vascular death. Adjusted by sex, age, assigned treatment, antiplatelet agents at randomization, intracranial artery stenosis, and the time of starting trial treatment

Fig. 3A shows the aHR of dual therapy relative to monotherapy plotted against SBP. The estimated aHR curve for the outcome showed the benefit of dual therapy over a wide SBP range from ≈120 mmHg to ≈165 mmHg uniformly, with the greatest risk reduction when SBP was ≈150 mmHg.

### Other outcomes

The aHR for the secondary outcome of composite events of a 10% and a 10 mmHg SBP increase from baseline was 1.18 (1.04–1.34) and 1.15 (1.04–1.26), respectively (Table 2). With the baseline SBP as a fixed covariate, the aHR of SBP was 1.15 (1.01–1.30). No DBP-derived variables showed significant associations with the outcome. The estimated aHR curve for the outcome showed the benefit of dual therapy relative to monotherapy over a wide SBP range from ≈120 mmHg to ≈160 mmHg uniformly (Fig. 3B).

No SBP-derived or DBP-derived variables showed significant associations with the safety outcome of severe or life-threatening bleeding (Table 2).

## Discussion

This was a sub-analysis of the randomized CSPS.com trial to determine the effects of follow-up BP parameters as a time-dependent covariate on recurrent stroke risk. The first major finding of this study was that both ischemic stroke and composite cardiovascular events increased significantly as the variables derived from SBP increased, but not as the DBP-derived variables increased. The second major finding was that the benefit of dual therapy was uniformly observed over a wide SBP range, with the greatest risk reduction when SBP was ≈150 mmHg.

Cilostazol inhibits phosphodiesterase activity and suppresses cyclic adenosine monophosphate (cAMP) degradation, increases intracellular cAMP concentrations, activates the cAMP-dependent protein kinase A, and inhibits platelet aggregation [2, 3, 17]. Both cilostazol monotherapy and cilostazol-based long-term DAPT were recommended as the first-line antiplatelet agents for secondary stroke prevention in Japan [10] and several Asian countries based on the results of CSPS2 and CSPS.com [1, 18]. Cilostazol monotherapy was weakly recommended for stroke or transient ischemic attack attributable to moderate to severe intracranial artery stenosis in the United States of America [19]. Cilostazol has pleiotropic effects other than inhibiting platelet aggregation, such as a vasodilatory effect on smooth muscle cells via the increase in intracellular cAMP concentrations [20]. The vasodilation may cause mild BP lowering; a 2 to 4 mmHg lower SBP level was observed during cilostazol-based DAPT than during monotherapy throughout the follow-up period in the overall CSPS.com cohort [1].

Lower percent changes, differences, and raw values of SBP were associated with lower risks of ischemic stroke. This “the lower, the better” phenomenon in stroke survivors was common to the results of the Perindopril Protection Against Recurrent Stroke Study (PROGRESS) [4, 7], but different from those of the Prevention Regimen for Effectively Avoiding Second Strokes (PRoFESS) study, where the risk was higher for patients with SBP < 120 mmHg [21]. Since patients within 120 days after stroke were enrolled in PRoFESS and those within 5 years were enrolled in PROGRESS, failure of cerebral autoregulation during subacute stroke might have affected the results more in PRoFESS. In addition, the Secondary Prevention of Small Subcortical Strokes (SPS3) and Recurrent Stroke Prevention Clinical Outcome (RESPECT) trials showed a significant risk reduction of intracerebral hemorrhage in patients with a target SBP < 130 mm Hg or < 120 mmHg compared with a higher target but did not show the significant reduction of ischemic stroke [22–24]. A reason for the differences in results between these trials and ours is that in our study, SBP was treated as a time-dependent covariate, and our findings were not confined to a specific SBP range, such as around 130 mmHg. Additionally, the discrepancies may be due to the fact that patients’ SBP data were treated consecutively in our study, without grouping patients based on a specific SBP value. Another possible explanation was the higher percentage of patients assigned to dual therapy in the lower mean SBP population, since dual therapy excessively lowered SBP by 2 to 4 mmHg.

In contrast, the DBP-derived variables were not associated with the outcome. The J-shaped relationship between DBP and adverse cardiovascular outcomes including stroke with DBP nadir around 70–80 mmHg has been repeatedly reported [25–30]. The J-shaped curve might be due to the positive association of low DBP and evident or subclinical myocardial damage [31] and due to the high frequency of isolated systolic hypertension with an increased pulse pressure in the low DBP cohort [30, 32]. However, the presence of a J-shaped relationship between DBP and the risk of stroke alone was somewhat unclear [25, 26, 31], presumably partly due to differences in mechanisms between cerebral and coronary autoregulation [33].

The benefit of dual therapy was observed over a wide SBP range. This suggests a simple message: the positive results of promising trial treatments can be more clearly identified when the vascular risk factors of the participants are appropriately controlled. Patients with good BP management might also be mindful of their general health condition and modify risk factors other than hypertension, and thus the impact of dual therapy might be clearer in such patients.

Visit-to-visit SBP variability was reported to be firmly associated with stroke outcomes and subsequent events [34–36]. Since the number of visits in the present patients was small (median 5), the association between BP variability and outcomes was not examined.

The strength of this study was that BP was treated as a time-dependent covariate and evaluated over time. For example, summarizing a patient’s BP values into a single statistic, e.g., mean BP, and treating it as a time-independent covariate would be equivalent to assuming that the patient’s BP is constant at the mean BP level throughout the follow-up period. However, this may miss the more detailed relationship between BP and events. The present method takes into account the fact that a patient’s BP is not constant and can change during the follow-up period, allowing for a more detailed and relevant assessment.

The limitations of the present study include the post hoc nature of the analysis, meaning that the associations identified might not necessarily imply causality. Severe stroke could cause both subsequent events and higher BP values. Second, visits for BP measurement were not frequent for patients developing events early after enrollment. Third, the present sample size seemed to be small for appropriate subgroup analyses, although numerical results indicated a tendency for a positive association between SBP and subsequent ischemic stroke regardless of assigned antiplatelet therapy and the antiplatelet agent at randomization. Fourth, the number of severe or life-threatening bleeding events (intracranial hemorrhage) was too small to perform meaningful statistical analysis. Hemorrhagic stroke generally shows a stronger association with follow-up BP than ischemic stroke [7, 37, 38].

### Perspective of Asia

In Asia, cilostazol is widely used for secondary stroke prevention. DAPT including cilostazol decreased the risk of recurrent ischemic stroke compared to aspirin or clopidogrel monotherapy in patients with lacunar stroke and those with intracranial arterial stenosis [15, 39]; both of these pathologies are common to Asian population. BP lowering after stroke is an optimal strategy to strengthen the power of cilostazol-based DAPT in Asian stroke patients.

## Conclusion

The present sub-study renewed our understanding of the importance of intensive BP lowering for secondary stroke prevention and also suggested the necessity of appropriate BP control for success in stroke clinical trials.

## Supplementary information

Supplementary Materials

Supplementary information

## References

[1] Toyoda K, Uchiyama S, Yamaguchi T, Easton JD, Kimura K, Hoshino H, et al. Dual antiplatelet therapy using cilostazol for secondary prevention in patients with high-risk ischaemic stroke in Japan: a multicentre, open-label, randomised controlled trial. Lancet Neurol. 2019;18:539–48. 31122494 10.1016/S1474-4422(19)30148-6

[2] De Havenon A, Sheth K, Madsen T, Johnston KC, Turan TN, Toyoda K, et al. Cilostazol for secondary stroke prevention: history, evidence, limitations, and possibilities. Stroke. 2021;52:e635–e645. 34517768 10.1161/STROKEAHA.121.035002 PMC8478840

[3] Kherallah RY, Khawaja M, Olson M, Angiolillo D, Birnbaum Y. Cilostazol: a review of basic mechanisms and clinical uses. Cardiovasc Drugs Ther. 2022;36:777–92. 33860901 10.1007/s10557-021-07187-x

[4] PROGRESS Collaborative Group. Randomised trial of a perindopril-based blood pressure lowering regimen among 6,105 individuals with previous stroke or transient ischaemic attack. Lancet. 2001;358:1033–41. 11589932 10.1016/S0140-6736(01)06178-5

[5] Friday G, Alter M, Lai SM. Control of hypertension and risk of stroke recurrence. Stroke. 2002;33:2652–7. 12411656 10.1161/01.STR.0000033929.62136.6F

[6] Rashid P, Leonardi-Bee J, Bath P. Blood pressure reduction and secondary prevention of stroke and other vascular events: a systematic review. Stroke. 2003;34:2741–8. 14576382 10.1161/01.STR.0000092488.40085.15

[7] Arima H, Chalmers J, Woodward M, Anderson C, Rodgers A, Davis S, et al. Lower target blood pressures are safe and effective for the prevention of recurrent stroke: the PROGRESS trial. J Hypertens. 2006;24:1201–8. 16685221 10.1097/01.hjh.0000226212.34055.86

[8] Whelton PK, Carey RM, Aronow WS, Casey DE Jr, Collins KJ, Dennison Himmelfarb C, et al. 2017 ACC/AHA/AAPA/ABC/ACPM/AGS/APhA/ASH/ASPC/NMA/PCNA Guideline for the Prevention, Detection, Evaluation, and Management of High Blood Pressure in Adults: Executive Summary: A Report of the American College of Cardiology/American Heart Association Task Force on Clinical Practice Guidelines. J Am Coll Cardiol. 2018;71:2199–269. 29146533 10.1016/j.jacc.2017.11.005

[9] Williams B, Mancia G, Spiering W, Agabiti Rosei E, Azizi M, Burnier M, et al. 2018 ESC/ESH Guidelines for the management of arterial hypertension. Eur Heart J. 2018;39:3021–104. 30165516 10.1093/eurheartj/ehy339

[10] Miyamoto S, Ogasawara K, Kuroda S, Itabashi R, Toyoda K, Itoh Y, et al. Japan Stroke Society Guideline 2021 for The Treatment of Stroke. Int J Stroke. 2022;17:1039–49. 35443847 10.1177/17474930221090347 PMC9615334

[11] Toyoda K, Uchiyama S, Hoshino H, Kimura K, Origasa H, Naritomi H, et al. Protocol for Cilostazol Stroke Prevention Study for Antiplatelet Combination (CSPS.com): a randomized, open-label, parallel-group trial. Int J Stroke. 2015;10:253–8. 25487817 10.1111/ijs.12420 PMC4335602

[12] Hoshino H, Toyoda K, Omae K, Ishida N, Uchiyama S, Kimura K, et al. Dual antiplatelet therapy using cilostazol with aspirin or clopidogrel: subanalysis of the CSPS.com trial. Stroke. 2021;52:3430–9. 34404237 10.1161/STROKEAHA.121.034378 PMC8547582

[13] Toyoda K, Omae K, Hoshino H, Uchiyama S, Kimura K, Miwa K, et al. Association of timing for starting dual antiplatelet treatment with cilostazol and recurrent stroke: a CSPS.com trial post hoc analysis. Neurology. 2022;98:e983–e992. 35074890 10.1212/WNL.0000000000200064 PMC8967394

[14] Uchiyama S, Toyoda K, Okamura S, Omae K, Hoshino H, Kimura K, et al. Dual antiplatelet therapy with cilostazol in stroke patients with extracranial arterial stenosis or without arterial stenosis: A subgroup analysis of the CSPS.com trial. Int J Stroke. 2023;18:426–32. 35762581 10.1177/17474930221112343

[15] Nishiyama Y, Kimura K, Otsuka T, Toyoda K, Uchiyama S, Hoshino H, et al. Dual antiplatelet therapy with cilostazol for secondary prevention in lacunar stroke: subanalysis of the CSPS.com trial. Stroke. 2023;54:697–705. 36734235 10.1161/STROKEAHA.122.039900

[16] The GUSTO investigators. An international randomized trial comparing four thrombolytic strategies for acute myocardial infarction. N. Engl J Med. 1993;329:673–82. 8204123 10.1056/NEJM199309023291001

[17] Mchutchison C, Blair GW, Appleton JP, Chappell FM, Doubal F, Bath PM, et al. Cilostazol for secondary prevention of stroke and cognitive decline: systematic review and meta-analysis. Stroke. 2020;51:2374–85. 32646330 10.1161/STROKEAHA.120.029454 PMC7382534

[18] Shinohara Y, Katayama Y, Uchiyama S, Yamaguchi T, Handa S, Matsuoka K, et al. Cilostazol for prevention of secondary stroke (CSPS 2): an aspirin-controlled, double-blind, randomised non-inferiority trial. Lancet Neurol. 2010;9:959–68. 20833591 10.1016/S1474-4422(10)70198-8

[19] Kernan WN, Ovbiagele B, Black HR, Bravata DM, Chimowitz MI, Ezekowitz MD, et al. Guidelines for the prevention of stroke in patients with stroke and transient ischemic attack. Stroke. 2014;45:2160–236. 24788967 10.1161/STR.0000000000000024

[20] Nakamura T, Houchi H, Minami A, Sakamoto S, Tsuchiya K, Niwa Y, et al. Endothelium-dependent relaxation by cilostazol, a phosphodiesteras III inhibitor, on rat thoracic aorta. Life Sci. 2001;69:1709–15. 11665832 10.1016/S0024-3205(01)01258-9

[21] Ovbiagele B, Diener HC, Yusuf S, Martin RH, Cotton D, Vinisko R, et al. Level of systolic blood pressure within the normal range and risk of recurrent stroke. JAMA. 2011;306:2137–44. 22089721 10.1001/jama.2011.1650

[22] Benavente OR, Coffey CS, Conwit R, Hart RG, Mcclure LA, Pearce LA, et al. Blood-pressure targets in patients with recent lacunar stroke: the SPS3 randomised trial. Lancet. 2013;382:507–15. 23726159 10.1016/S0140-6736(13)60852-1 PMC3979302

[23] Kitagawa K, Yamamoto Y, Arima H, Maeda T, Sunami N, Kanzawa T, et al. Effect of standard vs intensive blood pressure control on the risk of recurrent stroke: a randomized clinical trial and meta-analysis. JAMA Neurol. 2019;76:1309–18. 31355878 10.1001/jamaneurol.2019.2167 PMC6664380

[24] Kitagawa K, Arima H, Yamamoto Y, Ueda S, Rakugi H, Kohro T, et al. Intensive or standard blood pressure control in patients with a history of ischemic stroke: RESPECT post hoc analysis. Hypertens Res. 2022;45:591–601. 35241817 10.1038/s41440-022-00862-y

[25] Hansson L, Zanchetti A, Carruthers SG, Dahlöf B, Elmfeldt D, Julius S, et al. Effects of intensive blood-pressure lowering and low-dose aspirin in patients with hypertension: principal results of the Hypertension Optimal Treatment (HOT) randomised trial. HOT Study Group. Lancet. 1998;351:1755–62. 9635947 10.1016/S0140-6736(98)04311-6

[26] Messerli FH, Mancia G, Conti CR, Hewkin AC, Kupfer S, Champion A, et al. Dogma disputed: can aggressively lowering blood pressure in hypertensive patients with coronary artery disease be dangerous? Ann Intern Med. 2006;144:884–93. 16785477 10.7326/0003-4819-144-12-200606200-00005

[27] Vidal-Petiot E, Ford I, Greenlaw N, Ferrari R, Fox KM, Tardif JC, et al. Cardiovascular event rates and mortality according to achieved systolic and diastolic blood pressure in patients with stable coronary artery disease: an international cohort study. Lancet. 2016;388:2142–52. 27590221 10.1016/S0140-6736(16)31326-5

[28] Bohm M, Schumacher H, Teo KK, Lonn EM, Mahfoud F, Mann JFE, et al. Achieved blood pressure and cardiovascular outcomes in high-risk patients: results from ONTARGET and TRANSCEND trials. Lancet. 2017;389:2226–37. 28390695 10.1016/S0140-6736(17)30754-7

[29] Rahman F, Mcevoy JW. The J-shaped curve for blood pressure and cardiovascular disease risk: historical context and recent updates. Curr Atheroscler Rep. 2017;19:34. 28612327 10.1007/s11883-017-0670-1

[30] Flint AC, Conell C, Ren X, Banki NM, Chan SL, Rao VA, et al. Effect of systolic and diastolic blood pressure on cardiovascular outcomes. N Engl J Med. 2019;381:243–51. 31314968 10.1056/NEJMoa1803180

[31] Mcevoy JW, Chen Y, Rawlings A, Hoogeveen RC, Ballantyne CM, Blumenthal RS, et al. Diastolic blood pressure, subclinical myocardial damage, and cardiac events: implications for blood pressure control. J Am Coll Cardiol. 2016;68:1713–22. 27590090 10.1016/j.jacc.2016.07.754 PMC5089057

[32] Kannel WB, Wilson PWF, Nam B-H, D’agostino RB, Li J. A likely explanation for the J-curve of blood pressure cardiovascular risk. Am J Cardiol. 2004;94:380–4. 15276113 10.1016/j.amjcard.2004.04.043

[33] Bhatt DL. Troponin and the J-curve of diastolic blood pressure: when lower is not better. J Am Coll Cardiol. 2016;68:1723–6. 27590091 10.1016/j.jacc.2016.08.007

[34] Manning LS, Rothwell PM, Potter JF, Robinson TG. Prognostic significance of short-term blood pressure variability in acute stroke: systematic review. Stroke. 2015;46:2482–90. 26243226 10.1161/STROKEAHA.115.010075

[35] De Havenon A, Fino NF, Johnson B, Wong KH, Majersik JJ, Tirschwell D, et al. Blood pressure variability and cardiovascular outcomes in patients with prior stroke: a secondary analysis of PRoFESS. Stroke. 2019;50:3170–6. 31537194 10.1161/STROKEAHA.119.026293 PMC6817411

[36] Nepal G, Shrestha GS, Shing YK, Muha A, Bhagat R. Systolic blood pressure variability following endovascular thrombectomy and clinical outcome in acute ischemic stroke: A meta-analysis. Acta Neurol Scand. 2021;144:343–54. 34110006 10.1111/ane.13480

[37] Toyoda K, Yamagami H, Kitagawa K, Kitazono T, Nagao T, Minematsu K, et al. Blood pressure level and variability during long-term prasugrel or clopidogrel medication after stroke: PRASTRO-I. Stroke. 2021;52:1234–43. 33563017 10.1161/STROKEAHA.120.032824

[38] Toyoda K. Intensive blood pressure lowering for ischemic stroke patients: does it prevent ischemia or bleeding? Hypertens Res. 2022;45:769–71. 35318451 10.1038/s41440-022-00892-6 PMC9010285

[39] Uchiyama S, Toyoda K, Omae K, Saita R, Kimura K, Hoshino H, et al. Dual antiplatelet therapy using cilostazol in patients with stroke and intracranial arterial stenosis. J Am Heart Assoc. 2021;10:e022575. 34622679 10.1161/JAHA.121.022575 PMC8751870

