# Supplementary material for: Blood pressure during long-term cilostazol-based dual antiplatelet therapy after stroke: a post hoc analysis of the CSPS.com trial
Source: Hypertens Res. 2024 Jul 9;47(9):2238–49. doi: 10.1038/s41440-024-01742-3 (PMC11374707; doi:10.1038/s41440-024-01742-3)
Supplement: Supplementary file 2 — Supplementary information [file 41440_2024_1742_MOESM2_ESM.pdf]

# Protocol

This supplement contains the following items:

1. CSPS.com Study Protocol Original v1.0, Final v1.3, and summary of changes

Protocol v1.0 ………………………………………………………. 1
Protocol v1.3 ……….…………………..…………………………… 42
Summary of Changes ………………………………….…………… 85

2. Original statistical analysis plan (unchanged) ………..………… 89

# Study of Antiplatelet Combination Therapy for Ischemic Stroke Patients with High Risk of Recurrence

# CSPS.com

**(Cilostazol Stroke Prevention Study.** *Combination***)**

## Clinical Study Protocol

Principal investigator: Takenori Yamaguchi, President Emeritus,
National Cerebral and Cardiovascular Center

Protocol Identification Number: 021-TADD-1300-1
Version 1.0: 15 September, 2013

Protocol Summary

| | |
|---|---|
| Study title | Study of antiplatelet combination therapy for ischemic stroke patients with highrisk of recurrence<br>CSPS.com (Cilostazol Stroke Prevention Study. *Combination*) |
| Objectives | To examine the efficacy and safety of dual antiplatelet therapy (DAPT) including cilostazol (Pletaal OD Tablet® & reg.) in comparison with single antiplatelet therapy (SAPT) excluding cilostazol for secondary prevention of ischemic stroke in high-risk patients for stroke |
| Study design | Multicenter, randomized, controlled trial |
| Condition | Noncardioembolic ischemic stroke |
| Inclusion Criteria | Patients must meet all of the following criteria to be eligible for the trial:<br>1) Patients with a diagnosis of noncardioembolic ischemic stroke that developed between 8 and 180 days before the first day of observation<br>2) Patients with a responsible lesion identified by head MRI<br>3) Patients 20 to 85 years of age when providing informed consent<br>4) Patients taking aspirin or clopidogrel alone as antiplatelet therapy when providing informed consent<br>5) Patients meeting at least one of the following criteria:<br>i. At least 50% stenosis of a major intracranial artery<br>ii. At least 50% stenosis of an extracranial artery<br>iii. Two or more of the following risk factors:<br>• 65 years of age or older<br>• Diabetes mellitus<br>• Hypertension<br>• Peripheral arterial disease<br>• Chronic kidney disease<br>• History of symptomatic ischemic stroke (IS) (excluding the index IS for this study)<br>• History of ischemic heart disease<br>• Smoking (only current smokers, excluding previous smokers)<br>6) Patients able to visit the study center throughout the observation period<br>7) Patients who provided written informed consent for participation |
| Exclusion criteria | Patients meeting any of the following criteria will be excluded:<br>1) Patients with emboligenic heart disease<br>2) Patients taking any anticoagulant agents<br>3) Patients who cannot undergo MRI examination for reasons such as |

| | |
|---|---|
| | claustrophobia or implanted pacemaker<br>4) Patients scheduled to undergo any surgery during the study period, including percutaneous angioplasty, stent placement, or bypass grafting<br>5) Patients with a drug-eluting coronary stent implanted within the past year<br>6) Patients with a history of symptomatic non-traumatic intracranial hemorrhage, any other hemorrhagic disease (e.g. active peptic ulcer), bleeding predisposition, or blood clotting disorders<br>7) Patients with a history of hypersensitivity to cilostazol<br>8) Patients with congestive heart failure or uncontrollable angina pectoris<br>9) Patients with thrombocytopenia (platelet count ≤ 100,000/$mm^3$)<br>10) Patients with severe liver or renal dysfunction<br>11) Women who are pregnant, breast-feeding, or of childbearing potential<br>12) Patients with a malignant tumor requiring treatment<br>13) Patients who are taking aspirin, and meet any of the following criteria:<br>• History of hypersensitivity to aspirin or salicylic acid analogues<br>• Peptic ulcer complication<br>• Aspirin-induced asthma or its history<br>14) Patients who take clopidogrel, and meet the following criterion:<br>• History of hypersensitivity to clopidogrel<br>15) Patients who are participating in any other clinical studies<br>16) Patients considered by the investigator/subinvestigator to be unsuitable for participating in this study |
| Endpoints | Primary endpoint<br>• Recurrence of symptomatic ischemic stroke ("ischemic stroke" hereafter)<br><br>Secondary endpoints<br>• Any stroke [ischemic stroke (IS), intracerebral hemorrhage (ICH), or subarachnoid hemorrhage (SAH)]<br>• ICH or SAH<br>• Ischemic cerebrovascular disorder [IS or transient ischemic attack (TIA)]<br>• Death from any cause<br>• Stroke [IS, ICH, SAH], myocardial infarction (MI), or vascular death<br>• All vascular events: stroke, MI, and other vascular events [e.g. aortic dissection or rupture; pulmonary embolism; heart failure, angina pectoris, or occlusive arteriosclerosis requiring hospitalization; and revascularization of coronary artery, aorta, peripheral artery, etc.] |

| | |
|---|---|
| | Safety endpoints<br>• Adverse events and adverse drug reactions<br>• Severe or life-threatening hemorrhage (GUSTO Criteria) |
| Dosage, administration, and treatment period | SAPT group<br>One of the following drugs will be administered:<br>Aspirin: 81 or 100 mg/day (once daily)<br>Clopidogrel: 50 or 75 mg/day (once daily)<br>DAPT group<br>One of the following drugs will be administered:<br>Aspirin: 81 or 100 mg/day (once daily)<br>Clopidogrel: 50 or 75 mg/day (once daily)<br><br>As a rule, cilostazol (Pletaal OD Tablet® & reg.) will be co-administered twice daily at the maintenance dose of 200 mg/day. However, the starting dose can be 100 mg/day, in which case the dose must fundamentally be increased to 200 mg/day within 15 days. |
| Target sample size | 4,000 |
| Study period | October 2013 to March 2017 |

## Study Design

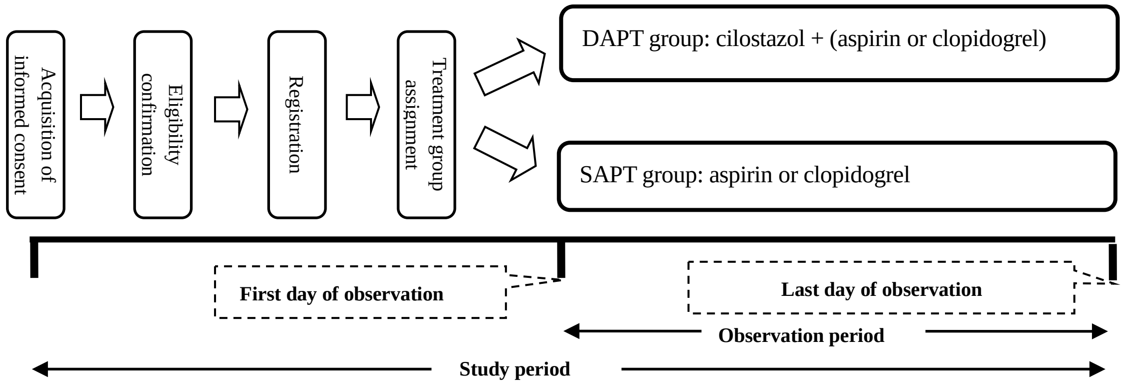


## Observation schedule

| Evaluation items \ Time point | (Date of onset) | Enroll-ment | Start of obser-vation | Month 1 | Month 3 | Month 6 | Month 12 | Every 6 months there-after | End of obser-Vation (*) |
|---|---|---|---|---|---|---|---|---|---|
| Informed consent | ← | ○ | | | | | | | |
| Enrollment | | ○ | | | | | | | |
| Observation status | | | | ○ | ○ | ○ | ○ | ○ | ○ |
| Patient characteristics | ← | ○ | | | | | | | |
| Degree of independence in daily living (mRS) | | | ○ | ○ | ○ | ○ | ○ | ○ | ○ |
| Study drug administration/ compliance | | | ○ | ○ | ○ | ○ | ○ | ○ | ○ |
| Drugs other than study drug | | | ○ | ○ | ○ | ○ | ○ | ○ | ○ |
| Blood pressure | | | ○ | ○ | ○ | ○ | ○ | ○ | ○ |
| Head MRI | ← | ○ | | | | | | | |
| Head MRI (T2* WI) | ← | △ | → | | | | | | |
| Head MRA | ← | △ | → | | | | | | |
| Carotid artery imaging | ← | △ | → | | | | | | |
| Laboratory test (blood) | ← | ○ | → | ←⋯ | ⋯ | ⋯ | ⋯ | ⋯ | ⋯→ |
| Laboratory test (urine) | ← | ○ | → | ←⋯ | ⋯ | ⋯ | ⋯ | ⋯ | ⋯→ |
| Chest x-ray | ← | ○ | → | ←⋯ | ⋯ | ⋯ | ⋯ | ⋯ | ⋯→ |
| ECG | ← | ○ | → | ←⋯ | ⋯ | ⋯ | ⋯ | ⋯ | ⋯→ |
| Adverse events | | | ←— | — | — | — | — | — | —→ |

○ Required item △ Optional item ←⋯→ As required

* Observation is complete when a subject meets criteria for discontinuation/completion, or when 1 year has elapsed from initiation of observation for last subject enrolled.

## Table of Contents

**1. STUDY OBJECTIVES** ...................................................... エラー! ブックマークが定義されていません。

**2. BACKGROUND AND RATIONALE** ............................. エラー! ブックマークが定義されていません。

2.1. PRESENT STATE OF DUAL ANTIPLATELET THERAPY IN PREVENTING ISCHEMIC STROKE RECURRENCE. エラー! ブックマークが定義されていません。

2.2. COMBINED CILOSTAZOL-ASPIRIN THERAPY IN PATIENTS WITH ISCHEMIC STROKE.. エラー! ブックマークが定義されていません。

2.3. DUAL ANTIPLATELET THERAPY USING CILOSTAZOL IN PATIENTS WITH CARDIOVASCULAR DISEASE エラー! ブックマークが定義されていません。

**3. PATIENT SELECTION**..................................................... エラー! ブックマークが定義されていません。

3.1. TARGET DISEASE ..................................................................エラー! ブックマークが定義されていません。

3.2. INCLUSION CRITERIA ...........................................................エラー! ブックマークが定義されていません。

3.3. EXCLUSION CRITERIA ..........................................................エラー! ブックマークが定義されていません。

**4. ENROLLMENT AND ALLOCATION** .......................... エラー! ブックマークが定義されていません。

4.1. ENROLLMENT AND ALLOCATION ........................................エラー! ブックマークが定義されていません。

4.2. ALLOCATION METHODOLOGY .............................................エラー! ブックマークが定義されていません。

**5. TREATMENT PLAN AND CRITERIA FOR CHANGES IN DOSAGE OR SCHEDULE..** エラー! ブックマークが定義されていません。

5.1. PROTOCOL TREATMENT......................................................エラー! ブックマークが定義されていません。

5.2. CRITERIA FOR CHANGES IN DOSAGE OR TREATMENT SCHEDULE.エラー! ブックマークが定義されていません。

5.3. CRITERIA FOR SUBJECT DISCONTINUATION OR COMPLETIONエラー! ブックマークが定義されていません。

5.4. CONCOMITANT THERAPIES .................................................エラー! ブックマークが定義されていません。

**6. STUDY PROCEDURES**.................................................... エラー! ブックマークが定義されていません。

6.1. OBSERVATIONS/TESTS AND OBSERVATION SCHEDULE .......エラー! ブックマークが定義されていません。

6.2. OBSERVATION DAYS ..........................................................エラー! ブックマークが定義されていません。

6.3. OBSERVATION, TEST, AND REPORT ITEMS...........................エラー! ブックマークが定義されていません。

**7. ADVERSE EVENTS**.......................................................... エラー! ブックマークが定義されていません。

7.1. DEFINITION .........................................................................エラー! ブックマークが定義されていません。

7.2. PROCEDURES FOR ADVERSE EVENTS ...................................エラー! ブックマークが定義されていません。

7.3. EVALUATION OF ADVERSE EVENTS.....................................エラー! ブックマークが定義されていません。

7.4. FOLLOW-UP OF ADVERSE EVENTS.......................................エラー! ブックマークが定義されていません。

7.5. EXPECTED ADVERSE DRUG REACTIONS ..............................エラー! ブックマークが定義されていません。
7.6. PROCEDURES FOR SUBJECTS WHO ARE SUSPECTED OR KNOWN TO BE PREGNANT .. エラー! ブックマークが定義されていません。

**8. EFFICACY EVALUATION** ............................................ エラー! ブックマークが定義されていません。

8.1. PRIMARY ENDPOINT ...........................................................エラー! ブックマークが定義されていません。
8.2. SECONDARY ENDPOINTS.....................................................エラー! ブックマークが定義されていません。

**9. SAFETY EVALUATION** .................................................. エラー! ブックマークが定義されていません。

**10. TARGET SAMPLE SIZE AND STUDY PERIOD** ...... エラー! ブックマークが定義されていません。

10.1. TARGET SAMPLE SIZE .........................................................エラー! ブックマークが定義されていません。
10.2. STUDY PERIOD..................................................................エラー! ブックマークが定義されていません。

**11. STATISTICAL ANALYSIS**............................................ エラー! ブックマークが定義されていません。

11.1. RATIONALE FOR TARGET SAMPLE SIZE ..............................エラー! ブックマークが定義されていません。
11.2. ANALYSIS POPULATIONS ...................................................エラー! ブックマークが定義されていません。
11.3. ANALYSIS ITEMS AND METHODS .......................................エラー! ブックマークが定義されていません。
11.4. INTERIM REVIEW ..............................................................エラー! ブックマークが定義されていません。
11.5. FINAL ANALYSIS ................................................................エラー! ブックマークが定義されていません。

**12. CASE REPORT FORM DATA ENTRY AND SUBMISSION** エラー! ブックマークが定義されていません。

**13. DATA MANAGEMENT** .................................................. エラー! ブックマークが定義されていません。

**14. ETHICS**............................................................................ エラー! ブックマークが定義されていません。

14.1. RULES AND REGULATIONS TO BE OBSERVED ....................エラー! ブックマークが定義されていません。
14.2. SUBJECT BENEFITS AND DRAWBACKS ...............................エラー! ブックマークが定義されていません。
14.3. REVIEW AND DISCUSSION BY ETHICS COMMITTEE ............エラー! ブックマークが定義されていません。
14.4. AUTHORING AND REVISION OF INFORMED CONSENT FORMS AND WRITTEN INFORMATION FOR SUBJECTS ... エラー! ブックマークが定義されていません。
14.5. INFORMED CONSENT.........................................................エラー! ブックマークが定義されていません。
14.6. PRIVACY PROTECTIONS AND SUBJECT IDENTIFICATION .....エラー! ブックマークが定義されていません。

**15. STUDY FUNDING AND CONFLICTS OF INTEREST**エラー! ブックマークが定義されていません。

**16. STUDY EXPENSES** ........................................................ エラー! ブックマークが定義されていません。

**17. RESPONSES AND COMPENSATION FOR DAMAGE TO SUBJECT HEALTH** . エラー! ブックマークが定義されていません。

17.1. RESPONSES TO DAMAGE TO HEALTH ................................エラー! ブックマークが定義されていません。
17.2. CLINICAL STUDY INSURANCE ...........................................エラー! ブックマークが定義されていません。

**18. REVISION OF THE STUDY PROTOCOL** ................. エラー! ブックマークが定義されていません。

**19. STUDY COMPLETION AND EARLY DISCONTINUATION** .. エラー! ブックマークが定義されていません。

19.1. STUDY COMPLETION AT EACH STUDY CENTER ..................エラー! ブックマークが定義されていません。
19.2. STUDY CANCELLATION OR DISCONTINUATION AT EACH STUDY CENTERエラー! ブックマークが定義されていません。
19.3. OVERALL COMPLETION OF STUDY ....................................エラー! ブックマークが定義されていません。
19.4. EARLY DISCONTINUATION OF STUDY ................................エラー! ブックマークが定義されていません。

**20. STORAGE OF STUDY-RELATED MATERIALS** ...... エラー! ブックマークが定義されていません。

**21. CLINICAL STUDY REGISTRATION** ......................... エラー! ブックマークが定義されていません。

**22. AUTHORING OF CLINICALSTUDY REPORT, DISCLOSURE OF STUDY RESULTS, AND OWNERSHIP OF RESULTS AND INTELLECTUAL PROPERTY**エラー! ブックマークが定義されていません。

**23. PROCEDURES FOR ADVERSE EVENTS** ................. エラー! ブックマークが定義されていません。

23.1. SUBJECT-RELATED RESPONSE ..........................................エラー! ブックマークが定義されていません。
23.2. SERIOUS ADVERSE EVENT REPORTING ..............................エラー! ブックマークが定義されていません。

**24. EVALUATION OF ADVERSE EVENTS** ...................... エラー! ブックマークが定義されていません。

24.1. EVENT NAME ...................................................................エラー! ブックマークが定義されていません。
24.2. EVENT DATE ....................................................................エラー! ブックマークが定義されていません。
24.3. SEVERITY .........................................................................エラー! ブックマークが定義されていません。
24.4. SERIOUSNESS ...................................................................エラー! ブックマークが定義されていません。
24.5. CAUSAL RELATIONSHIP WITH STUDY DRUGS.....................エラー! ブックマークが定義されていません。
24.6. STUDY DRUG-RELATED ACTIONS ......................................エラー! ブックマークが定義されていません。
24.7. ADVERSE EVENT-RELATED ACTIONS .................................エラー! ブックマークが定義されていません。
24.8. OUTCOME..........................................................................エラー! ブックマークが定義されていません。

**25. FOLLOW-UP INVESTIGATION OF ADVERSE EVENTS**エラー! ブックマークが定義されていません。

**26. DRUG INFORMATION** ................................................ エラー! ブックマークが定義されていません。

26.1. ASPIRIN ............................................................................エラー! ブックマークが定義されていません。
26.2. CLOPIDOGREL ..................................................................エラー! ブックマークが定義されていません。
26.3. CILOSTAZOL .....................................................................エラー! ブックマークが定義されていません。

**27. COMMITTEES** ............................................................... エラー! ブックマークが定義されていません。

27.1. STEERING COMMITTEE .....................................................エラー! ブックマークが定義されていません。
27.2. PROTOCOL AUTHORING COMMITTEE................................エラー! ブックマークが定義されていません。

27.3. INDEPENDENT DATA MONITORING COMMITTEE ...............エラー! ブックマークが定義されていません。
27.4. EVENT EVALUATION COMMITTEE....................................エラー! ブックマークが定義されていません。
27.5. STATISTICAL ANALYSIS COMMITTEE ...............................エラー! ブックマークが定義されていません。

**28. STUDY ORGANIZATION.............................................** エラー! ブックマークが定義されていません。

28.1. STUDY SPONSOR..............................................................エラー! ブックマークが定義されていません。
28.2. PRINCIPAL INVESTIGATOR................................................エラー! ブックマークが定義されていません。
28.3. STEERING COMMITTEE ....................................................エラー! ブックマークが定義されていません。
28.4. PROTOCOL AUTHORING COMMITTEE...............................エラー! ブックマークが定義されていません。
28.5. INDEPENDENT DATA MONITORING COMMITTEE ...............エラー! ブックマークが定義されていません。
28.6. EVENT EVALUATION COMMITTEE....................................エラー! ブックマークが定義されていません。
28.7. STATISTICAL ANALYSIS COMMITTEE ...............................エラー! ブックマークが定義されていません。
28.8. STUDY SECRETARIAT .........................................................エラー! ブックマークが定義されていません。
28.9. DATA CENTER ...................................................................エラー! ブックマークが定義されていません。
28.10. STUDY CENTERS ..............................................................エラー! ブックマークが定義されていません。

**29. REFERENCES.................................................................** エラー! ブックマークが定義されていません。

29.1. LITERATURE QUOTED .......................................................エラー! ブックマークが定義されていません。
29.2. HEART DISEASES WHICH MAY BE SOURCE OF EMBOLI .......エラー! ブックマークが定義されていません。
29.3. DEFINITIONS OF SERIOUS HEPATIC IMPAIRMENT AND SERIOUS RENAL IMPAIRMENT . エラー! ブックマークが定義されていません。
29.4. DEFINITION OF MEDICAL HISTORY AND COMPLICATIONS ..エラー! ブックマークが定義されていません。
29.5. DEGREE OF INDEPENDENCE IN ACTIVITIES OF DAILY LIVINGエラー! ブックマークが定義されていません。
29.6. GUSTO CRITERIA............................................................エラー! ブックマークが定義されていません。

## 1. Study Objectives

To evaluate the efficacy and safety of dual antiplatelet therapy (DAPT) including cilostazol (Pletaal OD Tablet®) in comparison with single antiplatelet therapy (SAPT) excluding cilostazol for secondary prevention of ischemic stroke in high-risk patients.

## 2. Background and Rationale

### 2.1. Present state of dual antiplatelet therapy in preventing ischemic stroke recurrence

Currently, treatment with antiplatelet drugs is strongly recommended for preventing recurrence of noncardioembolic ischemic stroke, along with improvement of the lifestyle and management of risk factors. However, prophylactic efficacy of aspirin for vascular events, for which there is significant evidence, shows a relative risk reduction rate (RRR) of only 20 to 30%. Existing antiplatelet monotherapy does not have sufficient prophylactic efficacy for stroke recurrence, especially in patients with overlapping risk factors such as intracranial artery stenosis, carotid stenosis, and diabetes mellitus/hypertension[1–4]. Therefore, stronger antiplatelet drugs development or dual antiplatelet therapies with different mechanism of action are being examined to enhance prophylactic efficacy for vascular events in patients at high risk for ischemic stroke (IS) or transient ischemic attack (TIA).

With regard to dual antiplatelet therapy, efficacy of combined aspirin and extended-release dipyridamole in IS/TIA patients was confirmed in the ESPS 2 (European Stroke Prevention Study)[5] and ESPRIT (European/Australasian Stroke Prevention in Reversible Ischaemia Trial)[6] studies. However, in Japan, the JASAP study [Japanese Aggrenox (Extended-Release Dipyridamole plus Aspirin) Stroke Prevention versus Aspirin Programme][7] failed to demonstrate the efficacy of this combination, and dipyridamole has not been approved to prevent recurrence of ischemic stroke.

With regard to the combined effect of aspirin and clopidogrel in IS/TIA patients, results of CARESS (Clopidogrel and Aspirin for Reduction of Emboli in Symptomatic Carotid Stenosis)[8], FASTER (Fast Assessment of Stroke and Transient ischaemic attack to prevent Early Recurrence)[9], CLAIR (Clopidogrel plus aspirin versus aspirin alone for reducing embolisation in patients with acute symptomatic cerebral or carotid artery stenosis)[10], etc., demonstrated the efficacy of combined treatment in patients with IS/TIA at relatively early stage of onset. Efficacy of a short-term combination therapy of aspirin and clopidogrel was also demonstrated in the recent CHANE study (Clopidogrel in High-risk patients with Acute Non-disabling Cerebrovascular Events)[11]. However, long-term combined use of aspirin and clopidogrel[12–14] is not recommended as it increases serious bleeding complications, even though there is no change or even a slight decrease observed in vascular events.

### 2.2.Combined cilostazol-aspirin therapy in patients with ischemic stroke

Cilostazol, developed in Japan, significantly inhibited ischemic stroke recurrence and did not increase serious bleeding in a double-blind placebo-controlled study in patients with ischemic stroke (CSPS, Cilostazol Stroke Prevention Study)[15]. Similarly, cilostazol significantly inhibited strokes (ischemic stroke, intracerebral

hemorrhage, and subarachnoid hemorrhage) and reduced serious bleeding to half in a double-blind controlled study with aspirin (CSPS 2, Cilostazol Stroke Prevention Study 2)[16].

The combination therapy of aspirin and cilostazol in patients with ischemic stroke has been tested in three small-scale studies, and no studies demonstrated any significant increase compared with aspirin monotherapy in serious bleeding complications. Summaries of the three studies are presented below.

In the TOSS study (Trial of Cilostazol in Symptomatic Intracranial Arterial Stenosis)[17] examining the inhibitory effects of aspirin or combined aspirin-cilostazol therapy on stenosis progression in patients with intracranial arterial stenosis, combined aspirin-cilostazol therapy significantly inhibited progression of stenosis compared to aspirin monotherapy. There was no increase in intracerebral hemorrhage due to the combination therapy, and results suggested effectiveness and safety of combined aspirin and cilostazol. However, the observation period in this study was only 6 months.

The subsequent TOSS 2 study (Trial of Cilostazol in Symptomatic Intracranial Arterial Stenosis)[18] examined the inhibitory effects of combined aspirin and clopidogrel versus aspirin and cilostazol on stenosis progression in patients with intracranial arterial stenosis. The study failed to demonstrate significant inhibition of stenosis progression, but serious bleeding was less frequent (0.9%) in the aspirin and cilostazol group compared to the aspirin and clopidogrel group (2.6%).

The CATHARSIS study (Cilostazol-Aspirin Therapy Against Recurrent Stroke with Intracranial artery Stenosis)[19] followed stenosis progression and stroke recurrence in an aspirin monotherapy group and an aspirin-cilostazol combination group in patients with intracranial arterial stenosis for 2 years. No significant differences were observed in intracranial artery stenosis progression, perhaps due to a background factor bias. However, strokes were less frequent in the aspirin-cilostazol combination group (2.5%/year) than the aspirin monotherapy group (4.5%/year), and there was no increase in serious bleeding in the combination group compared to the monotherapy group. This set of results has demonstrated that combination therapy using cilostazol is superior in safety compared with conventional dual antiplatelet therapy.

### 2.3. Dual antiplatelet therapy using cilostazol in patients with cardiovascular disease

With regard to cardiovascular diseases other than strokes, an overseas study has reported that the combination of cilostazol and other antiplatelet drugs does not increase bleeding complications.

A crossover study examining the effects of various combination therapies utilizing 3 antiplatelet drugs on bleeding time in patients with peripheral arterial disease[20] showed a significant prolongation of bleeding time with the aspirin-clopidogrel combination compared to monotherapy with each drug. However, no significant prolongation of bleeding time was observed with cilostazol in combination with either aspirin or clopidogrel. Moreover, no prolongation of bleeding time was observed when cilostazol was added to the aspirin-clopidogrel combination therapy.

The CASTLE study (Cilostazol: A Study in Long-term Effects)[21] was conducted with the primary objective of evaluating safety in patients with peripheral arterial disease. The effects of cilostazol and placebo were compared in 1,439 patients for a mean observation period of about 1.5 years. About 70% of patients used aspirin and about 27% used clopidogrel concomitantly in both placebo and cilostazol groups, but no

difference was observed in serious bleeding events between the two groups.

A meta-analysis[22] examined prophylactic effects on stenosis recurrence and safety following drug-eluting stent implantation in patients with coronary artery disease. No significant difference in bleeding events or serious bleeding events was observed between groups receiving either aspirin and clopidogrel or a 3-drug combination with cilostazol added.

These reports suggest that the adding either aspirin or clopidogrel to cilostazol use does not increase risk of bleeding complications.

Based on these clinical results, the current study will examine prophylactic efficacy on ischemic stroke recurrence between SAPT group and DAPT group with cilostazol added to conventional antiplatelet therapy in high risk patients with at least two risk factors of intracranial arterial stenosis, carotid stenosis, or ischemic stroke, with observation period at least one year.

## 3. Patient Selection

### 3.1. Target disease

Noncardioembolic ischemic stroke

### 3.2. Inclusion criteria

Patients meeting all of the following criteria will be included:

1) Patients with a diagnosis of noncardioembolic ischemic stroke that developed between 8 and 180 days before the first day of observation
2) Patients with a responsible lesion identified by head MRI
3) Patients 20 to 85 years of age when providing informed consent
4) Patients taking aspirin or clopidogrel alone as antiplatelet therapy when providing informed consent
5) Patients meeting at least one of the following criteria:
   i. At least 50% stenosis of a major intracranial artery (to the level of A2, M2, P2)
   ii. At least 50% stenosis of an extracranial artery*
   
   * common carotid artery, internal carotid artery, vertebral artery, brachiocephalic artery, or subclavian artery
   iii. Two or more of the following risk factors:
      - 65 years of age or older
      - Diabetes mellitus
      - Hypertension
      - Peripheral arterial disease
      - Chronic kidney disease
      - History of symptomatic ischemic stroke (excluding the index IS for this study)
      - History of ischemic heart disease
      - Smoking (only current smokers, excluding previous smokers)
6) Patients able to visit the study center throughout the observation period

7) Patients who provided written informed consent for participation

*Rationale for inclusion criteria*

1) A minimum of 8 days after onset was set because symptoms should be stable to a certain extent, and a maximum of 180 days after onset was set because recurrence rate decreases gradually over time.
2) To confirm the diagnosis of ischemic stroke
3) A minimum age of 20 years was set because that is required for valid consent, and a maximum age of 85 years was set because of the length of the observation period.
4) Patients taking aspirin or clopidogrel alone when providing informed consent were considered suitable based on the study design.
5) The criteria were established to determine high risk of stroke recurrence.
6) Established in order for the patients to be able to undergo planned observation and tests.
7) Complied with the “Ethical Guideline for Clinical Studies.”

### 3.3. Exclusion Criteria

Patients meeting any of the following criteria will be excluded:

8) Patients with emboligenic heart disease (See *29. References*)
9) Patients taking any anticoagulant agents
10) Patients who cannot undergo MRI examination for reasons such as claustrophobia or implanted pacemaker
11) Patients scheduled to undergo any surgery during the study period, including percutaneous angioplasty, stent placement, or bypass grafting
12) Patients with a drug-eluting coronary stent implanted within the past year
13) Patients with a history of symptomatic non-traumatic intracranial hemorrhage, any other hemorrhagic disease (e.g. active peptic ulcer), bleeding predisposition, or blood clotting disorders
14) Patients with a history of hypersensitivity to cilostazol
15) Patients with congestive heart failure or uncontrolled angina pectoris
16) Patients with thrombocytopenia (platelet count $\leq$ 100,000/mm$^3$)
17) Patients with severe liver or renal dysfunction (See *29. References*)
18) Women who are pregnant, breast-feeding, or of child-bearing potential
19) Patients with malignant tumor requiring treatment
20) Patients who are taking aspirin and meet any of the following criteria:
    - History of hypersensitivity to aspirin or salicylic acid analogues
    - Peptic ulcer complication
    - Aspirin-induced asthma or its history
21) Patients who are taking clopidogrel, and meet the following criterion:
    - History of hypersensitivity to clopidogrel
22) Patients who are participating in any other clinical studies
23) Patients considered by the investigator/subinvestigator to be unsuitable for this study

*Rationale for exclusion criteria*

24) To eliminate risk of enrolling patients with cardioembolic ischemic stroke
25) Anticoagulant agents are specified as prohibited concomitant medications
26) Prevents specified examinations
27) Evaluation will be strongly affected by these surgeries
28) Dual antiplatelet therapy is necessary for one year after the drug-eluting stent implantation
29) to 16) To secure the safety of study subjects based on the precautions for using cilostazol, aspirin, and clopidogrel

# 4. Enrollment and Allocation

## 4.1. Enrollment and allocation

The investigator/subinvestigator must obtain written informed consent from all patients prior to study enrollment. After obtaining written informed consent, the investigator/subinvestigator must assign a uniformly-determined subject identification code (see *14.6 Privacy protections and subject identification*) and enroll the subject through the Internet enrollment system. After enrollment, the investigator/subinvestigator must find out which treatment group the subject was allocated to from the enrollment system. For details, refer to the *Subject enrollment manual*.

The following items must be input into the system when subjects are enrolled:

Subject identification code
Date of written informed consent
Sex
Date of birth
Date of most recent noncardioembolic IS
Eligibility information (confirmation of inclusion and exclusion criteria)
Scheduled date of first visit

## 4.2. Allocation methodology

The enrollment center will allocate subjects to treatment groups using blocked randomization according to study center.

# 5. Treatment plan and criteria for changes in dosage or schedule

## 5.1. Protocol treatment

### 5.1.1. Dosage and administration

SAPT group

One of the following drugs will be administered:

| | |
|---|---|
| Aspirin: | 81 or 100 mg/day (once daily) |
| Clopidogrel: | 50 or 75 mg/day (once daily) |

DAPT group

One of the following drugs will be administered:

| | |
|---|---|
| Aspirin: | 81 or 100 mg/day (once daily) |
| Clopidogrel: | 50 or 75 mg/day (once daily) |

As a rule, cilostazol (Pletaal OD tablet® & reg.) will be co-administered twice daily at the maintenance dose of 200 mg/day. However, the starting dose can be 100 mg/day, in which case the dose must fundamentally be increased to 200 mg/day within 15 days.

Cilostazol, aspirin and clopidogrel are referred to below as study drugs.

#### 5.1.2. Treatment initiation timing

Treatment with cilostazol will be initiated on the first day of observation.

Type or dosage of aspirin or clopidogrel will not be changed after obtaining written informed consent.

#### 5.1.3. Confirming treatment status

The investigator/subinvestigator must confirm subject's treatment status by interview and record it on his/her medical record.

### 5.2. Criteria for changes in dosage or treatment schedule

Dosage or treatment schedule of aspirin or clopidogrel will fundamentally not be changed during the observation period.

If a headache etc. occurs after treatment with cilostazol at a dose of 200 mg/day, the treatment will continue at a reduced dosage of 100 mg/day.

Study drug may be withdrawn in event of surgery or other invasive procedures, but the period of drug withdrawal may not last more than 4 weeks.

### 5.3. Criteria for subject discontinuation or completion

If it is determined that continued observation of a subject is impossible due to the following reasons, that subject will be withdrawn from the study and the reason and date for this recorded. All necessary observations and tests will be performed at this time, and efficacy and safety evaluations performed.

30) Subject requests discontinuation or withdraws consent
31) A serious adverse event occurs which makes continuation problematic
32) Any event described in 6.3.3. occurs
33) Drug withdrawal lasts longer than 4 weeks
34) Any other reasons which lead the investigator/subinvestigator to consider continued participation problematic

Observation will also be discontinued for patients who discontinued taking study drugs.

## 5.4. Concomitant therapies

Cardiovascular risk factors, including hypertension, dyslipidemia, and diabetes mellitus will be controlled according to generally recommended guidelines using any type of drug. There are no restrictions for physical therapy or diet therapy.

Blood pressure should preferably be controlled with a target systolic blood pressure of ≤130 mmHg in order to inhibit intracranial hemorrhage.[23, 24]

Prohibited concomitant medications

Concomitant use of antiplatelet and anticoagulant drugs other than the study drugs is prohibited.

Antiplatelet drugs

Sarpogrelate hydrochloride, dipyridamole, beraprost sodium, limaprost alfadex, alprostadil, ozagrel sodium, and ticlopidine

Anticoagulants

Warfarin potassium, dabigatran, edoxaban, rivaroxaban, apixaban, heparin products (except for topical external preparation), low-molecular-weight heparin, danaparoid sodium, argatroban, urinastatin, gabexate mesilate, nafamostat mesilate, antithrombin III (freeze-dried concentrated), and human activated protein C (freeze-dried concentrated)

Concomitant medications requiring caution

Caution must be exercised when the following drugs are used concomitantly. For details, refer to the insert information of each product.

A) Aspirin group

Gout drugs, diabetes mellitus drugs, methotrexate, lithium products, thiazide diuretics, furosemide, azosemide, piretanide, nonsteroidal anti-inflammatory analgesics, ibuprofen, oxicam anti-inflammatory analgesics, alminoprofen, nitroglycerin products, tetracycline antibiotics, new quinolone antibacterials, adrenocortical hormone drugs, sodium lactate, sodium valproate, phenytoin, acetazolamide, angiotensin converting enzyme inhibitors, and β-blockers

B) Clopidogrel group

Nonsteroidal anti-inflammatory analgesics and thrombolytic drugs

Drug-metabolizing enzyme (CYP2C19) inhibitors: omeprazole etc.

C) DAPT group only

Drug-metabolizing enzyme (CYP3A4) inhibitors: macrolide antibiotics, HIV protease inhibitors,

azole antifungals, cimetidine, diltiazem, grapefruit juice, etc.

Drug-metabolizing enzyme (CYP2C19) inhibitors: omeprazole etc.

# 6. Study Procedures

## 6.1. Observations/tests and observation schedule

Subject observation will follow the schedule below. Timing of each observation is based on the first visit.

| Evaluation item \ Time point | (Date of onset) | Enroll-ment | Start of obser-vation | Month 1 | Month 3 | Month 6 | Month 12 | Every 6 months there-after | End of obser-Vation (*) |
|---|---|---|---|---|---|---|---|---|---|
| Informed consent | ← | ○ | | | | | | | |
| Enrollment | | ○ | | | | | | | |
| Observation status | | | | ○ | ○ | ○ | ○ | ○ | ○ |
| Patient characteristics | ← | ○ | | | | | | | |
| Degree of independence in daily living (mRS) | | | ○ | ○ | ○ | ○ | ○ | ○ | ○ |
| Study drug administration/ compliance | | | ○ | ○ | ○ | ○ | ○ | ○ | ○ |
| Drugs other than study drug | | | ○ | ○ | ○ | ○ | ○ | ○ | ○ |
| Blood pressure | | | ○ | ○ | ○ | ○ | ○ | ○ | ○ |
| Head MRI | ← | ○ | | | | | | | |
| Head MRI (T2* WI) | ← | △ | → | | | | | | |
| Head MRA | ← | △ | → | | | | | | |
| Carotid artery imaging | ← | △ | → | | | | | | |
| Laboratory test (blood) | ← | ○ | → | ←····· | ····· | ····· | ····· | ····· | ·····→ |
| Laboratory test (urine) | ← | ○ | → | ←····· | ····· | ····· | ····· | ····· | ·····→ |
| Chest x-ray | ← | ○ | → | ←····· | ····· | ····· | ····· | ····· | ·····→ |
| ECG | ← | ○ | → | ←····· | ····· | ····· | ····· | ····· | ·····→ |
| Adverse events | | | ←— | ——— | ——— | ——— | ——— | ——— | ——→ |

○ Required item △ Optional item ←·····→ As required

* Observation is complete when a subject meets criteria for discontinuation/completion, or when 1 year has elapsed from initiation of observation for last subject enrolled.

## 6.2. Observation days

### 6.2.1. First day of observation

The first day of observation is the first visit made by an enrolled subject after allocation, and shall take place within 30 days of the enrollment date.

The first day of observation for subjects in the SAPT group is the day when tests etc. described in *6.3.1. First day of observation* are complete.

### 6.2.2. At 1, 3, 6, and 12 months

Subjects will be observed at 1, 3, 6, and 12 months (determined days) following the first day of observation.

### 6.2.3. From 12 months onward

Subjects will be observed every 6 months.

### 6.2.4. Acceptable variation of days of observation

Acceptable variations in days of observation are as follows.

| | |
|---|---|
| Month 1 | ± 2 weeks from determined day |
| Month 3 | ± 4 weeks from determined day |
| Month 6 | ± 8 weeks from determined day |
| Month 12 and every 6 months thereafter | ± 8 weeks from determined day |

### 6.2.5. Completion of observation

Observation will be completed for all subjects at 1 year after observation begins or the last subject enrolled in the study.

## 6.3. Observation, test, and report items

### 6.3.1. First day of observation

The following items, including patient background, will be observed. All test data from day of onset to first day of observation will be used.

- Date of first visit
- Age and sex
- Height (cm)
- Weight (kg)
- Date of most recent noncardioembolic ischemic stroke
- Medical history and complications

    Coronary artery disease: No or Yes
    Peripheral arterial disease: No or Yes
    Hypertension: No or Yes
    Diabetes mellitus: No or Yes
    Dyslipidemia: No or Yes
    Chronic kidney disease: No or Yes
    History of ischemic stroke (excluding the index ischemic stroke for this study): No or Yes

- Smoking habit: No (nonsmokers or previous smokers) or Yes (current smokers of ≥1 cigarette/day)
- Modified Rankin Scale (mRS) (see *29.5 Modified Rankin Scale*)
- Statues of study drug treatment

    Drug name, prescription start date, and dosage and administration

- Drugs used other than study drugs
- Blood pressure
- Head MRI (required)

Date of scan (between stroke and enrollment)
Clinical subtype: lacunar, atherothrombotic, or other
Responsible lesion: supratentorial, infratentorial, or both

- Head MRI T2* (optional)
  Date of scan (between stroke and enrollment)
  Microbleeds: No or Yes
- Head MRA (optional)
  Date of scan (between stroke and enrollment)
  At least 50% stenosis of a major intracranial artery (to the level of A2, M2, or P2): No or Yes
- Image evaluation of cervical arteries (optional)
  Laboratory procedures: CTA, MRA, ultrasound, DSA
  Date of scan (between stroke and first observation)
  At least 50% stenosis of an extracranial artery (the common carotid artery, internal carotid artery, vertebral artery, brachiocephalic artery, or subclavian artery): No or Yes
- Clinical laboratory tests
  Hematology: white blood cell count, red blood cell count, hemoglobin, hematocrit, and platelet count
  Clinical chemistry: AST, ALT, TC, TG, HDL-C, creatinine, fasting blood glucose, uric acid, and HbA1c (only for patients with diabetes mellitus)
  Urinalysis: Glucose, protein, and occult blood
- Chest X-rays Normal/Abnormal
- ECG Normal/Abnormal

### 6.3.2. All observations after first month

The following items will be observed or evaluated according to the observation schedule:

- Observation status: Ongoing or unavailable
- Date of observation (date of last information if unavailable for observation)
- Presence or absence of events
  If events have occurred, refer to *6.3.3. Events.*
- Presence or absence of adverse events or serious adverse events
  If events have occurred, refer to *23. Procedures for Adverse Events* and *24. Evaluation of Adverse Events*.
- Modified Rankin Scale (mRS)
- Status of study drug administration
  Administration rate: ≥70% or <70%
  Withdrawal (<4 weeks of continuous drug withdrawal): No or Yes (length)

Discontinuation (≥4 weeks of continuous discontinuation): No or Yes (date and reasons)

- Drugs used other than study drugs
- Blood pressure

#### 6.3.3. Events

If any event described below occurs, the information below must be reported.

Refer to *23. Procedures for Adverse Events* and *24. Evaluation of Adverse Events* for information that must be reported in case of serious adverse events.

If a subject is transported to another medical institution due to an event, the following information will be obtained through cooperation from the subject or family, etc., then entered into medical records and used as reference materials.

Death
 Date, cause, and rationale for cause
Ischemic stroke (IS)
 Date
 Clinical subtype: lacunar, atherothrombotic, or other
 Responsible lesion: supratentorial, infratentorial, or both
Intracerebral hemorrhage (ICH)
 Date
Subarachnoid hemorrhage (SAH)
 Date
TIA
 Date
Myocardial infarction (MI)
 Date
Other vascular events*
 Date and details

* e.g. aortic dissection or rupture; pulmonary embolism; heart failure, angina pectoris, or occlusive arteriosclerosis requiring hospitalization; and revascularization of coronary artery, aorta, peripheral artery, etc.

# 7. Adverse Events

## 7.1. Definition

### 7.1.1. Adverse events

Adverse events are any medically undesirable event observed in a subject who has received a study drug, even if the causal relationship is not known.

In other words, adverse events are any undesirable or unintended sign (including abnormal laboratory values),

symptom, or illness observed under treatment with a study drug, regardless of causal relationship with the treatment.

Aggravation of any symptom, sign, or disease preexisting granting of consent or start of observation, will be handled as a new adverse event.

#### 7.1.2. Serious adverse events

Serious adverse events are adverse events for which any of the following apply:

35) Fatal
36) Life-threatening

    The term "life-threatening" refers to an event in which the subject was at risk of death at the time of the adverse event; it does not refer to an event which hypothetically might have caused death if it were more severe.

37) Requires hospitalization or prolongation of existing hospitalization for treatment
38) Results in persistent or significant disability/incapacity
39) Is a congenital anomaly/birth defect
40) Other conditions, i.e., a medically important event for which treatment is required to avoid risks or items 1)–5) above, even if the event is not immediately life-threatening, or resulting in death or hospitalization. Examples include bronchospasm requiring intensive care in emergency rooms etc.; blood disorder or convulsions not to leading to hospitalization; and drug dependence or abuse.

Explanation of "hospitalization for treatment" for serious adverse events

"Hospitalization for treatment" is defined as hospitalization of a subject in a medical institution (usually for at least one night) for treatment of adverse events, even if no particular treatment is performed (resting cure). This does not include hospitalization for examination or treatment of the primary disease or complications not aggravated from pre-observation conditions; hospitalization for convenience or personal reasons not intended to treat adverse events; and hospitalization for treatment or examination that was already scheduled prior to the first visit.

### 7.2. Procedures for adverse events

Refer to *23. Procedures for Adverse Events.*

### 7.3. Evaluation of adverse events

Refer to *24. Evaluation of Adverse Events.*

### 7.4. Follow-up of adverse events

Refer to *25. Follow-Up Investigation of Adverse Events*

### 7.5. Expected adverse drug reactions

Refer to the package insert of study drugs for expected adverse drug reactions and precautions.

### 7.6. Procedures for subjects who are suspected or known to be pregnant

Patients of childbearing potential are excluded from the study. However, an investigator/subinvestigator will conduct pregnancy tests during the observation period for subjects suspected to be pregnant. If the result is positive, the subject must discontinue study participation. Otsuka Pharmaceutical co., Ltd. must be informed of the matter if the pregnant subject was in the DAPT group. If the subject withdraws from the study, tests performed at discontinuation will be performed to the extent that the pregnancy is not adversely affected. Furthermore, follow-up investigation of the subject will continue until birth or termination of the pregnancy. For subjects in the DAPT group, follow-up results must be reported to Otsuka Pharmaceutical co., Ltd..

## 8. Efficacy Evaluation

### 8.1. Primary endpoint

The following is the study's primary endpoint:

- Recurrence of symptomatic ischemic stroke

### 8.2. Secondary endpoints

The following items are secondary endpoints:

- Any stroke [ischemic stroke (IS), intracerebral hemorrhage (ICH), or subarachnoid hemorrhage (SAH)]
- ICH or SAH
- Ischemic cerebrovascular disorder [IS or transient ischemic attack (TIA)]
- Death from any cause
- Stroke [IS, ICH, SAH], myocardial infarction (MI), or vascular death
- All vascular events: stroke, MI, and other vascular events [e.g. aortic dissection or rupture; pulmonary embolism; heart failure, angina pectoris, or occlusive arteriosclerosis requiring hospitalization; and revascularization of coronary artery, aorta, peripheral artery, etc.]

## 9. Safety Evaluation

The following are safety endpoints:

- Adverse events and adverse drug reactions
- Severe or life-threatening hemorrhage (GUSTO criteria)

## 10. Target Sample Size and Study Period

### 10.1. Target sample size

4,000 (2,000 per group)

### 10.2. Study period

The subject enrollment period lasts until target sample size is met. This period is planned to last from October 2013 to March 2016.

Study period lasts until 1 year after the first observation day of the last subject enrolled. This period is planned to last from October 2013 to March 2017.

## 11. Statistical Analysis

### 11.1. Rationale for target sample size

We assumed an annual recurrence rate of 4% in the monotherapy group based on data from the EVEREST study[25] and the aspirin monotherapy group in the JASAP study[7]. We also assumed a 30% decrease in relative recurrence risk (in other words, an annual relapse rate of 2.8%) in the DAPT group with cilostazol. The number of subjects required was calculated at 1,688 per group using the methods described by Lakatos et al[26] in our main analytical method of the log-rank test (conditions were assumed relapse rates for each group, a 2.5 year enrollment period, a maximum 3.5 year observation period, $\alpha$=0.05, and a statistical power of 80%). This results in a total target sample size of 4,000 (2,000 per group), assuming an annual dropout rate of 5% during the study period.

### 11.2. Analysis populations

The intent-to-treat (ITT) analysis population used for evaluating safety and efficacy is defined as all randomly allocated study subjects. In compliance with the CONSORT Statement, we will create a flow diagram containing numbers of enrolled subjects, randomly allocated subjects, subjects observed for 1 year, subjects observed for 2 years, etc.

### 11.3. Analysis items and methods

Demographic characteristics of patient backgrounds will be calculated for each group in the ITT analysis population. Continuous variables are shown as mean (standard deviation) or median (quartile ranges), while category variables are shown as number of subjects (percentage) per category. Mean and median values will also be calculated for length of subject observation periods. Intergroup bias will also be studied with appropriate tests based on variable characteristics.

Kaplan-Meier plots will be drawn by group for the primary endpoint of ischemic stroke recurrence, while groups will be compared with the log-rank test. Also, the Cox proportional hazards model will be used to calculate hazard ratios and 95% CIs for DAPT groups versus SAPT groups. Unadjusted hazard ratios and adjusted hazard ratios [adjusted according to age, sex, clinical type of cerebral infarction (atheroma or lacunar), and mRS] will be calculated at this time.

Annual recurrence rates will be calculated with both person-year and Kaplan-Meier methods, 95% CIs for which will be calculated with the approximate Poisson and Greenwood methods.

Subgroup analysis will include the following factors: age, sex, antiplatelet therapy (aspirin or clopidogrel), clinical subtype of ischemic stroke (atheroma or lacunar), presence or absence of 50% or greater stenosis of

extracranial arteries, mRS, medical history and complications (hypertension, diabetes mellitus, dyslipidemia, coronary heart disease, peripheral artery disease, chronic kidney disease, and ischemic stroke), smoking status, and BMI. Continuous variables will be divided at the median, at which point a Cox proportional hazards model including a comparison group, predictive factors, and interactions will be studied. Stratified hazard ratios of the combination group compared to the SAPT group will be calculated according to predictive factors. Uniformity of interactions will also be studied. Significance level for all statistical tests is bilateral 5%.

Details of statistical analysis are described in the statistical analysis protocol authored by the Statistical Analysis Committee and the data center.

## 11.4. Interim review

### 11.4.1. Interim review methods

Because this study involves a long observation period, interim review will be performed in order to secure subject safety. An interim review of safety endpoints (serious adverse events and hemorrhagic events), in principal, will be performed once per year. Besides the above reviews of safety endpoints, a review of efficacy and safety endpoints will also be performed once only during the study.

The interim review of safety and efficacy endpoints will be performed when 50% of information has been obtained (when 4,000 person-years of data have been collected, or specifically approximately when the 2.5 year enrollment period ends). The Haybittle-Peto ad-hoc method[27, 28] will be used for discontinuation criteria of primary endpoint. No specific discontinuation criteria will be established for safety endpoints, but the Independent Data Monitoring Committee (IDMC) will consider discontinuation or continuation based on incidence rates and make a recommendation to study investigators.

Timing of the interim review of safety and efficacy endpoints was determined based on planned enrollment period, observation period, and withdrawals/dropouts. Because the enrollment period for the study is 2.5 years and observation will end 1 year following the date of initiation of observation of the final subject to enroll, the observation period will last 3.5 years for the first subject to enroll and 1 year for the last subject to enroll. Assuming that subjects will enroll at a regular rate, mean observation period will last 2.25 years for 9,000 total person-years (4,000 × 2.25). However, given the possibility of withdrawals or dropouts, mean observation period is assumed to be 2 years, resulting in a likely total of 8,000 person-years (4,000 subjects × 2 years). Therefore, the review of safety and efficacy endpoints will occur at the mid-point up to 8,000 person-years, or when 4,000 person-years of data has been collected.

### 11.4.2. Interim analysis

As analysis, listing and tabulating safety endpoints (serious adverse events and hemorrhagic events) by treatment will be performed for every safety interim review. As an interim analysis for the review of efficacy and safety endpoints, data fixing will be performed by the Data Center and statistical analysis processing will be performed by IDMC after listing and tabulating endpoints by treatment. Details will be described in the interim analysis protocol, which will be drafted together by the Statistical Analysis Committee, the

Independent Data Monitoring Committee, and the data center by the time of data lock for the first interim analysis.

### 11.5. Final analysis

Final analysis will be performed by the data center after full data lock in accordance with the statistical analysis protocol. Results of this analysis will be compiled in the final analytical report and submitted to the principal investigator and each committee member. The statistical analysis protocol will be authored together by the Statistical Analysis Committee and the data center prior to full data lock.

## 12. Case Report Form Data Entry and Submission

Investigators and subinvestigators will fill out case report form (CRFs) after observing subjects based on the observation schedule. The current study uses an Electronic Data Capture (EDC) system through a special Internet website for CRFs.

Details are described in the *CRF Manual*.

## 13. Data Management

Data collected through the EDC system will be stored and managed at the study's data center. Management methods will adhere to the system's operational procedures.

## 14. Ethics

### 14.1. Rules and regulations to be observed

All staff involved with the study will adhere to the World Medical Association's *Declaration of Helsinki* (amended 10/2008) and the *Ethical Guidelines for Clinical Studies* (fully revised 07/31/2008), as well as the Pharmaceutical Affairs Law and other relevant laws and regulations. All staff will also observe this study protocol.

### 14.2. Subject benefits and drawbacks

The current study falls within the scope of treatment norms, including tests.

Using cilostazol in combination with antiplatelet agents, which are considered to offer insufficient prophylactic efficacy against ischemic stroke recurrence as monotherapy, may mitigate recurrence risk over the long term. This constitutes a benefit for subjects, who are able to receive a new method of treatment that had not been previously available. Observations of subjects scheduled at regular intervals during the study period also means that patients will be receiving regular treatment and diagnostic attention.

As described above in *2. Background and Rationale*, concomitant use of aspirin and cilostazol has not been associated with an increase in hemorrhagic complications. Similarly, concomitant use of the clopidogrel and cilostazol did not result in increased hemorrhagic complications in overseas research on cardiovascular diseases. The same safety is expected with regard to ischemic strokes, but there are no detailed reports available at this time.

**14.3.** Review and discussion by ethics committee

According to *Ethical Guidelines for Clinical Studies*, study center directors must request the institution's ethics committee, either established by the director or otherwise, to make a preliminary review of study protocols to ascertain whether or not they adhere to the guidelines, as well as other issues related to appropriate performance of clinical studies. Items must be brought before the committee as necessary.

**14.4. Authoring and revision of informed consent forms and written information for subjects**

Investigators must author and revise as appropriate informational documents as well as consent forms in order to obtain consent from potential subjects. Authoring and revision of these documents must comply with the ethical principles of both the *Declaration of Helsinki* and the *Ethical Guidelines for Clinical Studies*.

In accordance with the *Declaration of Helsinki* and the *Ethical Guidelines for Clinical Studies*, informed consent forms and written information for subjects must contain the following items:

1) Explanation of the clinical study
2) Target diseases and general treatments
3) Study objectives
4) Explanation of the study drugs
5) Study methodologies
6) Expected advantages and disadvantages for subjects
7) Treatment methods if not participating in study
8) Precautions for study participation
9) Planned study period and number of subjects
10) Study participation and withdrawal
11) Study discontinuation and responses
12) Offering of information related to study
13) Protection of personal information
14) Storage, usage methods, and storage period of materials
15) Response and compensation if subject health is damaged
16) Conflicts of interest and expenses shouldered by subjects
17) Study personnel contacts, study doctors, and contact information

**14.5. Informed consent**

Prior to any subject participation in study, an investigator/subinvestigator will make a full explanation with informational materials and obtain freely-given consent in writing from the subject himself or herself. Once the potential subject fully understands the informational materials, both the investigator/subinvestigator who performed the explanation as well as the patient will sign (or print and affix personal seal) and date the consent documents. Next, the investigator/subinvestigator will give a copy of the signed (or printed with seal affixed) consent documents and informational materials to the subject, and store the original document at the

study center.

Patients may not participate in this study if it is determined they lack the capacity to make their own judgments. However, if for some reason the patient is incapable of signing forms themselves, an alternate method of identification such as a fingerprint may be used to allow participation.

If significant changes are made to the informational materials, the investigator/subinvestigator will explain their content again to subjects using the updated documents, and obtain freely-given consent in writing for continued participation in the study.

### 14.6. Privacy protections and subject identification

All study personnel will strictly protect subject personal information in accordance with the Act on the Protection of Personal Information.

Investigators/subinvestigators will apply a uniformly-determined subject identification code when enrolling patients or when creating CRFs through the EDC system. No personal information which could identify the subject (name, initials, address, telephone number, etc.) will be used. Investigators/subinvestigators will create a chart linking subject identification codes to chart numbers and other personal information of subjects (a subject identification code list), and store this at the study center. Subject identification codes will be of the following format: **study center code (3-digit)—sequential number (3-digit)**. Study center codes will be allocated to each study center by the data center. Subject identification codes will be used to identify subjects when the data center makes inquires to study centers about data.

## 15. Study Funding and Conflicts of Interest

This study is performed under contract between the Japan Cardiovascular Research Foundation and Otsuka Pharmaceutical co., Ltd.. In accordance with rules regarding funding transparency and conflicts of interest of related study institutions, academic societies where results may be presented, publishers, and other entities, the Japan Cardiovascular Research Foundation and Otsuka Pharmaceutical co., Ltd. publically disclose the fact that Otsuka Pharmaceutical co., Ltd. is providing funding for this study.

## 16. Study Expenses

All treatment performed within the scope of the current study is covered by the national health insurance system. No drugs or tests will be used which are not covered by national health insurance. Further, the number of hospital visits and frequency of tests are similar to those under normal treatment, so participating in the current study constitutes no financial burden for subjects.

## 17. Responses and Compensation for Damage to Subject Health

### 17.1. Responses to damage to health

The tests and treatments in the study protocol all lie within the scope of normal treatment. Therefore, in the event of any damage to subject health, investigators/subinvestigators will take all appropriate actions, including full treatment, the expenses for which will be fundamentally compensated by insurance as normal

medical procedures. In the event of serious damage to health, subjects will receive an explanation of procedures for applying for compensation from The Relief System for Sufferers from Adverse Drug Reactions.

### 17.2. Clinical study insurance

Otsuka Pharmaceutical co., Ltd. will enroll in clinical study insurance for the entire study organization in order to cover indemnity liability as well as compensatory liability.

## 18. Revision of the Study Protocol

If revision of the study protocol becomes necessary after initiation of the study, the principal investigator will perform revisions after gaining approval from the Protocol Authoring Committee. If revisions are deemed significant, they must first be reviewed by the ethics committee of the principal investigator's institution.

After revision, the principal investigator will send the revised protocol or details of the revisions to investigators, all committee members, and the data center. Procedures for review of changes at each study center will follow regulations of applicable ethics committees. Finally, investigators at each study center will revise informed consent forms and written information for subjects according to the changes in the study protocol.

## 19. Study Completion and Early Discontinuation

### 19.1. Study completion at each study center

When the study concludes at a study center, the investigator will submit a report of study completion to the center director without delay. The investigator will also submit a copy of the report of study completion to the principal investigator.

### 19.2. Study cancellation or discontinuation at each study center

41) Investigators will assess the issue of whether or not to continue the study in any of the following circumstances:
    i. Significant information is obtained about study drug quality, safety, or efficacy
    ii. A determination has been made that study continuation is problematic
42) Investigators will cancel or discontinue the study in any of the following circumstances:
    i The ethics committee issues an order or recommendation to cancel or discontinue the study
    ii The ethics committee issues problematic orders for changing the study protocol etc.
43) Investigators will inform the principal investigator of any decision made to cancel or discontinue the study as soon as possible in writing, along with reasons for this decision.
44) Investigators will inform subjects of any decision made to cancel or discontinue the study, and arrange for appropriate treatment and post-processing.

### 19.3. Overall completion of study

Study is complete once all CRFs from all study centers are confirmed. Once the principal investigator

receives notification from the data center that all CRFs have been confirmed, he or she will inform investigators and all committee members at each study center that the study has been concluded. Investigators will then notify the director of their institutions and related departments of this in writing.

### 19.4. Early discontinuation of study

The principal investigator will act accordingly if he or she receives a recommendation from the Independent Data Monitoring Committee to discontinue the study early. If the principal investigator determines not to follow this recommendation, he or she will inform the Independent Data Monitoring Committee of this fact together with the basis for the decision, and discuss the issue.

If the principal investigator determines to follow the recommendation to discontinue the study early, he or she will immediately contact all investigators, committee members, and the data center to inform them of the early discontinuation, reasons for it, and follow-up responses. After being informed of the early study discontinuation, investigators will convey this to subjects along with the reasons for it, and perform appropriate alternate treatments. Finally, investigators will then notify the director of their institutions and related departments of the early discontinuation in writing.

## 20. Storage of Study-Related Materials

Investigators will store all documents related to the current clinical study (copies of applications and reports, notifications from study center directors, subject identification code lists, consent documents, CRF copies, and other documents or records necessary for ensuring reliability of data) either until discontinuation or the date 5 years following completion, then dispose of the records in a manner that does not compromise personal information.

## 21. Clinical Study Registration

This clinical study will be registered with UMIN-CTR and ClinicalTrials.gov prior to initiation.

## 22. Authoring of Clinical Study Report, Disclosure of Study Results, and Ownership of Results and Intellectual Property

The principal investigator and the Steering Committee will summarize the contents of the final analysis report and author a clinical study report encompassing overall conclusions from the study, problematic issues, analysis and discussion of results, and plans going forward from a primarily clinical perspective.

Study results will also be reported as a paper, with authors to be determined together by the principal investigator and the Steering Committee.

All data and intellectual property (patent rights, utility model rights, and inventions or devices utilizing these rights) generated by the study will be the property of Otsuka Pharmaceutical co., Ltd..

# 23. Procedures for Adverse Events

## 23.1. Subject-related response

The investigator/subinvestigator give sufficient medical response to all clinically problematic adverse events related to the study both during and after the subject's study participation. The subject should be informed when treatment for adverse events is required.

## 23.2. Serious adverse event reporting

### 23.2.1. Serious adverse events (SAE) requiring immediate reporting

The following SAEs require immediate reporting:

1) All SAEs occurring during the observation period, regardless of their relationship with the study drug.
2) SAEs for which causal relationship with study drugs cannot be denied occurring during the course of a follow-up investigation of an adverse event (see *25. Follow-Up Investigation of Adverse Events*), or an SAE which becomes serious during a follow-up investigation and for which causal relationship with study drugs cannot be denied.
3) SAEs occurring after completion of the study period reported to the investigator/subinvestigator by the subject, and for which the causal relationship to the study drugs cannot be denied according to the investigator/subinvestigator.

### 23.2.2. Immediate reporting procedures

Reporting will adhere to the following procedures:

1) When adverse events corresponding to the previous section occur, the investigator/subinvestigator will immediately input required information into the EDC (CRF) system, which will report relevant information to the study sponsor and Otsuka Pharmaceutical co., Ltd..
2) The investigator/subinvestigator will then submit a detailed report about the SAE (onset of which occurred following study drug treatment) to the study center director as soon as possible, using forms required by the institution. The same information will be input into the EDC (CRF) system, which will report relevant information to the study sponsor and Otsuka Pharmaceutical co., Ltd..
3) The investigator/subinvestigator will furnish additional information about the reported SAE (including autopsy reports, terminal stage medical records, or any other information) to the study sponsor, study center director, ethics committee, and Otsuka Pharmaceutical co., Ltd. as requested.

### 23.2.3. Reporting of additional information following immediate reporting

When new information (e.g. course, test results, procedures, etc.) is obtained about an SAE for which an immediate report has already been made, this information will be input into the EDC system (CRF).

### 23.2.4. Handling of unexpected SAEs

Unexpected SAEs are events which are judged to be adverse drug reaction (ADRs) due to the study drug, but which are not described in the package insert information for that drug; or, if they are described in the

package insert, they differ in quality or severity from that description. These events are handled as follows:

1) **If due to cilostazol**

Otsuka Pharmaceutical co., Ltd. will report unexpected SAEs (ADRs) reported according to *23.2.2. Immediate reporting procedures* to the Minister of Health, Labor and Welfare or a party under contract to said Minister.

2) **If due to other drugs**

The director of each study center will report to the Minister of Health, Labor and Welfare or a party under contract to said Minister in accordance with Article 77-4-2 of Pharmaceutical Affairs Law and *Ethical Guidelines for Clinical Studies.*

#### 23.2.5. SAE reporting to other collaborating clinical study institutions for the study

Otsuka Pharmaceutical co., Ltd. will make lists and tabulate SAEs reported according to *23.2.2. Immediate reporting procedures*, then report to IDMC members every month instead of SAEs reporting to investigators at other site as prescribed by the *Ethical Guidelines for Clinical Studies*, the analysis officer will report the listed SAEs to all investigators.

These reports will not include information on treatment group in order to avoid various biases.

## 24. Evaluation of Adverse Events

The investigator/subinvestigator will evaluate adverse events in accordance with the following:

### 24.1. Event name

Name of disease or condition diagnosed will be used for each event if known instead of individual symptoms.

### 24.2. Event date

Event date is the day of occurrence or confirmation of the adverse event. If diseases, symptoms, or signs existing as when consent was granted become aggravated during the observation period, the day of aggravation is considered the day of adverse event occurrence.

### 24.3. Severity

Severity of adverse events is classified into 3 levels as follows:

1. Mild: Causing discomfort but no interference with daily activities
2. Moderate: Causing discomfort sufficient to restrict or affect daily activities
3. Severe: Cannot perform work or daily activities

### 24.4. Seriousness

Seriousness of adverse events is classified into 2 levels as follows in accordance with *7.1.2. Serious adverse events*.

1. Serious

2. Not serious

### 24.5. Causal relationship with study drugs

Causal relationship with study drugs is classified according to the following 2 categories:

1. Relationship cannot be denied
4. Relationship can be denied

If an investigational drug or concomitant drug is judged responsible, the responsible drug should be identified.

[Reference for determining causal relationship with study drugs]

1. Relationship cannot be denied

   Cases where any of the following reasons make a causal relationship a logical possibility:

   a If it the event is suggested by package insert

   b If there is a suspected temporal relationship between study drug administration and event occurrence (e.g., development of allergic dermatitis several days after study drug administration)

   c If there is a suspected relationship between study drug discontinuation or dosage reduction and event outcome (e.g., rapid recovery of nausea following study drug discontinuation)

   d Other cases in which study drug involvement cannot be medically ruled out

4. Relationship can be denied

   Cases where any of the following reasons make a causal relationship logically improbable:

   a If the event may be due to the primary disease, complications, or medical history

   b If the event may be due to age, sex, or other factors

   c If there is little chance of temporal relationship between study drug administration and event (e.g., adverse events occurring a significant period of time after completion of study drug administration)

   d If there is little chance of relationship due to observed course of event [e.g., subject recovers from event while continuing study drug administration and with no other actions taken (although possibility of habituation to study drug must be taken into account)]

   e If effects of concomitant drug are considered likely

   f If the event is considered likely to be due to chance alone (accident, spontaneous symptoms, etc.) (e.g., femur fracture due to being caught in a traffic accident)

   g Other cases in which study drug involvement can be medically ruled out

### 24.6. Study drug-related actions

The following study drug-related actions are available as responses to occurrence of adverse events:

1. Drug withdrawal

2. Discontinuation
3. Dose reduction
4. Dose increase
5. No change
99. Unknown

### 24.7. Adverse event-related actions

Existence of therapy (a drug therapy or other treatment) and contents of the therapy conducted for developed adverse events should be recorded.

### 24.8. Outcome

Select one of the following 6 categories for adverse event outcomes. Record the date of death if the subject died, and the date of confirmation for relief (in process of recovery), not recovered, or unknown.

1. Recovery
2. Relief (in process of recovery)
3. Recovery with sequelae
4. Not recovered
5. Fatal outcome
99. Unknown (if follow-up investigation impossible for some reason, etc.)

## 25. Follow-Up Investigation of Adverse Events

Follow-up investigation of adverse events will be performed according to the following procedures.

"Recovery" refers to a subject returning to original state of health following an adverse event. For adverse events consisting of aggravation of an existing disease, symptom, or sign during the observation period that was present when consent was granted, "recovery" refers to returning to the health state as of the first day of observation.

1) If the subject has not recovered from an adverse event as of the day of completion of observation or the day of discontinuation, the investigator/subinvestigator will explain follow-up investigations and their purpose to the subject, request cooperation, and perform the investigation within 4 weeks (the day the follow-up investigation is performed is recorded as the follow-up investigation day).
2) If there is no recovery of the adverse event as of the day of the follow-up investigation and a causal relationship between the study drug and event cannot be denied, the investigation should be continued when possible until recovery or stabilization of the adverse event. If a causal relationship between the study drug and event can be denied, the follow-up investigation will conclude on the day the follow-up investigation is performed.
3) If a new SAE for which a causal relationship with the study drug cannot be denied is observed after the day of completion of observation (or the day of discontinuation) and before the follow-up investigation day; or if an adverse event for which a causal relationship with the study drug cannot be

denied that has not been recovered from as of the day of completion of observation (or the day of discontinuation) becomes an SAE; then the investigation should be continued until recovery or stabilization of the adverse event.

4) If an SAE for which a causal relationship with the study drug cannot be denied is discovered after the day of completion of observation (or the day of discontinuation) or after the follow-up investigation day, it will not be investigated in the current study. Therefore, it should be reported according to Article 253 of Enforcement Regulations of the Pharmaceutical Affairs Law, but not through the EDC system.

# 26. Drug Information

Summaries of all study drugs are shown below. Refer to package inserts of each for details.

## 26.1. Aspirin

Aspirin inhibits the cyclooxygenase 1 (COX-1) enzyme, which in turn inhibits synthesis of the thromboxane A2 (TXA2) enzyme, thereby inducing platelet aggregation inhibition. COX-1 inhibitory action in platelets is irreversible because platelets cannot resynthesize the enzyme. However, COX-1 is re-synthesized in blood vessel tissue, so inhibitory effects on synthesis of prostacyclin (PGI2) are reversible and exhibit relatively rapid recovery. In addition, the metabolite salicylic acid does not inhibit COX-1 and does not display platelet aggregation inhibition.

## 26.2. Clopidogrel

The active metabolite of clopidogrel sulfate irreversibly acts on the P2Y12 subtype of platelet ADP receptors, and suppresses platelet aggregation and therefore platelet activity by inhibiting ADP binding.

## 26.3. Cilostazol

Cilostazol exhibits antithrombogenic action by specifically inhibiting phosphodiesterase 3 (PDE3), an enzyme which metabolizes cAMP, thereby inhibiting platelet aggregation. The drug also acts as a vasodilator, and has been reported to increase cerebral blood flow in ischemic stroke patients and blood flow to lower extremities in patients with chronic arterial obstruction.

# 27. Committees

The following types of committees will be established, members of which are described in *28. Study Organization* below.

## 27.1. Steering Committee

This committee is to receive advice from the principal investigator and discuss overall operation of the study while also facilitating the study.

### 27.2. Protocol Authoring Committee

This committee is to receive advice from the principal investigator and members of the Independent Data Monitoring Committee and perform revisions to the study protocol.

### 27.3. Independent Data Monitoring Committee

This committee is to give advice on continuation or discontinuation of the study, as well as revision of the protocol and other issues, based on study progress, serious adverse events, event incidences, results of interim analysis, and other factors. IDMC will perform an interim review based on results of interim analysis with safety information and other data once per year. Beside monthly safety monitoring and yearly safety interim review, the committee will also perform an efficacy and safety interim review once at the mid-point of the study based on interim analysis of inter-group comparisons.

### 27.4. Event Evaluation Committee

This committee is to evaluate validity of subject-related data after an event (primary or secondary endpoint) occurs.

### 27.5. Statistical Analysis Committee

This committee is to suggest and supervise methodologies for group allocation and statistical analysis in the study protocol (establish for target sample size and their rationales, analytical methods, etc.). The committee will also cooperate with the Independent Data Monitoring Committee and the data center in authoring an interim analysis protocol prior to the first interim review. Additionally, the committee will also cooperate with the Independent Data Monitoring Committee and the data center in authoring an analysis protocol prior to the data locked. The committee will also oversee statistics-related materials.

## 28. Study Organization

### 28.1. Study sponsor

Takenori Yamaguchi, President,

Public interest incorporated foundation, Japan Cardiovascular Research Foundation

### 28.2. Principal Investigator

Takenori Yamaguchi, President emeritus, National Cerebral and Cardiovascular Center

### 28.3. Steering Committee

(in alphabetical order)

Shinichirou Uchiyama, Professor, Department of Neurology, Tokyo Women's Medical University (Chairman)

Yasushi Okada, Director, Clinical Research Institute, National Hospital Organization Kyushu Medical Center

Kazumi Kimura, Professor, Department of Stroke Medicine, Kawasaki Medical School

Nobuyuki Sakai, Director, Department of Neurosurgery, Kobe City Medical Center General Hospital

Kazunori Toyoda, Director, Department of Cerebrovascular Medicine, National Cerebral and Cardiovascular Center

Haruhiko Hoshino, Director, Department of Neurology and Stroke Center, Tokyo Saiseikai Central Hospital

### 28.4. Protocol Authoring Committee

(in alphabetical order)

Kazumi Kimura, Professor, Department of Stroke Medicine, Kawasaki Medical School

Kazunori Toyoda, Director, Department of Cerebrovascular Medicine, National Cerebral and Cardiovascular Center (Chairman)

Haruhiko Hoshino, Director, Department of Neurology and Stroke Center, Tokyo Saiseikai Central Hospital

### 28.5. Independent Data Monitoring Committee

(in alphabetical order)

Tatsuya Isomura, Chief executive, CLINICAL STUDY SUPPORT, Inc.

Shinya Goto  Professor, Cardiovascular Medicine, Tokai University School of Medicine

Kotaro Tanaka, Professor, Department of Neurology, University of Toyama (Chairman)

### 28.6. Event Evaluation Committee

(in alphabetical order)

Yasuo Terayama, Professor, Department of Neurology, Iwate Medical University

Teiji Tominaga, Professor, Department of Neurosurgery, Tohoku University

Hidekazu Tomimoto, Professor, Department of Neurology, Mie University

Kiyohiro Houkin, Professor, Department of Neurosurgery, Hokkaido University

Masayasu Matsumoto, Professor, Department of Neurology, Hiroshima University

Kazuo Minematsu, Deputy Director General, National Cerebral and Cardiovascular Center Hospital (Chairman)

Satoshi Yasuda, Director, Department of Cardiovascular Medicine, National Cerebral and Cardiovascular Center

### 28.7. Statistical Analysis Committee

Hideki Orikasa, Professor, Biostatistics and Clinical Epidemiology, University of Toyama Graduate School of Medicine (Chairman)

Naoko Kumagai, Associate Professor, Integrated Center for Advanced Medical Technologies, Kochi Medical School Hospital

### 28.8. Study Secretariat

CSPS.com Study office, Public interest incorporated foundation, Japan Cardiovascular Research Foundation

11F, Nissei Shin-osaka Bldg. 3-4-30 Miyahara, Yodogawa-ku, Osaka 532-0003, Japan

Clinical Coordinating Center, EPS Corporation
TEL: 0120-05-3125 / FAX: 06-4807-3025
E-mail: prj-csps.cont.com@eps.co.jp

### 28.9. Data Center

Clinical Information Division Data Management Center 1, EPS Corporation
Nissei Shin-osaka Bldg., 3-4-30 Miyahara, Yodogawa-ku, Osaka 532-0003, Japan
TEL: 0120-80-0226 / FAX: 06-4807-1117
E-mail: prj-csps-com-etr@e-trial.co.jp

### 28.10. Study Centers

Listed separately.

## 29. References

### 29.1. Literature quoted

1) Chimowitz MI, Lynn MJ, Howlett-Smith H, et al. Comparison of warfarin and aspirin for symptomatic intracranial arterial stenosis. *N Engl J Med* 2005; 352: 1305-16.
2) Kakkos SK, Sabetai M, Tegos T, et al. Silent embolic infarcts on computed tomography brain scans and risk of ipsilateral hemispheric events in patients with asymptomatic internal carotid artery stenosis. *J Vasc Surg* 2009; 49: 902-9
3) Kamouchi M, Kumagai N, Okada Y, Origasa H, Yamaguchi T, Kitazono T. Risk score for predicting recurrence in patients with ischemic stroke: the Fukuoka stroke risk score for Japanese. *Cerebrovasc Dis* 2012; 34: 351-7.
4) Diener HC, Ringleb PA, Savi P. Clopidogrel for the secondary prevention of stroke. *Expert Opin Pharmacother* 2005; 6: 755-64.
5) Diener HC, Cunha L, Forbes C, Sivenius J, Smets P, Lowenthal A. European Stroke Prevention Study. 2. Dipyridamole and acetylsalicylic acid in the secondary prevention of stroke. *J Neurol Sci* 1996; 143: 1-13.
6) The ESPRIT Study Group. Aspirin plus dipyridamole versus aspirin alone after cerebral ischaemia of arterial origin (ESPRIT): randomised controlled trial. *Lancet* 2006; 367: 1665-73.
7) Uchiyama S, Ikeda Y, Urano Y, Horie Y, Yamaguchi T. The Japanese aggrenox (extended-release dipyridamole plus aspirin) stroke prevention versus aspirin programme (JASAP) study: a randomized, double-blind, controlled trial. *Cerebrovasc Dis* 2011; 31: 601-13.
8) Markus HS, Droste DW, Kaps M, et al. Dual antiplatelet therapy with clopidogrel and aspirin in symptomatic carotid stenosis evaluated using doppler embolic signal detection: the Clopidogrel and Aspirin for Reduction of Emboli in Symptomatic Carotid Stenosis (CARESS) trial. *Circulation* 2005; 111: 2233-40.

9) FASTER Investigators. Fast assessment of stroke and transient ischaemic attack to prevent early recurrence (FASTER): a randomised controlled pilot trial. *Lancet Neurol* 2007; 6: 961-9.
10) Wong KS, Chen C, Fu J, et al. Clopidogrel plus aspirin versus aspirin alone for reducing embolisation in patients with acute symptomatic cerebral or carotid artery stenosis (CLAIR study): a randomised, open-label, blinded-endpoint trial. *Lancet Neurol* 2010; 9: 489-97.
11) Wang Y, Wang Y, Zhao X, et al. Clopidogrel with aspirin in acute minor stroke or transient ischemic attack. *N Engl J Med* 2013; 369: 11-9.
12) Diener HC, Bogousslavsky J, Brass LM, et al. Aspirin and clopidogrel compared with clopidogrel alone after recent ischaemic stroke or transient ischaemic attack in high-risk patients (MATCH): randomised, double-blind, placebo-controlled trial. *Lancet* 2004; 364: 331-7.
13) Bhatt DL, Fox KA, Hacke W, et al. Clopidogrel and aspirin versus aspirin alone for the prevention of atherothrombotic events. *N Engl J Med* 2006; 354: 1706-17.
14) The SPS 3 Investigators. Effects of clopidogrel added to aspirin in patients with recent lacunar stroke. *N Engl J Med* 2012; 367: 817-25.
15) Gotoh F, Tohgi H, Hirai S, et al. Cilostazol stroke prevention study: a placebo-controlled double-blind trial for secondary prevention of cerebral infarction. *J Stroke Cerebrovasc Dis* 2000; 9: 147-57.
16) Shinohara Y, Katayama Y, Uchiyama S, et al. Cilostazol for prevention of secondary stroke (CSPS 2): an aspirin-controlled, double-blind, randomised non-inferiority trial. *Lancet Neurol* 2010; 9: 959-68.
17) Kwon SU, Cho YJ, Koo JS, et al. Cilostazol prevents the progression of the symptomatic intracranial arterial stenosis: the multicenter double-blind placebo-controlled trial of cilostazol in symptomatic intracranial arterial stenosis. *Stroke* 2005; 36: 782-6.
18) Kwon SU, Hong KS, Kang DW, et al. Efficacy and safety of combination antiplatelet therapies in patients with symptomatic intracranial atherosclerotic stenosis. *Stroke* 2011; 42: 2883-90.
19) Uchiyama S et al. Final Results of Cilostazol-Aspirin THerapy Against Recurrent Stroke with Intracranial artery Stenosis (CATHARSIS). *38th International Stroke Conference,* Honolulu, USA, Feb.6-8, 2013.
20) Wilhite DB, Comerota AJ, Schmieder FA, Throm RC, Gaughan JP, Rao AK. Managing PAD with multiple platelet inhibitors: the effect of combination therapy on bleeding time. *J Vasc Surg* 2003; 38: 710-3.
21) Hiatt WR, Money SR, Brass EP. Long-term safety of cilostazol in patients with peripheral artery disease: the CASTLE study (Cilostazol: A Study in Long-term Effects). *J Vasc Surg* 2008; 47: 330-6.
22) Sakurai R, Koo BK, Kaneda H, Bonneau HN, Nagai R. Cilostazol added to aspirin and clopidogrel reduces revascularization without increases in major adverse events in patients with drug-eluting stents: A meta-analysis of randomized controlled trials. *Int J Cardiol* 2013; 167: 2250-8.
23) Toyoda K, Yasaka M, Uchiyama S, et al. Blood pressure levels and bleeding events during antithrombotic therapy: the Bleeding with Antithrombotic Therapy (BAT) Study. *Stroke* 2010; 41: 1440-4.
24) The SPS3 Study Group. Blood-pressure targets in patients with recent lacunar stroke: the SPS3

randomised trial. *Lancet* 2013; 382: 507-15.

25) Suzuki N, Sato M, Houkin K, et al.: One-year atherothrombotic vascular events rates in outpatients with recent non-cardioembolic ischemic stroke: the EVEREST (Effective Vascular Event REduction after STroke) registry. *J Stroke Cerebrovasc Dis* 2012; 21: 245-53.
26) Lakatos E, Lan KK. A comparison of sample size methods for the logrank statistic. *Stat Med* 1992; 11: 179-91.
27) Haybittle JL. Repeated assessment of results in clinical trials of cancer treatment. *Br J Radiol* 1971; 44: 793-7.
28) Peto R, Pike MC, Armitage P, et al. Design and analysis of randomized clinical trials requiring prolonged observation of each patient. I. Introduction and design. *Br J Cancer* 1976; 34: 585-612.

## 29.2. Heart diseases which may be source of emboli

High-risk sources of emboli according to classification in the TOAST study (Trial of Org 10172 in Acute Stroke Treatment) are as follows:

Mechanical prosthetic valve, mitral stenosis with atrial fibrillation, atrial fibrillation (other than lone atrial fibrillation), left atrial/atrial appendage thrombus, sick sinus syndrome, recent myocardial infarction (< 4 weeks), left ventricular thrombus, dilated cardiomyopathy, akinetic left ventricular segment, atrial myxoma, infective endocarditis

## 29.3. Definitions of serious hepatic impairment and serious renal impairment

Defined as follows:

i. Serious hepatic impairment: AST and ALT levels both ≥ 2.5 times upper limit of study center standard, or complications of hepatic cirrhosis or active hepatitis

ii. Serious renal impairment: Serum creatinine level ≥ 3 times upper limit of study center standard

## 29.4. Definition of medical history and complications

Defined as follows:

1) Coronary artery disease : Diagnosed as angina pectoris or myocardial infarction at a medical institution
2) Peripheral arterial disease: Comorbid with symptomatic peripheral arterial disease, or ABI < 0.9
3) Hypertension: Systolic blood pressure ≥ 140 mmHg, diastolic blood pressure ≥ 90 mmHg at any examination in the past 3 months, or receiving antihypertensive medication as hypertension treatment
4) Diabetes mellitus: Satisfying any of the following conditions:
   - Fasting plasma glucose ≥ 126 mg/dl
   - Casual blood glucose level ≥ 200 mg/dl
   - 2-hour value in oral glucose tolerance test (75 g dose) of ≥ 200 mg/dl
   - HbA1c (NGSP) ≥ 6.5%
   - Receiving a hypoglycemic agent

・Diagnosed as diabetes mellitus at a medical institution

5) Dyslipidaemia: Satisfying any of the following conditions:

・LDL cholesterol level ≥ 140 mg/dl
・HDL cholesterol level < 40 mg/dl
・Triglyceride ≥ 150 mg/dl
・Receiving a lipid-lowering agent

6) Chronic kidney disease: Any of the following conditions persisting for more than 3 months:

・Clear findings suggesting renal disorder in urinalysis, imaging, pathological diagnosis, or physical findings (especially proteinuria)
・Estimated glomerular filtration rate (eGFR) < 60 mL/min/1.73 $m^2$

## 29.5. Degree of independence in activities of daily living

Evaluate as one of following 7 levels according to Japanese Modified Rankin Scale (Japanese Guidelines for Management of Stroke 2009):

0. No symptoms at all
1. No significant disability despite symptoms: able to carry out all usual duties and activities
2. Slight disability: unable to carry out all previous activities, but able to look after own affairs without assistance
3. Moderate disability: requiring some help, but able to walk without assistance
4. Moderate to severe disability: unable to walk without assistance and unable to attend to own bodily needs without assistance
5. Severe disability: bedridden, incontinent and requiring constant nursing care and attention
6. Dead

## 29.6. GUSTO Criteria

(GUSTO: Global Utilization of Streptokinase and t-PA for Occluded Coronary Arteries Trial)

Defined as follows:

- Severe or life-threatening hemorrhage: Intracranial bleeding or bleeding that causes substantial hemodynamic compromise requiring treatment
- Moderate bleeding: Bleeding which requires blood transfusion
- Minor bleeding: Other bleeding, neither requiring transfusion nor causing hemodynamic compromise

# Study of Antiplatelet Combination Therapy for Ischemic Stroke Patients with High Risk of Recurrence

# CSPS.com

## (Cilostazol Stroke Prevention Study. *Combination*)

## Clinical Study Protocol

Principal investigator: Takenori Yamaguchi, President Emeritus,
National Cerebral and Cardiovascular Center

Protocol Identification Number: 021-TADD-1300-1
Version 1.0: 15 September, 2013
Version 1.1: 17 October, 2013
Version 1.2: 24 February, 2014
Version 1.3: 22 February, 2016

Protocol Summary

| | |
|---|---|
| Study title | Study of antiplatelet combination therapy for ischemic stroke patients with highrisk of recurrence<br>CSPS.com (Cilostazol Stroke Prevention Study. *Combination*) |
| Objectives | To examine the efficacy and safety of dual antiplatelet therapy (DAPT) including cilostazol (Pletaal OD Tablet® & reg.) in comparison with single antiplatelet therapy (SAPT) excluding cilostazol for secondary prevention of ischemic stroke in high-risk patients for stroke |
| Study design | Multicenter, randomized, controlled trial |
| Condition | Noncardioembolic ischemic stroke |
| Inclusion Criteria | Patients must meet all of the following criteria to be eligible for the trial:<br>8) Patients with a diagnosis of noncardioembolic ischemic stroke that developed between 8 and 180 days before the first day of observation<br>9) Patients with a responsible lesion identified by head MRI<br>10) Patients 20 to 85 years of age when providing informed consent<br>11) Patients taking aspirin or clopidogrel alone as antiplatelet therapy when providing informed consent<br>12) Patients meeting at least one of the following criteria:<br>i. At least 50% stenosis of a major intracranial artery<br>ii. At least 50% stenosis of an extracranial artery<br>iii. Two or more of the following risk factors:<br>• 65 years of age or older<br>• Diabetes mellitus<br>• Hypertension<br>• Peripheral arterial disease<br>• Chronic kidney disease<br>• History of symptomatic ischemic stroke (IS) (excluding the index IS for this study)<br>• History of ischemic heart disease<br>• Smoking (only current smokers, excluding previous smokers)<br>13) Patients able to visit the study center throughout the observation period<br>14) Patients who provided written informed consent for participation |
| Exclusion criteria | Patients meeting any of the following criteria will be excluded:<br>17) Patients with emboligenic heart disease<br>18) Patients taking any anticoagulant agents<br>19) Patients who cannot undergo MRI examination for reasons such as |

| | |
|---|---|
| | claustrophobia or implanted pacemaker<br>20) Patients scheduled to undergo any surgery during the study period, including percutaneous angioplasty, stent placement, or bypass grafting<br>21) Patients with a drug-eluting coronary stent implanted within the past year<br>22) Patients with a history of symptomatic non-traumatic intracranial hemorrhage, any other hemorrhagic disease (e.g. active peptic ulcer), bleeding predisposition, or blood clotting disorders<br>23) Patients with a history of hypersensitivity to cilostazol<br>24) Patients with congestive heart failure or uncontrollable angina pectoris<br>25) Patients with thrombocytopenia (platelet count ≤ 100,000/mm$^3$)<br>26) Patients with severe liver or renal dysfunction<br>27) Women who are pregnant, breast-feeding, or of childbearing potential<br>28) Patients with a malignant tumor requiring treatment<br>29) Patients who are taking aspirin, and meet any of the following criteria:<br>• History of hypersensitivity to aspirin or salicylic acid analogues<br>• Peptic ulcer complication<br>• Aspirin-induced asthma or its history<br>30) Patients who take clopidogrel, and meet the following criterion:<br>• History of hypersensitivity to clopidogrel<br>31) Patients who are participating in any other clinical studies<br>32) Patients considered by the investigator/subinvestigator to be unsuitable for participating in this study |
| Endpoints | Primary endpoint<br>• Recurrence of symptomatic ischemic stroke ("ischemic stroke" hereafter), with symptoms lasting for at least 24 hours<br><br>Secondary endpoints<br>• Any stroke [ischemic stroke (IS), intracerebral hemorrhage (ICH), or subarachnoid hemorrhage (SAH)]<br>• ICH or SAH<br>• Ischemic cerebrovascular disorder [IS or transient ischemic attack (TIA)]<br>• Death from any cause<br>• Stroke [IS, ICH, SAH], myocardial infarction (MI), or vascular death<br>• All vascular events: stroke, MI, and other vascular events [e.g. aortic dissection or rupture; pulmonary embolism; heart failure, angina pectoris, or occlusive arteriosclerosis requiring hospitalization; and revascularization of coronary artery, aorta, peripheral artery, etc.] |

| | |
|---|---|
| | Safety endpoints<br>• Adverse events and adverse drug reactions<br>• Severe or life-threatening hemorrhage (GUSTO Criteria) |
| Dosage, administration, and treatment period | SAPT group<br>One of the following drugs will be administered:<br>Aspirin: 81 or 100 mg/day (once daily)<br>Clopidogrel: 50 or 75 mg/day (once daily)<br>DAPT group<br>One of the following drugs will be administered:<br>Aspirin: 81 or 100 mg/day (once daily)<br>Clopidogrel: 50 or 75 mg/day (once daily)<br><br>As a rule, cilostazol (Pletaal OD Tablet® & reg.) will be co-administered twice daily at the maintenance dose of 200 mg/day. However, the starting dose can be 100 mg/day, in which case the dose must fundamentally be increased to 200 mg/day within 15 days. |
| Target sample size | 4,000 |
| Study period | October 2013 to March 2018 |

## Study Design

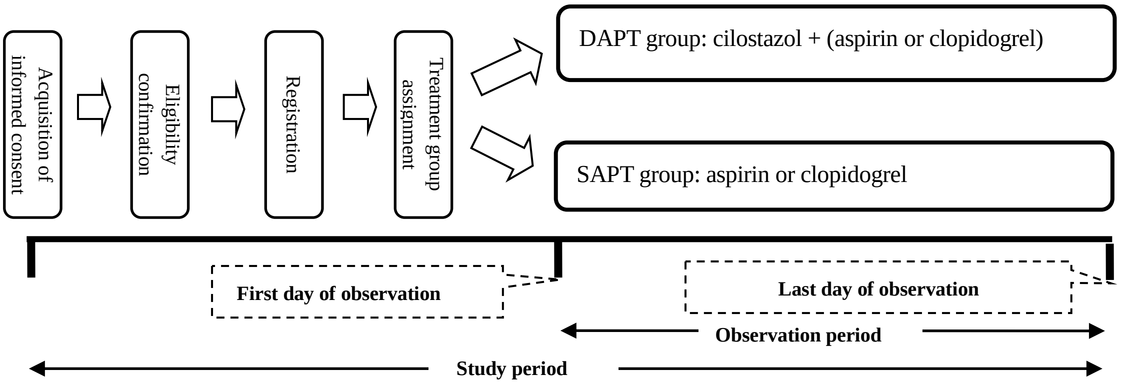


## Observation schedule

| Evaluation items \ Time point | (Date of onset) | Enroll-ment | Start of obser-vation | Month 1 | Month 3 | Month 6 | Month 12 | Every 6 months there-after | End of obser-Vation (*) |
|---|---|---|---|---|---|---|---|---|---|
| Informed consent | ← | ○ | | | | | | | |
| Enrollment | | ○ | | | | | | | |
| Observation status | | | | ○ | ○ | ○ | ○ | ○ | ○ |
| Patient characteristics | ← | ○ | | | | | | | |
| Degree of independence in daily living (mRS) | | | ○ | ○ | ○ | ○ | ○ | ○ | ○ |
| Study drug administration/ compliance | | | ○ | ○ | ○ | ○ | ○ | ○ | ○ |
| Drugs other than study drug | | | ○ | ○ | ○ | ○ | ○ | ○ | ○ |
| Blood pressure | | | ○ | ○ | ○ | ○ | ○ | ○ | ○ |
| Head MRI | ← | ○ | | | | | | | |
| Head MRI (T2* WI) | ← | △ | → | | | | | | |
| Head MRA | ← | △ | → | | | | | | |
| Carotid artery imaging | ← | △ | → | | | | | | |
| Laboratory test (blood) | ← | ○ | → | ←······ | ······ | ······ | ······ | ······ | ······→ |
| Laboratory test (urine) | ← | ○ | → | ←······ | ······ | ······ | ······ | ······ | ······→ |
| Chest x-ray | ← | ○ | → | ←······ | ······ | ······ | ······ | ······ | ······→ |
| ECG | ← | ○ | → | ←······ | ······ | ······ | ······ | ······ | ······→ |
| Adverse events | | | ←—— | —— | —— | —— | —— | —— | ——→ |

○ Required item △ Optional item ←······→ As required

* Observation is complete when a subject meets criteria for discontinuation/completion, or when 1 year has elapsed from initiation of observation for last subject enrolled.

## Table of Contents

**1. STUDY OBJECTIVES** ...................................................... エラー! ブックマークが定義されていません。

**2. BACKGROUND AND RATIONALE** ............................ エラー! ブックマークが定義されていません。

2.1. PRESENT STATE OF DUAL ANTIPLATELET THERAPY IN PREVENTING ISCHEMIC STROKE RECURRENCE. エラー! ブックマークが定義されていません。

2.2. COMBINED CILOSTAZOL-ASPIRIN THERAPY IN PATIENTS WITH ISCHEMIC STROKE.. エラー! ブックマークが定義されていません。

2.3. DUAL ANTIPLATELET THERAPY USING CILOSTAZOL IN PATIENTS WITH CARDIOVASCULAR DISEASE エラー! ブックマークが定義されていません。

**3. PATIENT SELECTION**.................................................... エラー! ブックマークが定義されていません。

3.1. TARGET DISEASE ........................................................ エラー! ブックマークが定義されていません。

3.2. INCLUSION CRITERIA ....................................................... エラー! ブックマークが定義されていません。

3.3. EXCLUSION CRITERIA ..................................................... エラー! ブックマークが定義されていません。

**4. ENROLLMENT AND ALLOCATION** .......................... エラー! ブックマークが定義されていません。

4.1. ENROLLMENT AND ALLOCATION ...................................... エラー! ブックマークが定義されていません。

4.2. ALLOCATION METHODOLOGY ........................................... エラー! ブックマークが定義されていません。

**5. TREATMENT PLAN AND CRITERIA FOR CHANGES IN DOSAGE OR SCHEDULE**..エラー! ブックマークが定義されていません。

5.1. PROTOCOL TREATMENT...................................................... エラー! ブックマークが定義されていません。

5.2. CRITERIA FOR CHANGES IN DOSAGE OR TREATMENT SCHEDULE.エラー! ブックマークが定義されていません。

5.3. CRITERIA FOR SUBJECT DISCONTINUATION OR COMPLETIONエラー! ブックマークが定義されていません。

5.4. CONCOMITANT THERAPIES ................................................. エラー! ブックマークが定義されていません。

**6. STUDY PROCEDURES**................................................... エラー! ブックマークが定義されていません。

6.1. OBSERVATIONS/TESTS AND OBSERVATION SCHEDULE .......エラー! ブックマークが定義されていません。

6.2. OBSERVATION DAYS ........................................................ エラー! ブックマークが定義されていません。

6.3. OBSERVATION, TEST, AND REPORT ITEMS.......................... エラー! ブックマークが定義されていません。

**7. ADVERSE EVENTS**......................................................... エラー! ブックマークが定義されていません。

7.1. DEFINITION ...................................................................... エラー! ブックマークが定義されていません。

7.2. PROCEDURES FOR ADVERSE EVENTS ................................ エラー! ブックマークが定義されていません。

7.3. EVALUATION OF ADVERSE EVENTS.................................... エラー! ブックマークが定義されていません。

7.4. FOLLOW-UP OF ADVERSE EVENTS...................................... エラー! ブックマークが定義されていません。

7.5. EXPECTED ADVERSE DRUG REACTIONS ............................エラー! ブックマークが定義されていません。
7.6. PROCEDURES FOR SUBJECTS WHO ARE SUSPECTED OR KNOWN TO BE PREGNANT .. エラー! ブックマークが定義されていません。

**8. EFFICACY EVALUATION** .......................................... エラー! ブックマークが定義されていません。

8.1. PRIMARY ENDPOINT ..........................................................エラー! ブックマークが定義されていません。
8.2. SECONDARY ENDPOINTS...................................................エラー! ブックマークが定義されていません。

**9. SAFETY EVALUATION** ............................................... エラー! ブックマークが定義されていません。

**10. TARGET SAMPLE SIZE AND STUDY PERIOD** ...... エラー! ブックマークが定義されていません。

10.1. TARGET SAMPLE SIZE .....................................................エラー! ブックマークが定義されていません。
10.2. STUDY PERIOD................................................................エラー! ブックマークが定義されていません。

**11. STATISTICAL ANALYSIS**.......................................... エラー! ブックマークが定義されていません。

11.1. RATIONALE FOR TARGET SAMPLE SIZE ............................エラー! ブックマークが定義されていません。
11.2. ANALYSIS POPULATIONS ................................................エラー! ブックマークが定義されていません。
11.3. ANALYSIS ITEMS AND METHODS ......................................エラー! ブックマークが定義されていません。
11.4. INTERIM REVIEW ............................................................エラー! ブックマークが定義されていません。
11.5. FINAL ANALYSIS ............................................................エラー! ブックマークが定義されていません。

**12. CASE REPORT FORM DATA ENTRY AND SUBMISSION** エラー! ブックマークが定義されていません。

**13. DATA MANAGEMENT** ............................................... エラー! ブックマークが定義されていません。

**14. ETHICS**......................................................................... エラー! ブックマークが定義されていません。

14.1. RULES AND REGULATIONS TO BE OBSERVED ....................エラー! ブックマークが定義されていません。
14.2. SUBJECT BENEFITS AND DRAWBACKS ..............................エラー! ブックマークが定義されていません。
14.3. REVIEW AND DISCUSSION BY ETHICS COMMITTEE ............エラー! ブックマークが定義されていません。
14.4. AUTHORING AND REVISION OF INFORMED CONSENT FORMS AND WRITTEN INFORMATION FOR SUBJECTS ... エラー! ブックマークが定義されていません。
14.5. INFORMED CONSENT........................................................エラー! ブックマークが定義されていません。
14.6. PRIVACY PROTECTIONS AND SUBJECT IDENTIFICATION .....エラー! ブックマークが定義されていません。

**15. STUDY FUNDING AND CONFLICTS OF INTEREST**エラー! ブックマークが定義されていません。

**16. STUDY EXPENSES** ...................................................... エラー! ブックマークが定義されていません。

**17. RESPONSES AND COMPENSATION FOR DAMAGE TO SUBJECT HEALTH** . エラー! ブックマークが定義されていません。

17.1. RESPONSES TO DAMAGE TO HEALTH ................................エラー! ブックマークが定義されていません。
17.2. CLINICAL STUDY INSURANCE ..........................................エラー! ブックマークが定義されていません。

**18. REVISION OF THE STUDY PROTOCOL** ................. エラー! ブックマークが定義されていません。

**19. STUDY COMPLETION AND EARLY DISCONTINUATION** .. エラー! ブックマークが定義されていません。

19.1. STUDY COMPLETION AT EACH STUDY CENTER .................. エラー! ブックマークが定義されていません。

19.2. STUDY CANCELLATION OR DISCONTINUATION AT EACH STUDY CENTER エラー! ブックマークが定義されていません。

19.3. OVERALL COMPLETION OF STUDY .................................... エラー! ブックマークが定義されていません。

19.4. EARLY DISCONTINUATION OF STUDY ................................ エラー! ブックマークが定義されていません。

**20. STORAGE OF STUDY-RELATED MATERIALS** ...... エラー! ブックマークが定義されていません。

**21. CLINICAL STUDY REGISTRATION** ......................... エラー! ブックマークが定義されていません。

**22. AUTHORING OF CLINICALSTUDY REPORT, DISCLOSURE OF STUDY RESULTS, AND OWNERSHIP OF RESULTS AND INTELLECTUAL PROPERTY** エラー! ブックマークが定義されていません。

**23. PROCEDURES FOR ADVERSE EVENTS** ................. エラー! ブックマークが定義されていません。

23.1. SUBJECT-RELATED RESPONSE .......................................... エラー! ブックマークが定義されていません。

23.2. SERIOUS ADVERSE EVENT REPORTING .............................. エラー! ブックマークが定義されていません。

**24. EVALUATION OF ADVERSE EVENTS** ...................... エラー! ブックマークが定義されていません。

24.1. EVENT NAME .................................................................... エラー! ブックマークが定義されていません。

24.2. EVENT DATE ..................................................................... エラー! ブックマークが定義されていません。

24.3. SEVERITY ......................................................................... エラー! ブックマークが定義されていません。

24.4. SERIOUSNESS .................................................................. エラー! ブックマークが定義されていません。

24.5. CAUSAL RELATIONSHIP WITH STUDY DRUGS ..................... エラー! ブックマークが定義されていません。

24.6. STUDY DRUG-RELATED ACTIONS ...................................... エラー! ブックマークが定義されていません。

24.7. ADVERSE EVENT-RELATED ACTIONS ................................. エラー! ブックマークが定義されていません。

24.8. OUTCOME ......................................................................... エラー! ブックマークが定義されていません。

**25. FOLLOW-UP INVESTIGATION OF ADVERSE EVENTS** エラー! ブックマークが定義されていません。

**26. DRUG INFORMATION** ................................................ エラー! ブックマークが定義されていません。

26.1. ASPIRIN ............................................................................ エラー! ブックマークが定義されていません。

26.2. CLOPIDOGREL .................................................................. エラー! ブックマークが定義されていません。

26.3. CILOSTAZOL .................................................................... エラー! ブックマークが定義されていません。

**27. COMMITTEES** ............................................................. エラー! ブックマークが定義されていません。

27.1. STEERING COMMITTEE ..................................................... エラー! ブックマークが定義されていません。

27.2. PROTOCOL AUTHORING COMMITTEE ................................ エラー! ブックマークが定義されていません。

27.3. INDEPENDENT DATA MONITORING COMMITTEE ...............エラー! ブックマークが定義されていません。
27.4. EVENT EVALUATION COMMITTEE....................................エラー! ブックマークが定義されていません。
27.5. STATISTICAL ANALYSIS COMMITTEE ...............................エラー! ブックマークが定義されていません。

**28. STUDY ORGANIZATION.............................................** エラー! ブックマークが定義されていません。

28.1. STUDY SPONSOR..............................................................エラー! ブックマークが定義されていません。
28.2. PRINCIPAL INVESTIGATOR..................................................エラー! ブックマークが定義されていません。
28.3. STEERING COMMITTEE .....................................................エラー! ブックマークが定義されていません。
28.4. PROTOCOL AUTHORING COMMITTEE...............................エラー! ブックマークが定義されていません。
28.5. INDEPENDENT DATA MONITORING COMMITTEE ...............エラー! ブックマークが定義されていません。
28.6. EVENT EVALUATION COMMITTEE....................................エラー! ブックマークが定義されていません。
28.7. STATISTICAL ANALYSIS COMMITTEE ...............................エラー! ブックマークが定義されていません。
28.8. STUDY SECRETARIAT .........................................................エラー! ブックマークが定義されていません。
28.9. DATA CENTER ..................................................................エラー! ブックマークが定義されていません。
28.10. STUDY CENTERS ..............................................................エラー! ブックマークが定義されていません。

**29. REFERENCES................................................................** エラー! ブックマークが定義されていません。

29.1. LITERATURE QUOTED .......................................................エラー! ブックマークが定義されていません。
29.2. HEART DISEASES WHICH MAY BE SOURCE OF EMBOLI .......エラー! ブックマークが定義されていません。
29.3. DEFINITIONS OF SERIOUS HEPATIC IMPAIRMENT AND SERIOUS RENAL IMPAIRMENT . エラー! ブックマークが定義されていません。
29.4. DEFINITION OF MEDICAL HISTORY AND COMPLICATIONS ..エラー! ブックマークが定義されていません。
29.5. DEGREE OF INDEPENDENCE IN ACTIVITIES OF DAILY LIVINGエラー! ブックマークが定義されていません。
29.6. GUSTO CRITERIA............................................................エラー! ブックマークが定義されていません。

## 1. Study Objectives

To evaluate the efficacy and safety of dual antiplatelet therapy (DAPT) including cilostazol (Pletaal OD Tablet®) in comparison with single antiplatelet therapy (SAPT) excluding cilostazol for secondary prevention of ischemic stroke in high-risk patients.

## 2. Background and Rationale

### 2.1. Present state of dual antiplatelet therapy in preventing ischemic stroke recurrence

Currently, treatment with antiplatelet drugs is strongly recommended for preventing recurrence of noncardioembolic ischemic stroke, along with improvement of the lifestyle and management of risk factors. However, prophylactic efficacy of aspirin for vascular events, for which there is significant evidence, shows a relative risk reduction rate (RRR) of only 20 to 30%. Existing antiplatelet monotherapy does not have sufficient prophylactic efficacy for stroke recurrence, especially in patients with overlapping risk factors such as intracranial artery stenosis, carotid stenosis, and diabetes mellitus/hypertension[1–5]. Therefore, stronger antiplatelet drugs development or dual antiplatelet therapies with different mechanism of action are being examined to enhance prophylactic efficacy for vascular events in patients at high risk for ischemic stroke (IS) or transient ischemic attack (TIA).

With regard to dual antiplatelet therapy, efficacy of combined aspirin and extended-release dipyridamole in IS/TIA patients was confirmed in the ESPS 2 (European Stroke Prevention Study)[6] and ESPRIT (European/Australasian Stroke Prevention in Reversible Ischaemia Trial)[7] studies. However, in Japan, the JASAP study [Japanese Aggrenox (Extended-Release Dipyridamole plus Aspirin) Stroke Prevention versus Aspirin Programme][8] failed to demonstrate the efficacy of this combination, and dipyridamole has not been approved to prevent recurrence of ischemic stroke.

With regard to the combined effect of aspirin and clopidogrel in IS/TIA patients, results of CARESS (Clopidogrel and Aspirin for Reduction of Emboli in Symptomatic Carotid Stenosis)[9], FASTER (Fast Assessment of Stroke and Transient ischaemic attack to prevent Early Recurrence)[10], CLAIR (Clopidogrel plus aspirin versus aspirin alone for reducing embolisation in patients with acute symptomatic cerebral or carotid artery stenosis)[11], etc., demonstrated the efficacy of combined treatment in patients with IS/TIA at relatively early stage of onset. Efficacy of a short-term combination therapy of aspirin and clopidogrel was also demonstrated in the recent CHANE study (Clopidogrel in High-risk patients with Acute Non-disabling Cerebrovascular Events)[12]. However, long-term combined use of aspirin and clopidogrel[13–15] is not recommended as it increases serious bleeding complications, even though there is no change or even a slight decrease observed in vascular events.

### 2.2.Combined cilostazol-aspirin therapy in patients with ischemic stroke

Cilostazol, developed in Japan, significantly inhibited ischemic stroke recurrence and did not increase serious bleeding in a double-blind placebo-controlled study in patients with ischemic stroke (CSPS, Cilostazol Stroke Prevention Study)[16]. Similarly, cilostazol significantly inhibited strokes (ischemic stroke, intracerebral

hemorrhage, and subarachnoid hemorrhage) and reduced serious bleeding to half in a double-blind controlled study with aspirin (CSPS 2, Cilostazol Stroke Prevention Study 2)[17].

The combination therapy of aspirin and cilostazol in patients with ischemic stroke has been tested in three small-scale studies, and no studies demonstrated any significant increase compared with aspirin monotherapy in serious bleeding complications. Summaries of the three studies are presented below.

In the TOSS study (Trial of Cilostazol in Symptomatic Intracranial Arterial Stenosis)[18] examining the inhibitory effects of aspirin or combined aspirin-cilostazol therapy on stenosis progression in patients with intracranial arterial stenosis, combined aspirin-cilostazol therapy significantly inhibited progression of stenosis compared to aspirin monotherapy. There was no increase in intracerebral hemorrhage due to the combination therapy, and results suggested effectiveness and safety of combined aspirin and cilostazol. However, the observation period in this study was only 6 months.

The subsequent TOSS 2 study (Trial of Cilostazol in Symptomatic Intracranial Arterial Stenosis)[19] examined the inhibitory effects of combined aspirin and clopidogrel versus aspirin and cilostazol on stenosis progression in patients with intracranial arterial stenosis. The study failed to demonstrate significant inhibition of stenosis progression, but serious bleeding was less frequent (0.9%) in the aspirin and cilostazol group compared to the aspirin and clopidogrel group (2.6%).

The CATHARSIS study (Cilostazol-Aspirin Therapy Against Recurrent Stroke with Intracranial artery Stenosis)[20] followed stenosis progression and stroke recurrence in an aspirin monotherapy group and an aspirin-cilostazol combination group in patients with intracranial arterial stenosis for 2 years. No significant differences were observed in intracranial artery stenosis progression, perhaps due to a background factor bias. However, strokes were less frequent in the aspirin-cilostazol combination group (2.5%/year) than the aspirin monotherapy group (4.5%/year), and there was no increase in serious bleeding in the combination group compared to the monotherapy group. This set of results has demonstrated that combination therapy using cilostazol is superior in safety compared with conventional dual antiplatelet therapy.

## 2.2. Dual antiplatelet therapy using cilostazol in patients with cardiovascular disease

With regard to cardiovascular diseases other than strokes, an overseas study has reported that the combination of cilostazol and other antiplatelet drugs does not increase bleeding complications.

A crossover study examining the effects of various combination therapies utilizing 3 antiplatelet drugs on bleeding time in patients with peripheral arterial disease[21] showed a significant prolongation of bleeding time with the aspirin-clopidogrel combination compared to monotherapy with each drug. However, no significant prolongation of bleeding time was observed with cilostazol in combination with either aspirin or clopidogrel. Moreover, no prolongation of bleeding time was observed when cilostazol was added to the aspirin-clopidogrel combination therapy.

The CASTLE study (Cilostazol: A Study in Long-term Effects)[22] was conducted with the primary objective of evaluating safety in patients with peripheral arterial disease. The effects of cilostazol and placebo were compared in 1,439 patients for a mean observation period of about 1.5 years. About 70% of patients used aspirin and about 27% used clopidogrel concomitantly in both placebo and cilostazol groups, but no

difference was observed in serious bleeding events between the two groups.

A meta-analysis[23] examined prophylactic effects on stenosis recurrence and safety following drug-eluting stent implantation in patients with coronary artery disease. No significant difference in bleeding events or serious bleeding events was observed between groups receiving either aspirin and clopidogrel or a 3-drug combination with cilostazol added.

These reports suggest that the adding either aspirin or clopidogrel to cilostazol use does not increase risk of bleeding complications.

Based on these clinical results, the current study will examine prophylactic efficacy on ischemic stroke recurrence between SAPT group and DAPT group with cilostazol added to conventional antiplatelet therapy in high risk patients with at least two risk factors of intracranial arterial stenosis, carotid stenosis, or ischemic stroke, with observation period at least one year.

# 3. Patient Selection

## 3.1. Target disease

Noncardioembolic ischemic stroke

## 3.2. Inclusion criteria

Patients meeting all of the following criteria will be included:

1) Patients with a diagnosis of noncardioembolic ischemic stroke that developed between 8 and 180 days before the first day of observation
2) Patients with a responsible lesion identified by head MRI
3) Patients 20 to 85 years of age when providing informed consent
4) Patients taking aspirin or clopidogrel alone as antiplatelet therapy when providing informed consent
5) Patients meeting at least one of the following criteria:
   i. At least 50% stenosis of a major intracranial artery (to the level of A2, M2, P2)
   ii. At least 50% stenosis of an extracranial artery*
      * common carotid artery, internal carotid artery, vertebral artery, brachiocephalic artery, or subclavian artery
   iii. Two or more of the following risk factors:
      - 65 years of age or older
      - Diabetes mellitus
      - Hypertension
      - Peripheral arterial disease
      - Chronic kidney disease
      - History of symptomatic ischemic stroke (excluding the index IS for this study)
      - History of ischemic heart disease
      - Smoking (only current smokers, excluding previous smokers)
6) Patients able to visit the study center throughout the observation period

7) Patients who provided written informed consent for participation

*Rationale for inclusion criteria*

1) A minimum of 8 days after onset was set because symptoms should be stable to a certain extent, and a maximum of 180 days after onset was set because recurrence rate decreases gradually over time.
2) To confirm the diagnosis of ischemic stroke
3) A minimum age of 20 years was set because that is required for valid consent, and a maximum age of 85 years was set because of the length of the observation period.
4) Patients taking aspirin or clopidogrel alone when providing informed consent were considered suitable based on the study design.
5) The following criteria were established to determine high risk of stroke recurrence:
    i. Based on the WASID trial sub-analysis report[2]
    ii. Based on the ACSRS study sub-analysis report[3]
    iii. Based on the Stroke Prevention Model[5], the Fukuoka Stroke Risk Score[4] and the CAPRIE trial sub-analysis.
6) Established in order for the patients to be able to undergo planned observation and tests.
7) Complied with the “Ethical Guideline for Clinical Studies.”

### 3.3. Exclusion Criteria

Patients meeting any of the following criteria will be excluded:

1) Patients with emboligenic heart disease (See *29. References*)
2) Patients taking any anticoagulant agents
3) Patients who cannot undergo MRI examination for reasons such as claustrophobia or implanted pacemaker
4) Patients scheduled to undergo any surgery during the study period, including percutaneous angioplasty, stent placement, or bypass grafting
5) Patients with a drug-eluting coronary stent implanted within the past year
6) Patients with a history of symptomatic non-traumatic intracranial hemorrhage, any other hemorrhagic disease (e.g. active peptic ulcer), bleeding predisposition, or blood clotting disorders
7) Patients with a history of hypersensitivity to cilostazol
8) Patients with congestive heart failure or uncontrolled angina pectoris
9) Patients with thrombocytopenia (platelet count ≤ 100,000/$mm^3$)
10) Patients with severe liver or renal dysfunction (See *29. References*)
11) Women who are pregnant, breast-feeding, or of child-bearing potential
12) Patients with malignant tumor requiring treatment
13) Patients who are taking aspirin and meet any of the following criteria:
    - History of hypersensitivity to aspirin or salicylic acid analogues
    - Peptic ulcer complication
    - Aspirin-induced asthma or its history

14) Patients who are taking clopidogrel, and meet the following criterion:
- History of hypersensitivity to clopidogrel

15) Patients who are participating in any other clinical studies
16) Patients considered by the investigator/subinvestigator to be unsuitable for this study

*Rationale for exclusion criteria*

1) To eliminate risk of enrolling patients with cardioembolic ischemic stroke
2) Anticoagulant agents are specified as prohibited concomitant medications
3) Prevents specified examinations
4) Evaluation will be strongly affected by these surgeries
5) Dual antiplatelet therapy is necessary for one year after the drug-eluting stent implantation
6) to 16) To secure the safety of study subjects based on the precautions for using cilostazol, aspirin, and clopidogrel

# 4. Enrollment and Allocation

## 4.1. Enrollment and allocation

The investigator/subinvestigator must obtain written informed consent from all patients prior to study enrollment. After obtaining written informed consent, the investigator/subinvestigator must assign a uniformly-determined subject identification code (see *14.6 Privacy protections and subject identification*) and enroll the subject through the Internet enrollment system. After enrollment, the investigator/subinvestigator must find out which treatment group the subject was allocated to from the enrollment system. For details, refer to the *Subject enrollment manual*.

The following items must be input into the system when subjects are enrolled:

Subject identification code
Date of written informed consent
Sex
Date of birth
Date of most recent noncardioembolic IS
Eligibility information (confirmation of inclusion and exclusion criteria)
Scheduled date of first visit

## 4.2. Allocation methodology

The enrollment center will allocate subjects to treatment groups using blocked randomization according to study center.

# 5. Treatment plan and criteria for changes in dosage or schedule

## 5.1. Protocol treatment

### 5.1.1. Dosage and administration

SAPT group

One of the following drugs will be administered:

| | |
|---|---|
| Aspirin: | 81 or 100 mg/day (once daily) |
| Clopidogrel: | 50 or 75 mg/day (once daily) |

DAPT group

One of the following drugs will be administered:

| | |
|---|---|
| Aspirin: | 81 or 100 mg/day (once daily) |
| Clopidogrel: | 50 or 75 mg/day (once daily) |

As a rule, cilostazol (Pletaal OD tablet® & reg.) will be co-administered twice daily at the maintenance dose of 200 mg/day. However, the starting dose can be 100 mg/day, in which case the dose must fundamentally be increased to 200 mg/day within 15 days.

Cilostazol, aspirin and clopidogrel are referred to below as study drugs.

### 5.1.2. Treatment initiation timing

Treatment with cilostazol will be initiated on the first day of observation.

Type or dosage of aspirin or clopidogrel will not be changed after obtaining written informed consent.

### 5.1.3. Confirming treatment status

The investigator/subinvestigator must confirm subject's treatment status by interview and record it on his/her medical record.

## 5.2. Criteria for changes in dosage or treatment schedule

Dosage or treatment schedule of aspirin or clopidogrel will fundamentally not be changed during the observation period.

If a headache etc. occurs after treatment with cilostazol at a dose of 200 mg/day, the treatment will continue at a reduced dosage of 100 mg/day.

Study drug may be withdrawn in event of surgery or other invasive procedures, but the period of drug withdrawal may not last more than 4 weeks.

## 5.3. Criteria for subject discontinuation or completion

If it is determined that continued observation of a subject is impossible due to the following reasons, that subject will be withdrawn from the study and the reason and date for this recorded. All necessary observations and tests will be performed at this time, and efficacy and safety evaluations performed.

1) Subject requests discontinuation or withdraws consent
2) A serious adverse event occurs which makes continuation problematic
3) Any event described in 6.3.3. occurs

4) Drug withdrawal lasts longer than 4 weeks
5) Any other reasons which lead the investigator/subinvestigator to consider continued participation problematic

Observation will also be discontinued for patients who discontinued taking study drugs.

## 5.4. Concomitant therapies

Cardiovascular risk factors, including hypertension, dyslipidemia, and diabetes mellitus will be controlled according to generally recommended guidelines using any type of drug. There are no restrictions for physical therapy or diet therapy.

Blood pressure should preferably be controlled with a target systolic blood pressure of ≤130 mmHg in order to inhibit intracranial hemorrhage.[24, 25]

Prohibited concomitant medications

Concomitant use of antiplatelet and anticoagulant drugs other than the study drugs is prohibited.

Antiplatelet drugs

Sarpogrelate hydrochloride, dipyridamole, beraprost sodium, limaprost alfadex, alprostadil, ozagrel sodium, ticlopidine, plasugrel, and combined drugs containing aspirin or clopidogrel

Anticoagulants

Warfarin potassium, dabigatran, edoxaban, rivaroxaban, apixaban, heparin products (except for topical external preparation), low-molecular-weight heparin, danaparoid sodium, argatroban, urinastatin, gabexate mesilate, nafamostat mesilate, antithrombin III (freeze-dried concentrated), and human activated protein C (freeze-dried concentrated)

Concomitant medications requiring caution

Caution must be exercised when the following drugs are used concomitantly. For details, refer to the insert information of each product.

A) Aspirin group

Gout drugs, diabetes mellitus drugs, methotrexate, lithium products, thiazide diuretics, furosemide, azosemide, piretanide, nonsteroidal anti-inflammatory analgesics, ibuprofen, oxicam anti-inflammatory analgesics, alminoprofen, nitroglycerin products, tetracycline antibiotics, new quinolone antibacterials, adrenocortical hormone drugs, sodium lactate, sodium valproate, phenytoin, acetazolamide, angiotensin converting enzyme inhibitors, and β-blockers

B) Clopidogrel group

Nonsteroidal anti-inflammatory analgesics and thrombolytic drugs

Drug-metabolizing enzyme (CYP2C19) inhibitors: omeprazole etc.

C) DAPT group only

Drug-metabolizing enzyme (CYP3A4) inhibitors: macrolide antibiotics, HIV protease inhibitors, azole antifungals, cimetidine, diltiazem, grapefruit juice, etc.

Drug-metabolizing enzyme (CYP2C19) inhibitors: omeprazole etc.

# 6. Study Procedures

## 6.1. Observations/tests and observation schedule

Subject observation will follow the schedule below. Timing of each observation is based on the first visit.

| Time point / Evaluation item | (Date of onset) | Enroll-ment | Start of obser-vation | Month 1 | Month 3 | Month 6 | Month 12 | Every 6 months there-after | End of obser-Vation (*) |
|---|---|---|---|---|---|---|---|---|---|
| Informed consent | ← | ○ | | | | | | | |
| Enrollment | | ○ | | | | | | | |
| Observation status | | | | ○ | ○ | ○ | ○ | ○ | ○ |
| Patient characteristics | ← | ○ | | | | | | | |
| Degree of independence in daily living (mRS) | | | ○ | ○ | ○ | ○ | ○ | ○ | ○ |
| Study drug administration/ compliance | | | ○ | ○ | ○ | ○ | ○ | ○ | ○ |
| Drugs other than study drug | | | ○ | ○ | ○ | ○ | ○ | ○ | ○ |
| Blood pressure | | | ○ | ○ | ○ | ○ | ○ | ○ | ○ |
| Head MRI | ← | ○ | | | | | | | |
| Head MRI (T2* WI) | ← | △ | → | | | | | | |
| Head MRA | ← | △ | → | | | | | | |
| Carotid artery imaging | ← | △ | → | | | | | | |
| Laboratory test (blood) | ← | ○ | → | ←⋯ | ⋯ | ⋯ | ⋯ | ⋯ | ⋯→ |
| Laboratory test (urine) | ← | ○ | → | ←⋯ | ⋯ | ⋯ | ⋯ | ⋯ | ⋯→ |
| Chest x-ray | ← | ○ | → | ←⋯ | ⋯ | ⋯ | ⋯ | ⋯ | ⋯→ |
| ECG | ← | ○ | → | ←⋯ | ⋯ | ⋯ | ⋯ | ⋯ | ⋯→ |
| Adverse events | | | ←— | — | — | — | — | — | —→ |

○ Required item △ Optional item ←⋯→ As required

* Observation is complete when a subject meets criteria for discontinuation/completion, or when 1 year has elapsed from initiation of observation for last subject enrolled.

## 6.2. Observation days

### 6.2.1. First day of observation

The first day of observation is the first visit made by an enrolled subject after allocation, and shall take place within 30 days of the enrollment date.

The first day of observation for subjects in the SAPT group is the day when tests etc. described in *6.3.1. First day of observation* are complete.

### 6.2.2. At 1, 3, 6, and 12 months

Subjects will be observed at 1, 3, 6, and 12 months (determined days) following the first day of observation.

### 6.2.3. From 12 months onward

Subjects will be observed every 6 months.

### 6.2.4. Acceptable variation of days of observation

Acceptable variations in days of observation are as follows.

| | |
|---|---|
| Month 1 | ± 2 weeks from determined day |
| Month 3 | ± 4 weeks from determined day |
| Month 6 | ± 8 weeks from determined day |
| Month 12 and every 6 months thereafter | ± 8 weeks from determined day |

### 6.2.5. Completion of observation

Observation will be completed for all subjects at 1 year after observation begins or the last subject enrolled in the study.

## 6.3. Observation, test, and report items

### 6.3.1. First day of observation

The following items, including patient background, will be observed. All test data from day of onset to first day of observation will be used.

- Date of first visit
- Age and sex
- Height (cm)
- Weight (kg)
- Date of most recent noncardioembolic ischemic stroke
- Medical history and complications

  Coronary artery disease: No or Yes

  Peripheral arterial disease: No or Yes

  Hypertension: No or Yes

  Diabetes mellitus: No or Yes

  Dyslipidemia: No or Yes

  Chronic kidney disease: No or Yes

  History of ischemic stroke (excluding the index ischemic stroke for this study): No or Yes
- Smoking habit: No (nonsmokers or previous smokers) or Yes (current smokers of ≥1 cigarette/day)
- Modified Rankin Scale (mRS) (see *29.5 Modified Rankin Scale*)
- Statues of study drug treatment

  Drug name, prescription start date, and dosage and administration
- Drugs used other than study drugs
- Blood pressure
- Head MRI (required)

Date of scan (between stroke and enrollment)
Clinical subtype: lacunar, atherothrombotic, or other
Responsible lesion: supratentorial, infratentorial, or both

- Head MRI T2* (optional)
  Date of scan (between stroke and enrollment)
  Microbleeds: No or Yes, (location and number)
- Head MRA (optional)
  Date of scan (between stroke and enrollment)
  At least 50% stenosis of a major intracranial artery (to the level of A2, M2, or P2): No or Yes
- Image evaluation of cervical arteries (optional)
  Laboratory procedures: CTA, MRA, ultrasound, DSA
  Date of scan (between stroke and first observation)
  At least 50% stenosis of an extracranial artery (the common carotid artery, internal carotid artery, vertebral artery, brachiocephalic artery, or subclavian artery): No or Yes
- Clinical laboratory tests
  Hematology: white blood cell count, red blood cell count, hemoglobin, hematocrit, and platelet count
  Clinical chemistry: AST, ALT, TC, TG, HDL-C, creatinine, fasting blood glucose, uric acid, and HbA1c (only for patients with diabetes mellitus)
  Urinalysis: Glucose, protein, and occult blood
- Chest X-rays Normal/Abnormal
- ECG Normal/Abnormal

### 6.3.2. All observations after first month

The following items will be observed or evaluated according to the observation schedule:

- Observation status: Ongoing or unavailable
- Date of observation (date of last information if unavailable for observation)
- Presence or absence of events
  If events have occurred, refer to *6.3.3. Events.*
- Presence or absence of adverse events or serious adverse events
  If events have occurred, refer to *23. Procedures for Adverse Events* and *24. Evaluation of Adverse Events*.
- Modified Rankin Scale (mRS)
- Status of study drug administration
  Administration rate: ≥70% or <70%
  Withdrawal (<4 weeks of continuous drug withdrawal): No or Yes (length)

Discontinuation (≥4 weeks of continuous discontinuation): No or Yes (date and reasons)

- Drugs used other than study drugs
- Blood pressure

#### 6.3.3. Events

If any event described below occurs, the information below must be reported.

Refer to *23. Procedures for Adverse Events* and *24. Evaluation of Adverse Events* for information that must be reported in case of serious adverse events.

If a subject is transported to another medical institution due to an event, the following information will be obtained through cooperation from the subject or family, etc., then entered into medical records and used as reference materials.

Death

Date, cause, and rationale for cause

Ischemic stroke (IS)

Date

Clinical subtype: lacunar, atherothrombotic, or other

Responsible lesion: supratentorial, infratentorial, or both

Intracerebral hemorrhage (ICH)

Date

Subarachnoid hemorrhage (SAH)

Date

TIA

Date

Myocardial infarction (MI)

Date

Other vascular events*

Date and details

* e.g. aortic dissection or rupture; pulmonary embolism; heart failure, angina pectoris, or occlusive arteriosclerosis requiring hospitalization; and revascularization of coronary artery, aorta, peripheral artery, etc.

# 7. Adverse Events

## 7.1. Definition

### 7.1.1. Adverse events

Adverse events are any medically undesirable event observed in a subject who has received a study drug, even if the causal relationship is not known.

In other words, adverse events are any undesirable or unintended sign (including abnormal laboratory values),

symptom, or illness observed under treatment with a study drug, regardless of causal relationship with the treatment.

Aggravation of any symptom, sign, or disease preexisting granting of consent or start of observation, will be handled as a new adverse event.

#### 7.1.2. Serious adverse events

Serious adverse events are adverse events for which any of the following apply:

1) Fatal
2) Life-threatening

   The term "life-threatening" refers to an event in which the subject was at risk of death at the time of the adverse event; it does not refer to an event which hypothetically might have caused death if it were more severe.
3) Requires hospitalization or prolongation of existing hospitalization for treatment
4) Results in persistent or significant disability/incapacity
5) Is a congenital anomaly/birth defect
6) Other conditions, i.e., a medically important event for which treatment is required to avoid risks or items 1)–5) above, even if the event is not immediately life-threatening, or resulting in death or hospitalization. Examples include bronchospasm requiring intensive care in emergency rooms etc.; blood disorder or convulsions not to leading to hospitalization; and drug dependence or abuse.

Explanation of "hospitalization for treatment" for serious adverse events

"Hospitalization for treatment" is defined as hospitalization of a subject in a medical institution (usually for at least one night) for treatment of adverse events, even if no particular treatment is performed (resting cure). This does not include hospitalization for examination or treatment of the primary disease or complications not aggravated from pre-observation conditions; hospitalization for convenience or personal reasons not intended to treat adverse events; and hospitalization for treatment or examination that was already scheduled prior to the first visit.

### 7.2. Procedures for adverse events

Refer to *23. Procedures for Adverse Events.*

### 7.3. Evaluation of adverse events

Refer to *24. Evaluation of Adverse Events.*

### 7.4. Follow-up of adverse events

Refer to *25. Follow-Up Investigation of Adverse Events*

### 7.5. Expected adverse drug reactions

Refer to the package insert of study drugs for expected adverse drug reactions and precautions.

### 7.6. Procedures for subjects who are suspected or known to be pregnant

Patients of childbearing potential are excluded from the study. However, an investigator/subinvestigator will conduct pregnancy tests during the observation period for subjects suspected to be pregnant. If the result is positive, the subject must discontinue study participation. Otsuka Pharmaceutical co., Ltd. must be informed of the matter if the pregnant subject was in the DAPT group. If the subject withdraws from the study, tests performed at discontinuation will be performed to the extent that the pregnancy is not adversely affected. Furthermore, follow-up investigation of the subject will continue until birth or termination of the pregnancy. For subjects in the DAPT group, follow-up results must be reported to Otsuka Pharmaceutical co., Ltd..

## 8. Efficacy Evaluation

### 8.1. Primary endpoint

The following is the study's primary endpoint:

- Recurrence of symptomatic ischemic stroke (with symptoms lasting for at least 24 hours)

### 8.2. Secondary endpoints

The following items are secondary endpoints:

- Any stroke [ischemic stroke (IS), intracerebral hemorrhage (ICH), or subarachnoid hemorrhage (SAH)]
- ICH or SAH
- Ischemic cerebrovascular disorder [IS or transient ischemic attack (TIA)]
- Death from any cause
- Stroke [IS, ICH, SAH], myocardial infarction (MI), or vascular death
- All vascular events: stroke, MI, and other vascular events [e.g. aortic dissection or rupture; pulmonary embolism; heart failure, angina pectoris, or occlusive arteriosclerosis requiring hospitalization; and revascularization of coronary artery, aorta, peripheral artery, etc.]

## 9. Safety Evaluation

The following are safety endpoints:

- Adverse events and adverse drug reactions
- Severe or life-threatening hemorrhage (GUSTO criteria)

## 10. Target Sample Size and Study Period

### 10.1. Target sample size

4,000 (2,000 per group)

### 10.2. Study period

The subject enrollment period lasts until target sample size is met. This period is planned to last from October 2013 to March 2017.

Study period lasts until 1 year after the first observation day of the last subject enrolled. This period is planned to last from October 2013 to March 2018.

## 11. Statistical Analysis

### 11.1. Rationale for target sample size

We assumed an annual recurrence rate of 4% in the monotherapy group based on data from the EVEREST study[26] and the aspirin monotherapy group in the JASAP study[8]. We also assumed a 30% decrease in relative recurrence risk (in other words, an annual relapse rate of 2.8%) in the DAPT group with cilostazol. The number of subjects required was calculated at 1,688 per group using the methods described by Lakatos et al[27] in our main analytical method of the log-rank test (conditions were assumed relapse rates for each group, a 2.5 year enrollment period, a maximum 3.5 year observation period, $\alpha$=0.05, and a statistical power of 80%). This results in a total target sample size of 4,000 (2,000 per group), assuming an annual dropout rate of 5% during the study period.

### 11.2. Analysis populations

The intent-to-treat (ITT) analysis population used for evaluating safety and efficacy is defined as all randomly allocated study subjects. In compliance with the CONSORT Statement, we will create a flow diagram containing numbers of enrolled subjects, randomly allocated subjects, subjects observed for 1 year, subjects observed for 2 years, etc.

### 11.3. Analysis items and methods

Demographic characteristics of patient backgrounds will be calculated for each group in the ITT analysis population. Continuous variables are shown as mean (standard deviation) or median (quartile ranges), while category variables are shown as number of subjects (percentage) per category. Mean and median values will also be calculated for length of subject observation periods. Intergroup bias will also be studied with appropriate tests based on variable characteristics.

Kaplan-Meier plots will be drawn by group for the primary endpoint of ischemic stroke recurrence, while groups will be compared with the log-rank test. Also, the Cox proportional hazards model will be used to calculate hazard ratios and 95% CIs for DAPT groups versus SAPT groups. Unadjusted hazard ratios and adjusted hazard ratios [adjusted according to age, sex, clinical type of cerebral infarction (atheroma or lacunar), and mRS] will be calculated at this time.

Annual recurrence rates will be calculated with both person-year and Kaplan-Meier methods, 95% CIs for which will be calculated with the approximate Poisson and Greenwood methods.

Subgroup analysis will include the following factors: age, sex, antiplatelet therapy (aspirin or clopidogrel), clinical subtype of ischemic stroke (atheroma or lacunar), presence or absence of 50% or greater stenosis of

extracranial arteries, mRS, medical history and complications (hypertension, diabetes mellitus, dyslipidemia, coronary heart disease, peripheral artery disease, chronic kidney disease, and ischemic stroke), smoking status, and BMI. Continuous variables will be divided at the median, at which point a Cox proportional hazards model including a comparison group, predictive factors, and interactions will be studied. Stratified hazard ratios of the combination group compared to the SAPT group will be calculated according to predictive factors. Uniformity of interactions will also be studied. Significance level for all statistical tests is bilateral 5%.

Details of statistical analysis are described in the statistical analysis protocol authored by the Statistical Analysis Committee and the analysis officers in charge of the final analysis in the data center.

## 11.4. Interim review

### 11.4.1. Interim review methods

Because this study involves a long observation period, interim review will be performed in order to secure subject safety. An interim review of safety endpoints (serious adverse events, adverse drug events and hemorrhagic events), in principal, will be performed once per year. Besides the above reviews of safety endpoints, a review of efficacy and safety endpoints will also be performed once only during the study. The operation date, methods and others were described in “The Independent Data Monitoring Committee Procedure Manual” separately.

The interim review of safety and efficacy endpoints will be performed when 50% of information has been obtained (when 4,000 person-years of data have been collected, or specifically approximately when the 2.5 year enrollment period ends). The Haybittle-Peto ad-hoc method[28, 29] will be used for discontinuation criteria of primary endpoint. No specific discontinuation criteria will be established for safety endpoints, but the Independent Data Monitoring Committee (IDMC) will consider discontinuation or continuation based on incidence rates and make a recommendation to study investigators.

Timing of the interim review of safety and efficacy endpoints was determined based on planned enrollment period, observation period, and withdrawals/dropouts. Because the enrollment period for the study is 2.5 years and observation will end 1 year following the date of initiation of observation of the final subject to enroll, the observation period will last 3.5 years for the first subject to enroll and 1 year for the last subject to enroll. Assuming that subjects will enroll at a regular rate, mean observation period will last 2.25 years for 9,000 total person-years (4,000 × 2.25). However, given the possibility of withdrawals or dropouts, mean observation period is assumed to be 2 years, resulting in a likely total of 8,000 person-years (4,000 subjects × 2 years). Therefore, the review of safety and efficacy endpoints will occur at the mid-point up to 8,000 person-years, or when 4,000 person-years of data has been collected.

### 11.4.2. Interim analysis

As analysis, listing and tabulating safety endpoints (serious adverse events, adverse drug events and hemorrhagic events) by treatment will be performed for every safety interim review. As an interim analysis for the review of efficacy and safety endpoints, statistical analysis processing will be performed after listing

and tabulating endpoints by treatment. Details will be described in the interim analysis protocol, which will be drafted together by the Statistical Analysis Committee, the Independent Data Monitoring Committee, and the data center by the time of data lock for the first interim analysis.

The interim analysis will be performed by the analysis officers in charge of the interim analysis in the data center. Then the officers and member of IDMC bear the duty to maintain the confidentiality of any information in the review.

### 11.5. Final analysis

Final analysis will be performed by the data center after full data lock in accordance with the statistical analysis protocol. Results of this analysis will be compiled in the final analytical report and submitted to the principal investigator and each committee member. The statistical analysis protocol will be authored together by the Statistical Analysis Committee and the data center prior to full data lock.

## 12. Case Report Form Data Entry and Submission

Investigators and subinvestigators will fill out case report form (CRFs) after observing subjects based on the observation schedule. The current study uses an Electronic Data Capture (EDC) system through a special Internet website for CRFs.

Details are described in the *CRF Manual*.

## 13. Data Management

Data collected through the EDC system will be stored and managed at the study's data center. Management methods will adhere to the system's operational procedures.

## 14. Ethics

### 14.1. Rules and regulations to be observed

All staff involved with the study will adhere to the World Medical Association's *Declaration of Helsinki* (amended 10/2008) and the *Ethical Guidelines for Clinical Studies* (fully revised 07/31/2008), as well as the Pharmaceutical Affairs Law and other relevant laws and regulations. All staff will also observe this study protocol.

### 14.2. Subject benefits and drawbacks

The current study falls within the scope of treatment norms, including tests.

Using cilostazol in combination with antiplatelet agents, which are considered to offer insufficient prophylactic efficacy against ischemic stroke recurrence as monotherapy, may mitigate recurrence risk over the long term. This constitutes a benefit for subjects, who are able to receive a new method of treatment that had not been previously available. Observations of subjects scheduled at regular intervals during the study period also means that patients will be receiving regular treatment and diagnostic attention.

As described above in *2. Background and Rationale*, concomitant use of aspirin and cilostazol has not been

associated with an increase in hemorrhagic complications. Similarly, concomitant use of the clopidogrel and cilostazol did not result in increased hemorrhagic complications in overseas research on cardiovascular diseases. The same safety is expected with regard to ischemic strokes, but there are no detailed reports available at this time.

**14.3.** Review and discussion by ethics committee

According to *Ethical Guidelines for Clinical Studies*, study center directors must request the institution's ethics committee, either established by the director or otherwise, to make a preliminary review of study protocols to ascertain whether or not they adhere to the guidelines, as well as other issues related to appropriate performance of clinical studies. Items must be brought before the committee as necessary.

### 14.4. Authoring and revision of informed consent forms and written information for subjects

Investigators must author and revise as appropriate informational documents as well as consent forms in order to obtain consent from potential subjects. Authoring and revision of these documents must comply with the ethical principles of both the *Declaration of Helsinki* and the *Ethical Guidelines for Clinical Studies*.

In accordance with the *Declaration of Helsinki* and the *Ethical Guidelines for Clinical Studies*, informed consent forms and written information for subjects must contain the following items:

1) Explanation of the clinical study
2) Target diseases and general treatments
3) Study objectives
4) Explanation of the study drugs
5) Study methodologies
6) Expected advantages and disadvantages for subjects
7) Treatment methods if not participating in study
8) Precautions for study participation
9) Planned study period and number of subjects
10) Study participation and withdrawal
11) Study discontinuation and responses
12) Offering of information related to study
13) Protection of personal information
14) Storage, usage methods, and storage period of materials
15) Response and compensation if subject health is damaged
16) Conflicts of interest and expenses shouldered by subjects
17) Study personnel contacts, study doctors, and contact information

### 14.5. Informed consent

Prior to any subject participation in study, an investigator/subinvestigator will make a full explanation with informational materials and obtain freely-given consent in writing from the subject himself or herself. Once

the potential subject fully understands the informational materials, both the investigator/subinvestigator who performed the explanation as well as the patient will sign (or print and affix personal seal) and date the consent documents. Next, the investigator/subinvestigator will give a copy of the signed (or printed with seal affixed) consent documents and informational materials to the subject, and store the original document at the study center.

Patients may not participate in this study if it is determined they lack the capacity to make their own judgments. However, if for some reason the patient is incapable of signing forms themselves, an alternate method of identification such as a fingerprint may be used to allow participation.

If significant changes are made to the informational materials, the investigator/subinvestigator will explain their content again to subjects using the updated documents, and obtain freely-given consent in writing for continued participation in the study.

### 14.6. Privacy protections and subject identification

All study personnel will strictly protect subject personal information in accordance with the Act on the Protection of Personal Information.

Investigators/subinvestigators will apply a uniformly-determined subject identification code when enrolling patients or when creating CRFs through the EDC system. No personal information which could identify the subject (name, initials, address, telephone number, etc.) will be used. Investigators/subinvestigators will create a chart linking subject identification codes to chart numbers and other personal information of subjects (a subject identification code list), and store this at the study center. Subject identification codes will be of the following format: **study center code (3-digit)—sequential number (3-digit)**. Study center codes will be allocated to each study center by the data center. Subject identification codes will be used to identify subjects when the data center makes inquires to study centers about data.

## 15. Study Funding and Conflicts of Interest

This study is performed under contract between the Japan Cardiovascular Research Foundation and Otsuka Pharmaceutical co., Ltd.. In accordance with rules regarding funding transparency and conflicts of interest of related study institutions, academic societies where results may be presented, publishers, and other entities, the Japan Cardiovascular Research Foundation and Otsuka Pharmaceutical co., Ltd. publically disclose the fact that Otsuka Pharmaceutical co., Ltd. is providing funding for this study.

## 16. Study Expenses

All treatment performed within the scope of the current study is covered by the national health insurance system. No drugs or tests will be used which are not covered by national health insurance. Further, the number of hospital visits and frequency of tests are similar to those under normal treatment, so participating in the current study constitutes no financial burden for subjects.

# 17. Responses and Compensation for Damage to Subject Health

## 17.1. Responses to damage to health

The tests and treatments in the study protocol all lie within the scope of normal treatment. Therefore, in the event of any damage to subject health, investigators/subinvestigators will take all appropriate actions, including full treatment, the expenses for which will be fundamentally compensated by insurance as normal medical procedures. In the event of serious damage to health, subjects will receive an explanation of procedures for applying for compensation from The Relief System for Sufferers from Adverse Drug Reactions.

## 17.2. Clinical study insurance

Otsuka Pharmaceutical co., Ltd. will enroll in clinical study insurance for the entire study organization in order to cover indemnity liability as well as compensatory liability.

# 18. Revision of the Study Protocol

If revision of the study protocol becomes necessary after initiation of the study, the principal investigator will perform revisions after gaining approval from the Protocol Authoring Committee. If revisions are deemed significant, they must first be reviewed by the ethics committee of the principal investigator's institution.

After revision, the principal investigator will send the revised protocol or details of the revisions to investigators, all committee members, and the data center. Procedures for review of changes at each study center will follow regulations of applicable ethics committees. Finally, investigators at each study center will revise informed consent forms and written information for subjects according to the changes in the study protocol.

# 19. Study Completion and Early Discontinuation

## 19.1. Study completion at each study center

When the study concludes at a study center, the investigator will submit a report of study completion to the center director without delay. The investigator will also submit a copy of the report of study completion to the principal investigator.

## 19.2. Study cancellation or discontinuation at each study center

1) Investigators will assess the issue of whether or not to continue the study in any of the following circumstances:

   iii. Significant information is obtained about study drug quality, safety, or efficacy

   iv. A determination has been made that study continuation is problematic

2) Investigators will cancel or discontinue the study in any of the following circumstances:

   iii The ethics committee issues an order or recommendation to cancel or discontinue the study

   iv The ethics committee issues problematic orders for changing the study protocol etc.

3) Investigators will inform the principal investigator of any decision made to cancel or discontinue the study as

soon as possible in writing, along with reasons for this decision.

4) Investigators will inform subjects of any decision made to cancel or discontinue the study, and arrange for appropriate treatment and post-processing.

### 19.3. Overall completion of study

Study is complete once all CRFs from all study centers are confirmed. Once the principal investigator receives notification from the data center that all CRFs have been confirmed, he or she will inform investigators and all committee members at each study center that the study has been concluded. Investigators will then notify the director of their institutions and related departments of this in writing.

### 19.4. Early discontinuation of study

The principal investigator will act accordingly if he or she receives a recommendation from the Independent Data Monitoring Committee to discontinue the study early. If the principal investigator determines not to follow this recommendation, he or she will inform the Independent Data Monitoring Committee of this fact together with the basis for the decision, and discuss the issue.

If the principal investigator determines to follow the recommendation to discontinue the study early, he or she will immediately contact all investigators, committee members, and the data center to inform them of the early discontinuation, reasons for it, and follow-up responses. After being informed of the early study discontinuation, investigators will convey this to subjects along with the reasons for it, and perform appropriate alternate treatments. Finally, investigators will then notify the director of their institutions and related departments of the early discontinuation in writing.

## 20. Storage of Study-Related Materials

Investigators will store all documents related to the current clinical study (copies of applications and reports, notifications from study center directors, subject identification code lists, consent documents, CRF copies, and other documents or records necessary for ensuring reliability of data) either until discontinuation or the date 5 years following completion, then dispose of the records in a manner that does not compromise personal information.

## 21. Clinical Study Registration

This clinical study will be registered with UMIN-CTR and ClinicalTrials.gov prior to initiation.

## 22. Authoring of Clinical Study Report, Disclosure of Study Results, and Ownership of Results and Intellectual Property

The principal investigator and the Steering Committee will summarize the contents of the final analysis report and author a clinical study report encompassing overall conclusions from the study, problematic issues, analysis and discussion of results, and plans going forward from a primarily clinical perspective.

Study results will also be reported as a paper, with authors to be determined together by the principal

investigator and the Steering Committee.

All data and intellectual property (patent rights, utility model rights, and inventions or devices utilizing these rights) generated by the study will be the property of Otsuka Pharmaceutical co., Ltd..

# 23. Procedures for Adverse Events

## 23.1. Subject-related response

The investigator/subinvestigator give sufficient medical response to all clinically problematic adverse events related to the study both during and after the subject's study participation. The subject should be informed when treatment for adverse events is required.

## 23.2. Serious adverse event reporting

### 23.2.1. Serious adverse events (SAE) requiring immediate reporting

The following SAEs require immediate reporting:

1) All SAEs occurring during the observation period, regardless of their relationship with the study drug.
2) SAEs for which causal relationship with study drugs cannot be denied occurring during the course of a follow-up investigation of an adverse event (see *25. Follow-Up Investigation of Adverse Events*), or an SAE which becomes serious during a follow-up investigation and for which causal relationship with study drugs cannot be denied.
3) SAEs occurring after completion of the study period reported to the investigator/subinvestigator by the subject, and for which the causal relationship to the study drugs cannot be denied according to the investigator/subinvestigator.

### 23.2.2. Immediate reporting procedures

Reporting will adhere to the following procedures:

1) When adverse events corresponding to the previous section occur, the investigator/subinvestigator will immediately input required information into the EDC (CRF) system, which will report relevant information to the study sponsor and Otsuka Pharmaceutical co., Ltd..
2) The investigator/subinvestigator will then submit a detailed report about the SAE (onset of which occurred following study drug treatment) to the study center director as soon as possible, using forms required by the institution. The same information will be input into the EDC (CRF) system, which will report relevant information to the study sponsor. Furthermore, the information of DAPT group will be reported to PV department (pharmacovigilance division) in Otsuka Pharmaceutical co., Ltd..
3) The investigator/subinvestigator will furnish additional information about the reported SAE (including autopsy reports, terminal stage medical records, or any other information) to the study sponsor, study center director, ethics committee, and Otsuka Pharmaceutical co., Ltd. as requested.

### 23.2.3. Reporting of additional information following immediate reporting

When new information (e.g. course, test results, procedures, etc.) is obtained about an SAE for which an

immediate report has already been made, this information will be input into the EDC system (CRF).

#### 23.2.4. Handling of unexpected SAEs

Unexpected SAEs are events which are judged to be adverse drug reaction (ADRs) due to the study drug, but which are not described in the package insert information for that drug; or, if they are described in the package insert, they differ in quality or severity from that description. These events are handled as follows:

1) **If due to cilostazol**

Otsuka Pharmaceutical co., Ltd. will report unexpected SAEs (ADRs) reported according to *23.2.2. Immediate reporting procedures* to the Minister of Health, Labor and Welfare or a party under contract to said Minister.

2) **If due to other drugs**

The director of each study center will report to the Minister of Health, Labor and Welfare or a party under contract to said Minister in accordance with Article 77-4-2 of Pharmaceutical Affairs Law and *Ethical Guidelines for Clinical Studies.*

#### 23.2.5. SAE reporting to IDMC and other collaborating clinical study institutions for the study

The analysis officers in charge of the interim analysis in the data center will make lists and tabulate SAEs reported according to *23.2.2. Immediate reporting procedures*, then report to IDMC members every month. Details were described in “The Independent Data Monitoring Committee Procedure Manual”. Instead of SAEs reporting to investigators at other site as prescribed by the *Ethical Guidelines for Clinical Studies*, the analysis officer will report the listed SAEs to all investigators.

These reports will not include information on treatment group in order to avoid various biases.

## 24. Evaluation of Adverse Events

The investigator/subinvestigator will evaluate adverse events in accordance with the following:

### 24.1. Event name

Name of disease or condition diagnosed will be used for each event if known instead of individual symptoms.

### 24.2. Event date

Event date is the day of occurrence or confirmation of the adverse event. If diseases, symptoms, or signs existing as when consent was granted become aggravated during the observation period, the day of aggravation is considered the day of adverse event occurrence.

### 24.3. Severity

Severity of adverse events is classified into 3 levels as follows:

1. Mild: Causing discomfort but no interference with daily activities
2. Moderate: Causing discomfort sufficient to restrict or affect daily activities

3. Severe: Cannot perform work or daily activities

## 24.4. Seriousness

Seriousness of adverse events is classified into 2 levels as follows in accordance with *7.1.2. Serious adverse events*.

1. Serious
2. Not serious

## 24.5. Causal relationship with study drugs

Causal relationship with study drugs is classified according to the following 2 categories:

1. Relationship cannot be denied
4. Relationship can be denied

If an investigational drug or concomitant drug is judged responsible, the responsible drug should be identified.

[Reference for determining causal relationship with study drugs]

1. Relationship cannot be denied

Cases where any of the following reasons make a causal relationship a logical possibility:

a If it the event is suggested by package insert

b If there is a suspected temporal relationship between study drug administration and event occurrence (e.g., development of allergic dermatitis several days after study drug administration)

c If there is a suspected relationship between study drug discontinuation or dosage reduction and event outcome (e.g., rapid recovery of nausea following study drug discontinuation)

d Other cases in which study drug involvement cannot be medically ruled out

4. Relationship can be denied

Cases where any of the following reasons make a causal relationship logically improbable:

a If the event may be due to the primary disease, complications, or medical history

b If the event may be due to age, sex, or other factors

c If there is little chance of temporal relationship between study drug administration and event (e.g., adverse events occurring a significant period of time after completion of study drug administration)

d If there is little chance of relationship due to observed course of event [e.g., subject recovers from event while continuing study drug administration and with no other actions taken (although possibility of habituation to study drug must be taken into account)]

e If effects of concomitant drug are considered likely

f If the event is considered likely to be due to chance alone (accident, spontaneous

symptoms, etc.) (e.g., femur fracture due to being caught in a traffic accident)

g Other cases in which study drug involvement can be medically ruled out

### 24.6. Study drug-related actions

The following study drug-related actions are available as responses to occurrence of adverse events:

1. Drug withdrawal
2. Discontinuation
3. Dose reduction
4. Dose increase
5. No change
99. Unknown

### 24.7. Adverse event-related actions

Existence of therapy (a drug therapy or other treatment) and contents of the therapy conducted for developed adverse events should be recorded.

### 24.8. Outcome

Select one of the following 6 categories for adverse event outcomes. Record the date of death if the subject died, and the date of confirmation for relief (in process of recovery), not recovered, or unknown.

1. Recovery
2. Relief (in process of recovery)
3. Recovery with sequelae
4. Not recovered
5. Fatal outcome
99. Unknown (if follow-up investigation impossible for some reason, etc.)

## 25. Follow-Up Investigation of Adverse Events

Follow-up investigation of adverse events will be performed according to the following procedures.

"Recovery" refers to a subject returning to original state of health following an adverse event. For adverse events consisting of aggravation of an existing disease, symptom, or sign during the observation period that was present when consent was granted, "recovery" refers to returning to the health state as of the first day of observation.

1) If the subject has not recovered from an adverse event as of the day of completion of observation or the day of discontinuation, the investigator/subinvestigator will explain follow-up investigations and their purpose to the subject, request cooperation, and perform the investigation within 4 weeks (the day the follow-up investigation is performed is recorded as the follow-up investigation day).
2) If there is no recovery of the adverse event as of the day of the follow-up investigation and a causal relationship between the study drug and event cannot be denied, the investigation should be

continued when possible until recovery or stabilization of the adverse event. If a causal relationship between the study drug and event can be denied, the follow-up investigation will conclude on the day the follow-up investigation is performed.

3) If a new SAE for which a causal relationship with the study drug cannot be denied is observed after the day of completion of observation (or the day of discontinuation) and before the follow-up investigation day; or if an adverse event for which a causal relationship with the study drug cannot be denied that has not been recovered from as of the day of completion of observation (or the day of discontinuation) becomes an SAE; then the investigation should be continued until recovery or stabilization of the adverse event.
4) If an SAE for which a causal relationship with the study drug cannot be denied is discovered after the day of completion of observation (or the day of discontinuation) or after the follow-up investigation day, it will not be investigated in the current study. Therefore, it should be reported according to Article 253 of Enforcement Regulations of the Pharmaceutical Affairs Law, but not through the EDC system.

## 26. Drug Information

Summaries of all study drugs are shown below. Refer to package inserts of each for details.

### 26.1. Aspirin

Aspirin inhibits the cyclooxygenase 1 (COX-1) enzyme, which in turn inhibits synthesis of the thromboxane A2 (TXA2) enzyme, thereby inducing platelet aggregation inhibition. COX-1 inhibitory action in platelets is irreversible because platelets cannot resynthesize the enzyme. However, COX-1 is re-synthesized in blood vessel tissue, so inhibitory effects on synthesis of prostacyclin (PGI2) are reversible and exhibit relatively rapid recovery. In addition, the metabolite salicylic acid does not inhibit COX-1 and does not display platelet aggregation inhibition.

### 26.2. Clopidogrel

The active metabolite of clopidogrel sulfate irreversibly acts on the P2Y12 subtype of platelet ADP receptors, and suppresses platelet aggregation and therefore platelet activity by inhibiting ADP binding.

### 26.3. Cilostazol

Cilostazol exhibits antithrombogenic action by specifically inhibiting phosphodiesterase 3 (PDE3), an enzyme which metabolizes cAMP, thereby inhibiting platelet aggregation. The drug also acts as a vasodilator, and has been reported to increase cerebral blood flow in ischemic stroke patients and blood flow to lower extremities in patients with chronic arterial obstruction.

## 27. Committees

The following types of committees will be established, members of which are described in *28. Study*

*Organization* below.

### 27.1. Steering Committee

This committee is to receive advice from the principal investigator and discuss overall operation of the study while also facilitating the study.

### 27.2. Protocol Authoring Committee

This committee is to receive advice from the principal investigator and members of the Independent Data Monitoring Committee and perform revisions to the study protocol.

### 27.3. Independent Data Monitoring Committee

This committee is to give advice on continuation or discontinuation of the study, as well as revision of the protocol and other issues, based on study progress, serious adverse events, event incidences, results of interim analysis, and other factors. In principal, IDMC will perform an interim review based on results of interim analysis with safety information and other data once per year. Beside monthly safety monitoring and yearly safety interim review, the committee will also perform an efficacy and safety interim review once at the mid-point of the study based on interim analysis of inter-group comparisons. Details were described in “The Independent Data Monitoring Committee Procedure Manual”.

### 27.4. Event Evaluation Committee

This committee is to evaluate validity of subject-related data after an event (primary or secondary endpoint) occurs.

### 27.5. Statistical Analysis Committee

This committee is to suggest and supervise methodologies for group allocation and statistical analysis in the study protocol (establish for target sample size and their rationales, analytical methods, etc.). The committee will also cooperate with the Independent Data Monitoring Committee and the data center in authoring an interim analysis protocol prior to the first interim review. Additionally, the committee will also cooperate with the Independent Data Monitoring Committee and the data center in authoring an analysis protocol prior to the data locked. The committee will also oversee statistics-related materials.

## 28. Study Organization

### 28.1. Study sponsor

Takenori Yamaguchi, President,
Public interest incorporated foundation, Japan Cardiovascular Research Foundation

### 28.2. Principal Investigator

Takenori Yamaguchi, President emeritus, National Cerebral and Cardiovascular Center

**28.3. Steering Committee**

(in alphabetical order)

Shinichirou Uchiyama, Professor, Department of Neurology, Tokyo Women's Medical University (Chairman)

Yasushi Okada, Director, Clinical Research Institute, National Hospital Organization Kyushu Medical Center

Kazumi Kimura, Professor, Department of Stroke Medicine, Kawasaki Medical School

Nobuyuki Sakai, Director, Department of Neurosurgery, Kobe City Medical Center General Hospital

Kotaro Tanaka, Professor, Department of Neurology, University of Toyama

Kazunori Toyoda, Director, Department of Cerebrovascular Medicine, National Cerebral and Cardiovascular Center

Haruhiko Hoshino, Director, Department of Neurology and Stroke Center, Tokyo Saiseikai Central Hospital

**28.4. Protocol Authoring Committee**

(in alphabetical order)

Kazumi Kimura, Professor, Department of Stroke Medicine, Kawasaki Medical School

Kazunori Toyoda, Director, Department of Cerebrovascular Medicine, National Cerebral and Cardiovascular Center (Chairman)

Haruhiko Hoshino, Director, Department of Neurology and Stroke Center, Tokyo Saiseikai Central Hospital

**28.5. Independent Data Monitoring Committee**

(in alphabetical order)

Tatsuya Isomura, Chief executive, CLINICAL STUDY SUPPORT, Inc.

Shinya Goto  Professor, Cardiovascular Medicine, Tokai University School of Medicine

Hiroaki Naritomi**,** Director, Senri Chuo Hospital (Chairman)

**28.6. Event Evaluation Committee**

(in alphabetical order)

Yasuo Terayama, Professor, Department of Neurology, Iwate Medical University

Teiji Tominaga, Professor, Department of Neurosurgery, Tohoku University

Hidekazu Tomimoto, Professor, Department of Neurology, Mie University

Kiyohiro Houkin, Professor, Department of Neurosurgery, Hokkaido University

Masayasu Matsumoto, Professor, Department of Neurology, Hiroshima University

Kazuo Minematsu, Deputy Director General, National Cerebral and Cardiovascular Center Hospital (Chairman)

Satoshi Yasuda, Director, Department of Cardiovascular Medicine, National Cerebral and Cardiovascular Center

### 28.7. Statistical Analysis Committee

Hideki Orikasa, Professor, Biostatistics and Clinical Epidemiology, University of Toyama Graduate School of Medicine (Chairman)

Naoko Kumagai, Associate Professor, Integrated Center for Advanced Medical Technologies, Kochi Medical School Hospital

### 28.8. Study Secretariat

CSPS.com Study office, Public interest incorporated foundation, Japan Cardiovascular Research Foundation
11F, Nissei Shin-osaka Bldg. 3-4-30 Miyahara, Yodogawa-ku, Osaka 532-0003, Japan
Clinical Coordinating Center, EPS Corporation
TEL: 0120-05-3125 / FAX: 06-4807-3025
E-mail: prj-csps.cont.com@eps.co.jp

### 28.9. Data Center

Data management; Clinical Information Division Data Management Center 1, EPS Corporation
Nissei Shin-osaka Bldg., 3-4-30 Miyahara, Yodogawa-ku, Osaka 532-0003, Japan
TEL: 0120-80-0226 / FAX: 06-4807-1117
E-mail: prj-csps-com-etr@e-trial.co.jp

Statistical analysis; Clinical Information Division Data Science Center, Statistics Analysis Department 1, EPS Corporation
Kagurazaka AK Bldg., 1-8 Tsukudo-cho, Shinjuku-ku, Tokyo 162-0821, Japan
TEL: 03-5684-7799 / FAX: 03-5684-7798

### 28.10. Study Centers

Listed separately.

## 29. References

### 29.1. Literature quoted

1) Chimowitz MI, Lynn MJ, Howlett-Smith H, et al. Comparison of warfarin and aspirin for symptomatic intracranial arterial stenosis. *N Engl J Med* 2005; 352: 1305-16.
2) Scott EK, Marc IC, Michael JL, et al. Predictors of Ischemic Stroke in the Territory of a Symptomatic Intracranial Arterial Stenosis. *Circulation* 2006; 113: 555-63.
3) Andrew NN, Stavros KK, Efthyvoulos K, et al. Asymptomatic internal carotid artery stenosis and cerebrovascular risk stratification. *J Vasc Surg* 2010; 52: 1486-96.
4) Kamouchi M, Kumagai N, Okada Y, Origasa H, Yamaguchi T, Kitazono T. Risk score for predicting recurrence in patients with ischemic stroke: the Fukuoka stroke risk score for Japanese. *Cerebrovasc Dis* 2012; 34: 351-7.

5) Diener HC, Ringleb PA, Savi P. Clopidogrel for the secondary prevention of stroke. *Expert Opin Pharmacother* 2005; 6: 755-64.
6) Diener HC, Cunha L, Forbes C, Sivenius J, Smets P, Lowenthal A. European Stroke Prevention Study. 2. Dipyridamole and acetylsalicylic acid in the secondary prevention of stroke. *J Neurol Sci* 1996; 143: 1-13.
7) The ESPRIT Study Group. Aspirin plus dipyridamole versus aspirin alone after cerebral ischaemia of arterial origin (ESPRIT): randomised controlled trial. *Lancet* 2006; 367: 1665-73.
8) Uchiyama S, Ikeda Y, Urano Y, Horie Y, Yamaguchi T. The Japanese aggrenox (extended-release dipyridamole plus aspirin) stroke prevention versus aspirin programme (JASAP) study: a randomized, double-blind, controlled trial. *Cerebrovasc Dis* 2011; 31: 601-13.
9) Markus HS, Droste DW, Kaps M, et al. Dual antiplatelet therapy with clopidogrel and aspirin in symptomatic carotid stenosis evaluated using doppler embolic signal detection: the Clopidogrel and Aspirin for Reduction of Emboli in Symptomatic Carotid Stenosis (CARESS) trial. *Circulation* 2005; 111: 2233-40.
10) FASTER Investigators. Fast assessment of stroke and transient ischaemic attack to prevent early recurrence (FASTER): a randomised controlled pilot trial. *Lancet Neurol* 2007; 6: 961-9.
11) Wong KS, Chen C, Fu J, et al. Clopidogrel plus aspirin versus aspirin alone for reducing embolisation in patients with acute symptomatic cerebral or carotid artery stenosis (CLAIR study): a randomised, open-label, blinded-endpoint trial. *Lancet Neurol* 2010; 9: 489-97.
12) Wang Y, Wang Y, Zhao X, et al. Clopidogrel with aspirin in acute minor stroke or transient ischemic attack. *N Engl J Med* 2013; 369: 11-9.
13) Diener HC, Bogousslavsky J, Brass LM, et al. Aspirin and clopidogrel compared with clopidogrel alone after recent ischaemic stroke or transient ischaemic attack in high-risk patients (MATCH): randomised, double-blind, placebo-controlled trial. *Lancet* 2004; 364: 331-7.
14) Bhatt DL, Fox KA, Hacke W, et al. Clopidogrel and aspirin versus aspirin alone for the prevention of atherothrombotic events. *N Engl J Med* 2006; 354: 1706-17.
15) The SPS 3 Investigators. Effects of clopidogrel added to aspirin in patients with recent lacunar stroke. *N Engl J Med* 2012; 367: 817-25.
16) Gotoh F, Tohgi H, Hirai S, et al. Cilostazol stroke prevention study: a placebo-controlled double-blind trial for secondary prevention of cerebral infarction. *J Stroke Cerebrovasc Dis* 2000; 9: 147-57.
17) Shinohara Y, Katayama Y, Uchiyama S, et al. Cilostazol for prevention of secondary stroke (CSPS 2): an aspirin-controlled, double-blind, randomised non-inferiority trial. *Lancet Neurol* 2010; 9: 959-68.
18) Kwon SU, Cho YJ, Koo JS, et al. Cilostazol prevents the progression of the symptomatic intracranial arterial stenosis: the multicenter double-blind placebo-controlled trial of cilostazol in symptomatic intracranial arterial stenosis. *Stroke* 2005; 36: 782-6.
19) Kwon SU, Hong KS, Kang DW, et al. Efficacy and safety of combination antiplatelet therapies in patients with symptomatic intracranial atherosclerotic stenosis. *Stroke* 2011; 42: 2883-90.
20) Uchiyama S et al. Final Results of Cilostazol-Aspirin THerapy Against Recurrent Stroke with Intracranial artery Stenosis (CATHARSIS). *38th International Stroke Conference*, Honolulu, USA, Feb.6-8, 2013.

21) Wilhite DB, Comerota AJ, Schmieder FA, Throm RC, Gaughan JP, Rao AK. Managing PAD with multiple platelet inhibitors: the effect of combination therapy on bleeding time. *J Vasc Surg* 2003; 38: 710-3.
22) Hiatt WR, Money SR, Brass EP. Long-term safety of cilostazol in patients with peripheral artery disease: the CASTLE study (Cilostazol: A Study in Long-term Effects). *J Vasc Surg* 2008; 47: 330-6.
23) Sakurai R, Koo BK, Kaneda H, Bonneau HN, Nagai R. Cilostazol added to aspirin and clopidogrel reduces revascularization without increases in major adverse events in patients with drug-eluting stents: A meta-analysis of randomized controlled trials. *Int J Cardiol* 2013; 167: 2250-8.
24) Toyoda K, Yasaka M, Uchiyama S, et al. Blood pressure levels and bleeding events during antithrombotic therapy: the Bleeding with Antithrombotic Therapy (BAT) Study. *Stroke* 2010; 41: 1440-4.
25) The SPS3 Study Group. Blood-pressure targets in patients with recent lacunar stroke: the SPS3 randomised trial. *Lancet* 2013; 382: 507-15.
26) Suzuki N, Sato M, Houkin K, et al.: One-year atherothrombotic vascular events rates in outpatients with recent non-cardioembolic ischemic stroke: the EVEREST (Effective Vascular Event REduction after STroke) registry. *J Stroke Cerebrovasc Dis* 2012; 21: 245-53.
27) Lakatos E, Lan KK. A comparison of sample size methods for the logrank statistic. *Stat Med* 1992; 11: 179-91.
28) Haybittle JL. Repeated assessment of results in clinical trials of cancer treatment. *Br J Radiol* 1971; 44: 793-7.
29) Peto R, Pike MC, Armitage P, et al. Design and analysis of randomized clinical trials requiring prolonged observation of each patient. I. Introduction and design. *Br J Cancer* 1976; 34: 585-612.

## 29.2. Heart diseases which may be source of emboli

High-risk sources of emboli according to classification in the TOAST study (Trial of Org 10172 in Acute Stroke Treatment) are as follows:

Mechanical prosthetic valve, mitral stenosis with atrial fibrillation, atrial fibrillation (other than lone atrial fibrillation), left atrial/atrial appendage thrombus, sick sinus syndrome, recent myocardial infarction (< 4 weeks), left ventricular thrombus, dilated cardiomyopathy, akinetic left ventricular segment, atrial myxoma, infective endocarditis

## 29.3. Definitions of serious hepatic impairment and serious renal impairment

Defined as follows:

i. Serious hepatic impairment: AST and ALT levels both ≥ 2.5 times upper limit of study center standard, or complications of hepatic cirrhosis or active hepatitis

ii. Serious renal impairment: Serum creatinine level ≥ 3 times upper limit of study center standard

## 29.4. Definition of medical history and complications

Defined as follows:

1) Coronary artery disease : Diagnosed as angina pectoris or myocardial infarction at a medical

institution

2) Peripheral arterial disease: Comorbid with symptomatic peripheral arterial disease, or ABI < 0.9

3) Hypertension: Systolic blood pressure ≥ 140 mmHg, diastolic blood pressure ≥ 90 mmHg at any examination in the past 3 months, or receiving antihypertensive medication as hypertension treatment

4) Diabetes mellitus: Satisfying any of the following conditions:
   - Fasting plasma glucose ≥ 126 mg/dl
   - Casual blood glucose level ≥ 200 mg/dl
   - 2-hour value in oral glucose tolerance test (75 g dose) of ≥ 200 mg/dl
   - HbA1c (NGSP) ≥ 6.5%
   - Receiving a hypoglycemic agent
   - Diagnosed as diabetes mellitus at a medical institution

5) Dyslipidaemia: Satisfying any of the following conditions:
   - LDL cholesterol level ≥ 140 mg/dl
   - HDL cholesterol level < 40 mg/dl
   - Triglyceride ≥ 150 mg/dl
   - Receiving a lipid-lowering agent

6) Chronic kidney disease: Any of the following conditions persisting for more than 3 months:
   - Clear findings suggesting renal disorder in urinalysis, imaging, pathological diagnosis, or physical findings (especially proteinuria)
   - Estimated glomerular filtration rate (eGFR) < 60 mL/min/1.73 $m^2$

### 29.5. Degree of independence in activities of daily living

Evaluate as one of following 7 levels according to Japanese Modified Rankin Scale (Japanese Guidelines for Management of Stroke 2009):

0. No symptoms at all
1. No significant disability despite symptoms: able to carry out all usual duties and activities
2. Slight disability: unable to carry out all previous activities, but able to look after own affairs without assistance
3. Moderate disability: requiring some help, but able to walk without assistance
4. Moderate to severe disability: unable to walk without assistance and unable to attend to own bodily needs without assistance
5. Severe disability: bedridden, incontinent and requiring constant nursing care and attention
6. Dead

### 29.6. GUSTO Criteria

(GUSTO: Global Utilization of Streptokinase and t-PA for Occluded Coronary Arteries Trial)

Defined as follows:

- Severe or life-threatening hemorrhage: Intracranial bleeding or bleeding that causes substantial

hemodynamic compromise requiring treatment

- Moderate bleeding: Bleeding which requires blood transfusion
- Minor bleeding: Other bleeding, neither requiring transfusion nor causing hemodynamic compromise

# CSPS.com
# Clinical Study Protocol Change
# Version 1.0 to 1.1

The first four columns of this table identify the location of the changes in version 1.1 of the protocol.

| Page | Chapter | Ver 1.0 (deletions strike-through) | Ver 1.1 (additions in bold) |
|---|---|---|---|
| Cover page | | | **Version 1.1: 17 October, 2013** |
| All other pages (Header) | | CSPS.com<br>Version 1.0: 15 September, 2013 | CSPS.com<br>Version 1.1: 17 October, 2013 |
| 2 | Endpoints | Recurrence of symptomatic ischemic stroke ("ischemic stroke" hereafter) | Recurrence of symptomatic ischemic stroke ("ischemic stroke" hereafter)**, with symptoms lasting for at least 24 hours** |
| Table of Contents | | Updated to reflect new page numbers and sections | |
| 12 | 3.2 | *Rationale for inclusion criteria*<br>5) The criteria were established to determine high risk of stroke recurrence. | 5) The **following** criteria were established to determine high risk of stroke recurrence:<br>**i. Based on the WASID trial sub-analysis report[2]**<br>**ii. Based on the ACSRS study sub-analysis report[3]**<br>**iii. Based on the Stroke Prevention Model[5], the Fukuoka Stroke Risk Score4 and the CAPRIE trial sub-analysis.** |
| 15 | 5.4 | ozagrel sodium, and ticlopidine | ozagrel sodium, ticlopidine, **and combined drugs containing aspirin or clopidogrel** |
| 22 | 8.1 | Recurrence of symptomatic ischemic stroke | Recurrence of symptomatic ischemic stroke **(with symptoms lasting for at least 24 hours)** |
| 36 | 28.3 | | **Kotaro Tanaka, Professor, Department of Neurology, University of Toyama** |
| 36 | 28.5 | ~~Kotaro Tanaka, Professor, Department of Neurology, University of Toyama (Chairman)~~ | Hiroaki Naritomi, Director, Senri Chuo Hospital (Chairman) |
| | | ~~2) Kakkos SK, Sabetai M, Tegos T, et al. Silent embolic infarcts on computed tomography brain scans and risk of ipsilateral hemispheric events in patients with asymptomatic internal carotid artery stenosis. J Vasc Surg 2009;49:902-9~~ | **2) Scott EK, Marc IC, Michael JL, et al. Predictors of Ischemic Stroke in the Territory of a Symptomatic Intracranial Arterial Stenosis. Circulation 2006; 113: 555-63.**<br>**3) Andrew NN, Stavros KK, Efthyvoulos K, et al. Asymptomatic internal carotid artery stenosis and cerebrovascular risk stratification. J Vasc Surg 2010; 52: 1486-96.**<br>* Reference numbers of 3 and after were shifted by one. |

# CSPS.com
# Clinical Study Protocol Change
# Version 1.1 to 1.2

The first four columns of this table identify the location of the changes in version 1.2 of the protocol.

| Page | Chapter | Ver 1.1 (deletions strike-through) | Ver 1.2 (additions in bold) |
|---|---|---|---|
| Cover page | | | **Version 1.2: 24 February, 2014** |
| All other pages (Header) | | CSPS.com<br>Version 1.1: 17 October, 2013 | CSPS.com<br>Version 1.2: 24 February, 2014 |
| Table of Contents | | Updated to reflect new page numbers and sections | |
| 15 | 5.4 | ozagrel sodium, ticlopidine, and combined drugs … | ozagrel sodium, ticlopidine, **plasugrel,** and combined drugs … |
| 19 | 6.3.1 | Microbleeds: No or Yes | Microbleeds: No or Yes**, (location and number)** |
| 24 | 11.3 | Details of statistical analysis are described in the statistical analysis protocol authored by the Statistical Analysis Committee and the data center. | Details of statistical analysis are described in the statistical analysis protocol authored by the Statistical Analysis Committee and **the analysis officers in charge of the final analysis in** the data center. |
| 24 | 11.4.1 | An interim review of safety endpoints (serious adverse events and hemorrhagic events), in principal, will be performed once per year. | An interim review of safety endpoints (serious adverse events, **adverse drug events** and hemorrhagic events), in principal, will be performed once per year. ..<br><br>**The operation date, methods and others were described in "The Independent Data Monitoring Committee Procedure Manual" separately.** |
| 25 | 11.4.2 | As analysis, listing and tabulating safety endpoints (serious adverse events and hemorrhagic events) by treatment will be performed for every safety interim review. As an interim analysis for the review of efficacy and safety endpoints, ~~data fixing will be performed by the Data Center and~~ statistical analysis processing will be performed **by IDMC** after listing and tabulating endpoints by treatment. | As analysis, listing and tabulating safety endpoints (serious adverse events, **adverse drug events** and hemorrhagic events) by treatment will be performed for every safety interim review. As an interim analysis for the review of efficacy and safety endpoints, statistical analysis processing will be performed after listing and tabulating endpoints by treatment…<br><br>**The interim analysis will be performed by the analysis officers in charge of the interim analysis in the data center. Then the officers and member of IDMC bear the duty to maintain the confidentiality of any information in the review.** |

| | | | |
|---|---|---|---|
| 30 | 23.2.2 | 2) The same information will be input into the EDC (CRF) system, which will report relevant information to the study sponsor ~~and Otsuka Pharmaceutical co., Ltd.~~. | 2) The same information will be input into the EDC (CRF) system, which will report relevant information to the study sponsor. **Furthermore, the information of DAPT group will be reported to PV department (pharmacovigilance division) in Otsuka Pharmaceutical co., Ltd..** |
| 31 | 23.2.5 (Title) | SAE reporting to other collaborating clinical study institutions for the study | SAE reporting to **IDMC and** other collaborating clinical study institutions for the study |
| 31 | 23.2.5 | Otsuka Pharmaceutical co., Ltd. will make lists and tabulate SAEs reported according to 23.2.2. Immediate reporting procedures, then report to IDMC members every month instead of SAEs reporting to investigators at other site as prescribed by the Ethical Guidelines for Clinical Studies, the analysis officer will report the listed SAEs to all investigators. | The analysis officers in charge of the interim analysis in the data center will make lists and tabulate SAEs reported according to 23.2.2. Immediate reporting procedures, then report to IDMC members every month. Details were described in “The Independent Data Monitoring Committee Procedure Manual”. Instead of SAEs reporting to investigators at other site as prescribed by the Ethical Guidelines for Clinical Studies, the analysis officer will report the listed SAEs to all investigators. |
| 35 | 27.3 | IDMC will perform an interim review based on results of interim analysis with safety information and other data once per year. Beside monthly safety monitoring and yearly safety interim review, the committee will also perform an efficacy and safety interim review once at the mid-point of the study based on interim analysis of inter-group comparisons. | In principal, IDMC will perform an interim review based on results of interim analysis with safety information and other data once per year. Beside monthly safety monitoring and yearly safety interim review, the committee will also perform an efficacy and safety interim review once at the mid-point of the study based on interim analysis of inter-group comparisons. **Details were described in “The Independent Data Monitoring Committee Procedure Manual”.** |
| 37 | 28.9 | Clinical Information Division Data Management Center 1, EPS Corporation | Data management; Clinical Information Division Data Management Center 1, EPS Corporation<br><br>**Statistical analysis; Clinical Information Division Data Science Center, Statistics Analysis Department 1,<br>EPS Corporation<br>Kagurazaka AK Bldg., 1-8<br>Tsukudo-cho, Shinjuku-ku, Tokyo 162-0821, Japan<br>TEL: 03-5684-7799 / FAX:03-5684-7798** |

# CSPS.com
# Clinical Study Protocol Change
# Version 1.2 to 1.3

The first four columns of this table identify the location of the changes in version 1.3 of the protocol.

| Page | Chapter | Ver 1.1 (deletions strike-through) | Ver 1.2 (additions in bold) |
|---|---|---|---|
| Cover page | | | **Version 1.3: 22 February, 2016** |
| All other pages (Header) | | CSPS.com<br>Version 1.2: 24 February, 2014 | CSPS.com<br>Version 1.3: 22 February, 2016 |
| 4 | Study period | October 2013 to March 2017 | October 2013 to March 2018 |
| 24 | 10.2 | The subject enrollment period lasts until target sample size is met. This period is planned to last from October 2013 to March 2016.<br>Study period lasts until 1 year after the first observation day of the last subject enrolled. This period is planned to last from October 2013 to March 2017. | The subject enrollment period lasts until target sample size is met. This period is planned to last from October 2013 to March 2017.<br>Study period lasts until 1 year after the first observation day of the last subject enrolled. This period is planned to last from October 2013 to March 2018. |

# Study of Antiplatelet Combination Therapy for Ischemic Stroke Patients with High Risk of Recurrence

CSPS.com

(Cilostazol Stroke Prevention Study. *Combination*)

**Statistical Analysis Plan**

Protocol No. 021-TADD-1300-1

Ver. 1.00, March 10, 2014

# TABLE OF CONTENTS

Abbreviations and definitions ........................................ 4
1. Preface ........................................ 5
2. Study objectives and endpoints ........................................ 5
  2.1 Study objectives ........................................ 5
  2.2 Efficacy evaluation ........................................ 5
    2.2.1 Primary endpoint ........................................ 5
    2.2.2 Secondary endpoints ........................................ 5
  2.3 Safety evaluation ........................................ 5
3. Study design ........................................ 6
4. Target sample size and rationale ........................................ 7
5. Analysis sets ........................................ 7
6. Data analysis considerations ........................................ 7
  6.1 Software ........................................ 7
  6.2 Data conversions and derivations ........................................ 7
    6.2.1 Age ........................................ 7
    6.2.2 Body Mass Index (BMI) ........................................ 7
    6.2.3 Duration of morbidity ........................................ 8
    6.2.4 Observation period (person-years) ........................................ 8
    6.2.5 Evaluated events ........................................ 8
  6.3 Handling of timepoints ........................................ 8
  6.4 Handling of missing data ........................................ 9
  6.5 Significance level and confidence coefficient ........................................ 9
  6.6 Multiple comparisons/multiplicity ........................................ 10
  6.7 Adjustment using covariates ........................................ 10
  6.8 Subgroup analysis ........................................ 10
  6.9 Handling of patients with study drug administration errors ........................................ 11
7. Disposition of patients ........................................ 11
8. Description of analysis sets ........................................ 12
  8.1 Demographic and other baseline characteristics ........................................ 12
  8.2 Compliance ........................................ 13
    8.2.1 Study drug compliance ........................................ 13
    8.2.2 Observation period ........................................ 13
    8.2.3 Compliance for drugs other than study drugs ........................................ 14
9. Efficacy evaluation ........................................ 14

9.1 Primary endpoint....14
9.2 Secondary endpoints....15
9.2.1 Stroke (ischemic stroke, intracerebral hemorrhage, subarachnoid hemorrhage)....15
9.2.2 Intracerebral hemorrhage or subarachnoid hemorrhage....16
9.2.3 Ischemic cerebrovascular disorder (ischemic stroke, TIA)....16
9.2.4 Death from any cause....16
9.2.5 Stroke (ischemic stroke, intracerebral hemorrhage, subarachnoid hemorrhage), myocardial infarction, or vascular death....16
9.2.6 All vascular events: stroke, myocardial infarction, and other vascular events [e.g., aortic dissection or rupture; pulmonary embolism; heart failure, angina pectoris, or occlusive arteriosclerosis requiring hospitalization; and revascularization (e.g., coronary artery, aorta, peripheral artery)]....17
10. Safety evaluation....17
10.1 Adverse events....17
10.2 Adverse drug reactions....17
10.3 Serious adverse events....18
10.4 Serious adverse drug reactions....18
10.5 Hemorrhagic adverse events....18
10.6 Blood pressure....18
10.7 Other safety endpoints....19
Degree of independence in activities of daily living (mRS)....19
11. References....19
12. Revision history....20
Approvals section .... エラー! ブックマークが定義されていません。

## ABBREVIATIONS AND DEFINITIONS

| | |
|---|---|
| Evaluated events: | Death, ischemic stroke, intracerebral hemorrhage, subarachnoid hemorrhage, transient ischemic attack (TIA), myocardial infarction, other vascular events (aortic dissection or rupture; pulmonary thrombosis; heart failure, angina pectoris, or occlusive arteriosclerosis requiring hospitalization), revascularization (e.g., coronary artery, aorta, peripheral artery) |
| Observation completion: | Observation is complete when a subject meets the criteria for discontinuation/completion or when 1 year has elapsed from the initiation of observation for last subject enrolled |
| Observation period: | The period from the start to completion of the observations. |
| Duration of morbidity: | Period from the most recent noncardioembolic ischemic stroke to the start of observations |
| Descriptive statistics: | Number of subjects, mean, standard deviation, minimum, median, maximum |
| GUSTO | Global Utilization of Streptokinase and t-PA for Occluded Coronary Arteries Trial |
| mRS | Modified Rankin Scale |

# 1. PREFACE

This statistical analysis plan describes methods of summary and analysis with the purpose of providing the data required to prepare the report for this clinical study. It was prepared based on study protocol version 1.2, dated 2014/02/24 (CSPS.com).

# 2. STUDY OBJECTIVES AND ENDPOINTS

## 2.1 Study objectives

To examine the efficacy and safety of dual antiplatelet therapy (DAPT) including cilostazol (Pletaal OD Tablet® & reg.) in comparison with single antiplatelet therapy (SAPT) excluding cilostazol for secondary prevention of ischemic stroke in high-risk patients for stroke

## 2.2 Efficacy evaluation

### 2.2.1 Primary endpoint

The following will be the primary endpoint.

- Recurrence of symptomatic* ischemic stroke ("ischemic stroke" hereafter), with symptoms lasting for at least 24 hours

    * Symptoms continue for at least 24 hr after ischemic stroke onset.

### 2.2.2 Secondary endpoints

The following are the secondary endpoints.

- Any stroke (ischemic stroke, intracerebral hemorrhage, subarachnoid hemorrhage)
- Intracerebral hemorrhage or subarachnoid hemorrhage
- Ischemic cerebrovascular disorder [ischemic stroke or transient ischemic attack (TIA)]
- Death from any cause
- Stroke (ischemic stroke, intracerebral hemorrhage, subarachnoid hemorrhage), myocardial infarction, or vascular death
- All vascular events: stroke, myocardial infarction, and other vascular events [e.g., aortic dissection or rupture; pulmonary embolism; heart failure, angina pectoris, or occlusive arteriosclerosis requiring hospitalization; and revascularization (e.g., coronary artery, aorta, peripheral artery)]

## 2.3 Safety evaluation

The following will be the safety endpoints.

- Adverse events and adverse drug reactions
- Severe or life-threatening hemorrhage (GUSTO criteria)

## 3. STUDY DESIGN

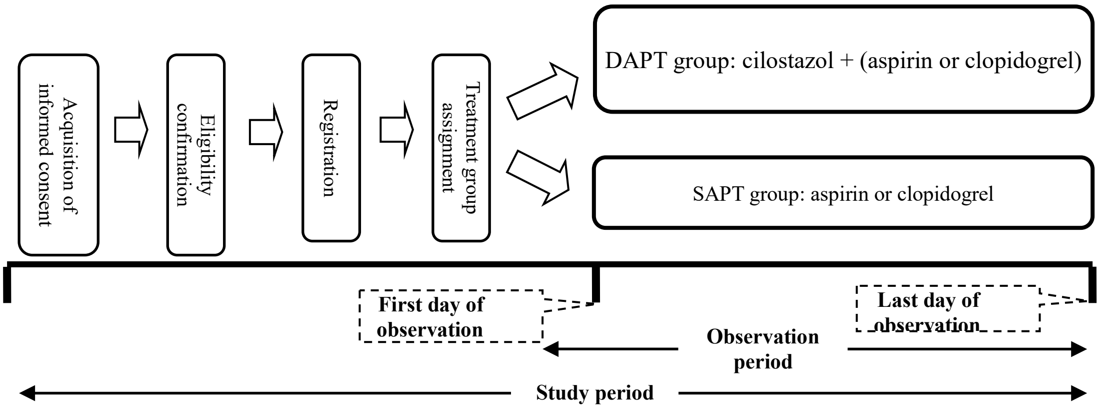


### Observation schedule

| Evaluation items \ Timepoint | Date of onset | Enroll-ment | Start of obser-vation | Month 1 | Month 3 | Month 6 | Month 12 | Every 6 months there-after | End of obser-Vation (*) |
|---|---|---|---|---|---|---|---|---|---|
| Informed consent | ← | ○ | | | | | | | |
| Enrollment | | ○ | | | | | | | |
| Observation status | | | | ○ | ○ | ○ | ○ | ○ | ○ |
| Patient characteristics | ← | ○ | | | | | | | |
| Degree of independence in activities of daily living (mRS) | | | ○ | ○ | ○ | ○ | ○ | ○ | ○ |
| Study drug administration/ compliance | | | ○ | ○ | ○ | ○ | ○ | ○ | ○ |
| Drugs other than study drug | | | ○ | ○ | ○ | ○ | ○ | ○ | ○ |
| Blood pressure | | | ○ | ○ | ○ | ○ | ○ | ○ | ○ |
| Head MRI | ← | ○ | | | | | | | |
| Head MRI (T2* WI) | ← | △ | → | | | | | | |
| Head MRA | ← | △ | → | | | | | | |
| Carotid artery imaging | ← | △ | → | | | | | | |
| Laboratory test (blood) | ← | ○ | → | ←⋯ | ⋯ | ⋯ | ⋯ | ⋯ | ⋯→ |
| Laboratory test (urine) | ← | ○ | → | ←⋯ | ⋯ | ⋯ | ⋯ | ⋯ | ⋯→ |
| Chest x-ray | ← | ○ | → | ←⋯ | ⋯ | ⋯ | ⋯ | ⋯ | ⋯→ |
| ECG | ← | ○ | → | ←⋯ | ⋯ | ⋯ | ⋯ | ⋯ | ⋯→ |
| Adverse events | | | ←— | — | — | — | — | — | —→ |

○ Required item △ Optional item ←⋯→ As required

* Observation is complete when a subject meets criteria for discontinuation/completion, or when 1 year has elapsed from initiation of observation for last subject enrolled.

# 4. TARGET SAMPLE SIZE AND RATIONALE

We assumed an annual recurrence rate of 4% in the monotherapy group based on data from the EVEREST study and the aspirin monotherapy group in the JASAP study. We also assumed a 30% decrease in relative recurrence risk (in other words, an annual recurrence rate of 2.8%) in the DAPT group with cilostazol. The number of subjects required was calculated at 1,688 per group using the methods described by Lakatos et al [3)] in our main analytical method of the log-rank test (conditions were assumed relapse rates for each group, a 2.5 year enrollment period, a maximum 3.5 year observation period, $\alpha$=0.05, and a statistical power of 80%). This results in a total target sample size of 4,000 (2,000 per group), assuming an annual dropout rate of 5% during the study period.

# 5. ANALYSIS SETS

The efficacy and safety analyses will use the intent-to-treat (ITT) analysis set.

ITT analysis set

The ITT analysis set is defined as the population of all randomized subjects. The subjects in this set belong to one of the treatment groups to which subjects were randomized, regardless of the drug they were actually administered.

The analysis set will be reexamined before database lock.

# 6. DATA ANALYSIS CONSIDERATIONS

## 6.1 Software

SAS release version 9.2 or later (SAS Institute, Cary, NC) will be used in the analysis.

## 6.2 Data conversions and derivations

### 6.2.1 Age

The subject's age at the time of informed consent (derived from date of birth and informed consent date indicated in database) will be used.

### 6.2.2 Body Mass Index (BMI)

BMI will be calculated based on the subject's height and weight at the time of the preliminary examination using the following formula.

BMI (kg/m$^2$) = weight (kg) / {height (m)}$^2$

#### 6.2.3 Duration of morbidity

The number of days from the most recent noncardioembolic ischemic stroke to the start of observations (first day of observations – day of ischemic stroke onset) will be calculated.

#### 6.2.4 Observation period (person-years)

For each group, the sum of the observation periods for the individual subjects will be determined, and the number of person-years will be calculated using 365.25 days for 1 year. The observation period (observation end date - observation start date) will be the number of days from the first observation day to the last observation day (day subject meets the criteria for discontinuation or completion, or 1 year from start of observations for final subject enrolled).

#### 6.2.5 Evaluated events

The number of days from the first day of observations until onset (date of evaluated event onset - observation start date) will be calculated for the following evaluated events.
Death, ischemic stroke, intracerebral hemorrhage, subarachnoid hemorrhage, transient ischemic attack (TIA), myocardial infarction, other vascular events [aortic dissection or rupture; pulmonary thrombosis; heart failure, angina pectoris, or occlusive arteriosclerosis requiring hospitalization; revascularization (e.g., coronary artery, aorta, peripheral artery)]

### 6.3 Handling of timepoints

Results for blood pressure and degree of independence in activities of daily living (mRS) for timepoints between treatment allocation and 10 days after observation completion will be included in the analysis.

When summarizing the data for blood pressure and degree of independence in activities of daily living (mRS) according to observation timepoint, a range of dates is defined for use as each timepoint, regardless of the observation timepoint indicated in the case report form (Table 6-3). The observation start date is designated day 0, and the day before the observation start date is designated day -1. If multiple evaluation values are observed for a given timepoint, the value obtained closest to the specified date will be used. If the values are equally divergent from the specified date, the latest value will be used.

Table 6-3: Ranges of examination timepoints used in statistical analysis

| Timepoint | Specified Day*1 | Range Used (day) | |
|---|---|---|---|
| Day before observation start | -1 | *3 up to -1*2 | |
| 1 month | 30 | 16 to 44 | (± 2 weeks) |
| 3 months | 91 | 63 to 119 | (± 4 weeks) |
| 6 months | 183 | 127 to 239 | (± 8 weeks) |
| 12 months | 365 | 309 to 421 | (± 8 weeks) |
| 18 months | 548 | 492 to 604 | (± 8 weeks) |
| 24 months | 731 | 675 to 787 | (± 8 weeks) |
| 30 months | 913 | 857 to 969 | (± 8 weeks) |
| 36 months | 1,096 | 1,040 to 1,152 | (± 8 weeks) |
| 42 months | 1,278 | 1,222 to 1,334 | (± 8 weeks) |
| 48 months | 1,461 | 1,405 to 1,517 | (± 8 weeks) |

*1: Specified day calculated using 1 month = 365.25/12 = 30.4375

*2: Including 0 if preliminary examination performed

*3: Handling of exceedingly old data will be examined before analysis.

### 6.4 Handling of missing data

Missing data will not be imputed.

### 6.5 Significance level and confidence coefficient

Because the evaluation of superiority with respect to the primary endpoint will be based on repeated tests, including the interim analysis, an increase in type I errors resulting from multiplicity will occur. Therefore, significance levels (two sided) will be selected using the Haybittle-Peto ad-hoc method.[1, 2]

Interim analysis significance level: 0.0027 (corresponds to z = 3)

Significance level for final analysis: 0.05

The significance level of the tests used in the other efficacy and safety evaluations will be a two-sided level of 5%. A two-sided significance level of roughly 5% will also be used for other evaluations, although an explicit significance level will not be established.

The confidence coefficient used for the estimates will be 95% (two sided).

### 6.6 Multiple comparisons/multiplicity

A significance level (two sided) determined using the Haybittle-Peto ad-hoc method will be used to examine superiority with respect to the primary endpoint.
No multiplicity adjustment will be performed for the efficacy endpoints other than the primary endpoint or for tests and estimates of the safety endpoints.

### 6.7 Adjustment using covariates

If a significant difference is seen for the demographic and other baseline characteristics and the difference is determined to be medically significant, an analysis of the primary endpoint will be performed using the Cox proportional hazard model with this factor as a covariate, and an adjusted hazard ratio will be calculated.

### 6.8 Subgroup analysis

A subgroup analysis will be performed for the primary endpoint, using the variables shown in Table 6.8 as stratification variables. A Cox proportional hazard model for the primary endpoint that includes a comparison group and predictive factors will be examined. The hazard ratio for the combination therapy group versus the monotherapy group will be calculated for each stratification category based on the predictive factors. Uniformity will be examined for the stratification categories.

Table 6.8: Stratification Variables

| Item | Category |
|---|---|
| Sex | Male, Female |
| Age (yr) | up to 49, 50 to 59, 60 to 69, 70 and older |
| | up to 64, 65 and older |
| BMI | < 25, ≥ 25 |
| Past history and concomitant disease | |
| Coronary artery disease | No, Yes |
| Peripheral artery disease | No, Yes |
| Hypertension | No, Yes |

| | |
|---|---|
| Diabetes mellitus | No, Yes |
| Dyslipidemia | No, Yes |
| Chronic renal disease | No, Yes |
| History of symptomatic ischemic stroke | No, Yes |
| Habitual smoking | No, Yes |
| Antiplatelet drugs[*1] | aspirin, clopidogrel |
| Ischemic stroke clinical subtype | lacunar, atherothrombotic |
| At least 50% stenosis of an extracranial artery | No, Yes |
| Degree of independence in activities of daily living (mRS) | 0, 1, 2, 3, 4, 5, 6 |

* 1: At start of observations

### 6.9 Handling of patients with study drug administration errors

For all of the analyses, the data for these patients will be analyzed as data for the groups allocated to a treatment.

## 7. DISPOSITION OF PATIENTS

The follow data summaries will be prepared for all subjects allocated to a treatment (ITT).

For the subjects for whom observations are discontinued or completed, the numbers of subjects observed for 1 year and 2 years and frequency distributions of the reasons for discontinuation/completion will be determined for each treatment group.

Discontinuation of observations: When the following criteria for discontinuation/completion are met and observations are discontinued.

Discontinuation/completion criteria

1) The subject requests that their study participation be discontinued or withdraws their consent

2) A serious adverse events occurs, making continued study participation difficult
3) An evaluated event occurs
4) The study drug is continuously withdrawn for more than 4 weeks
5) The investigator/subinvestigator otherwise determines that the subject's continued study participation would be difficult

# 8. DESCRIPTION OF ANALYSIS SETS

## 8.1 Demographic and other baseline characteristics

Descriptive statistics (no. of subjects, mean, standard deviation, minimum, median, maximum) will be calculated by treatment group for the following variables, and the differences between the groups will be examined using the Wilcoxon test: age, height, weight, BMI, duration of morbidity, blood pressure (systolic and diastolic) on first day of observations, and laboratory values (WBC, RBC, hemoglobin, hematocrit, platelet count, AST, ALT, TC, TG, HDL-C, creatinine, fasting blood glucose, HbA1c).
Frequency distributions for each group will be determined for the variables shown in Table 8.1, and the differences between the groups will be examined using the chi-square or Wilcoxon test.

Table 8.1. Demographic and other baseline characteristics

| Variable | Category |
|---|---|
| Sex | Male, Female |
| Age | up to 49, 50 to 59, 60 to 69, 70 and older |
| | up to 64, 65 and older |
| Duration of morbidity (days) | up to 28, 29 to 56, 57 to 112, 113 and longer |
| BMI | $< 25$, $\geq 25$ |
| Past history and concomitant diseases | |
| Coronary artery disease | No, Yes |
| Peripheral artery disease | No, Yes |
| hypertension | No, Yes |
| Diabetes mellitus | No, Yes |
| Dyslipidemia | No, Yes |

| | |
|---|---|
| Chronic renal disease | No, Yes |
| History of symptomatic ischemic stroke | No, Yes |
| Habitual smoking | No, Yes |
| Antiplatelet drug | aspirin, clopidogrel |
| Clinical subtype | lacunar, atherothrombotic, other, unknown |
| Responsible lesion | supratentorial, infratentorial, both, unknown |
| Microbleeds | No, Yes |
| At least 50% stenosis of a major intracranial artery | No, Yes |
| At least 50% stenosis of an extracranial artery | No, Yes |
| Chest x-ray | Normal, abnormal |
| ECG | Normal, abnormal |
| Degree of independence in activities of daily living (mRS) | 0, 1, 2, 3, 4, 5, 6 |

## 8.2 Compliance

### 8.2.1 Study drug compliance

A frequency distribution will be determined for study drug [cilostazol and the other antiplatelet drugs (aspirin, clopidogrel)] compliance (compliance ≥ 70%, <70%, and whether withdrawn) at each timepoint by treatment group.

### 8.2.2 Observation period

1) The observation period (person-years) will be calculated for each treatment group.
   The observation period will be the number of days from the first day of observations to the last day (observation end date - observation start date).
2) For each group, the observation periods will be classified as being up to 12 months (-365 days), 13 to 24 months (366 to 731 days), 25 to 36 months (732 to 1,096 days), and ≥ 37 months (1,097 days or longer), the frequency distribution will be determined, descriptive statistics (no. of subjects, mean, standard deviation, minimum, median, maximum) calculated, and the groups compared using the Wilcoxon test.

#### 8.2.3 Compliance for drugs other than study drugs

Whether drugs were taken for hypertension (beta-blockers, alpha-beta blockers, calcium channel blockers, angiotensin converting enzyme inhibitors, angiotensin II receptor antagonists, diuretics, and other antihypertensive drugs), diabetes (insulin/insulin analogs, non-insulin oral diabetes drugs), or hyperlipidemia (statins, non-statin lipid-lowering drugs) will be summarized for each timepoint and treatment group.

If a drug is used even once after the start of the observations, it will be treated as having been used throughout the study period.

# 9. EFFICACY EVALUATION

## 9.1 Primary endpoint

The first recurrence of ischemic stroke between the first and last observation days (during the observation period) will be included in the analysis.

Subjects discontinued for reasons other than occurrence of the primary endpoint and subjects who continue to study completion will be treated as censored subjects. The censor date will be considered the observation completion date.

1) A between-groups comparison for the period from the observation start date to the date of ischemic stroke recurrence will be performed using the log-rank test, and the superiority of the combination therapy group versus the monotherapy group will be examined.
2) Survival coefficients for the period from the observation start date to the date of ischemic stroke recurrence will be estimated for each treatment group using the Kaplan-Meier method and plotted.
3) The hazard ratio for ischemic stroke recurrence in the combination therapy group versus the monotherapy group and its 95% confidence interval will be determined using a Cox proportional hazard model.
4) An adjusted hazard ratio will be calculated using a Cox proportional hazard model that includes as covariates age (each 1 year), sex, ischemic stroke clinical subtype (atheromic, lacunar, other, unknown), mRS (ordered categorical data).
5) The annual incidence in each group will be calculated using the person-year and Kaplan-Meier methods. The 95% confidence interval of the incidence will be calculated using the approximate Poisson method and the Greenwood method. One year will be considered 365.25 days for the calculations.

6) A subgroup analysis will be performed for each variable shown in Table 6.8 using a Cox proportional hazard model that includes a comparison group and predictive factors. The hazard ratio for the combination therapy group versus the monotherapy group will be calculated for each stratification category based on the predictive factors. Uniformity will be examined for the stratification categories based on the interaction between the comparison group and predictive factors.
7) Frequency distributions for each treatment group will be determined for the findings regarding the clinical subtype of ischemic stroke (lacunar, atherothrombotic, other, unknown) and responsible lesion (supratentorial, infratentorial, both, unknown) at the time of ischemic stroke onset.

## 9.2 Secondary endpoints

The secondary endpoints will be those indicated below. Events or accidents that occur between the observation start date and the observation end date (during the observation period) will be included in the analysis.

1. Stroke (ischemic stroke, intracerebral hemorrhage, subarachnoid hemorrhage)
2. Intracerebral hemorrhage or subarachnoid hemorrhage
3. Ischemic cerebrovascular disorder (ischemic stroke, TIA)
4. Death from any cause
5. Stroke (ischemic stroke, intracerebral hemorrhage, subarachnoid hemorrhage), myocardial infarction, or vascular death
6. All vascular events: stroke, myocardial infarction, and other vascular events [e.g., aortic dissection or rupture; pulmonary embolism; heart failure, angina pectoris, or occlusive arteriosclerosis requiring hospitalization; and revascularization (e.g., coronary artery, aorta, peripheral artery)]

### 9.2.1 Stroke (ischemic stroke, intracerebral hemorrhage, subarachnoid hemorrhage)

The first event involving ischemic stroke, intracerebral hemorrhage, or subarachnoid hemorrhage will be included in the analysis.

Subjects discontinued for reasons other than occurrence of an event included in the analysis and subjects who continue to study completion will be considered censored subjects. The censor date will be considered the observation completion date.

1) A between-groups comparison for the period from the observation start date to the date of ischemic stroke onset will be performed using the log-rank test, and the superiority of the combination therapy group versus the monotherapy group will be examined.
2) Survival coefficients for the period from the observation start date to the date of the event will be estimated for each treatment group using the Kaplan-Meier method and plotted.
3) The hazard ratio for the occurrence of an event in either of the 2 groups and its 95% confidence interval will be determined using the Cox proportional hazard model.

The same analysis described in section 9.2.1 will be performed for the events indicated in sections 9.2.2 to 9.2.6.

### 9.2.2 Intracerebral hemorrhage or subarachnoid hemorrhage

The first event involving intracerebral hemorrhage or subarachnoid hemorrhage will be included in the analysis.
Subjects discontinued for reasons other than occurrence of an event included in the analysis and subjects who continue to study completion will be considered censored subjects. The censor date will be considered the observation completion date.

### 9.2.3 Ischemic cerebrovascular disorder (ischemic stroke, TIA)

The first event involving ischemic stroke or TIA will be included in the analysis.
Subjects discontinued for reasons other than occurrence of an event included in the analysis and subjects who continue to study completion will be considered censored subjects. The censor date will be considered the observation completion date.

### 9.2.4 Death from any cause

All deaths will be included in the analysis.
Subjects discontinued for reasons other than death, which will be included in the analysis, and subjects who continue to study completion will be considered censored subjects. The censor date will be considered the observation completion date.

### 9.2.5 Stroke (ischemic stroke, intracerebral hemorrhage, subarachnoid hemorrhage), myocardial infarction, or vascular death

The first event involving ischemic stroke, intracerebral hemorrhage, subarachnoid hemorrhage,

myocardial infarction, or vascular death will be included in the analysis.
Subjects discontinued for reasons other than occurrence of an event included in the analysis and subjects who continue to study completion will be considered censored subjects. The censor date will be considered the observation completion date.

#### 9.2.6 All vascular events: stroke, myocardial infarction, and other vascular events [e.g., aortic dissection or rupture; pulmonary embolism; heart failure, angina pectoris, or occlusive arteriosclerosis requiring hospitalization; and revascularization (e.g., coronary artery, aorta, peripheral artery)]

The first event involving stroke, myocardial infarction, or another vascular event [e.g., aortic dissection or rupture; pulmonary embolism; heart failure, angina pectoris, or occlusive arteriosclerosis requiring hospitalization; and revascularization (e.g., coronary artery, aorta, peripheral artery)] will be included in the analysis.
Subjects discontinued for reasons other than occurrence of an event included in the analysis and subjects who continue to study completion will be considered censored subjects. The censor date will be considered the observation completion date.

## 10. SAFETY EVALUATION

### 10.1 Adverse events

Events that occur between the observation start date and the observation end date (during the observation period) will be included in the analysis. Adverse event summaries according to system organ class and type of event will be prepared using MedDRA/J.

1) The number of patients with adverse events and their incidence (100 x no. of patients with adverse events / no. of ITT patients) will be determined according to treatment group. The incidence of adverse events will be compared between groups using the chi-square test.
2) Incidence will be determined according to system organ class and event type (preferred term) based on terminology conversions using MedDRA/J, and the chi-square test will be used to compare these incidences across groups.
3) A frequency distribution for each level of severity will be determined based on the converted terms. If the same type of event occurs more than once in the same subject, the severest of these events will be used.

### 10.2 Adverse drug reactions

Adverse events for which a causal relationship to the study drug cannot be ruled out will be considered adverse drug reactions and analyzed in the same manner as indicated in subsections 1), 2), and 3) of section 10.1 (adverse events).

## 10.3 Serious adverse events

Adverse events rated as "serious" in the assessment of seriousness will be considered serious adverse events and analyzed in the same manner as indicated in subsections 1) and 2) of section 10.1 (adverse events).

## 10.4 Serious adverse drug reactions

Adverse drug reactions rated as "serious" in the assessment of seriousness will be considered serious adverse drug reactions and analyzed in the same manner as indicated in subsections 1) and 2) of section 10.1 (adverse events).

## 10.5 Hemorrhagic adverse events

Hemorrhagic adverse events will be analyzed in the same manner as indicated in subsections 1) and 2) of section 10.1 (adverse events). Incidence will be determined for all hemorrhagic events and for hemorrhage determined to be severe or life-threatening based on the GUSTO bleeding criteria. Incidence will be determined according to system organ class and event type (preferred term) based on terminology conversions using MedDRA/J, and the chi-square test will be used to compare these incidences across groups.

GUSTO bleeding criteria

-Severe or life-threatening hemorrhage: Intracranial bleeding or bleeding that causes substantial hemodynamic compromise requiring treatment

-Moderate bleeding: Bleeding which requires blood transfusion

-Minor bleeding: Other bleeding, neither requiring transfusion nor causing hemodynamic compromise

## 10.6 Blood pressure

Descriptive statistics will be calculated for the blood pressure (systolic, diastolic) values for each treatment group and timepoint and for the difference from the observation start date.

A figure will be prepared showing the change in the mean and standard deviation over time for each treatment group and value at each timepoint.

### 10.7 Other safety endpoints

**Degree of independence in activities of daily living (mRS)**

Evaluate as one of following 7 levels according to Japanese Modified Rankin Scale (Japanese Guidelines for Management of Stroke 2009). A frequency distribution will be determined for each treatment group and timepoint, and a shift table will be prepared for the values at each timepoint before and after the start of the observation for each treatment group.

0. No symptoms at all
1. No significant disability despite symptoms: able to carry out all usual duties and activities
2. Slight disability: unable to carry out all previous activities, but able to look after own affairs
without assistance
3. Moderate disability: requiring some help, but able to walk without assistance
4. Moderate to severe disability: unable to walk without assistance and unable to attend to own bodily needs without assistance
5. Severe disability: bedridden, incontinent and requiring constant nursing care and attention
6. Dead

## 11. REFERENCES

1) Haybittle JL. Repeated assessment of results in clinical trials of cancer treatment. *Br J Radiol* 1971; 44: 793-7.

2) Peto R, Pike MC, Armitage P, et al. Design and analysis of randomized clinical trials requiring prolonged observation of each patient. I. Introduction and design. *Br J Cancer* 1976; 34: 585-612.

3) Lakatos E, Lan KK. A comparison of sample size methods for the logrank statistic. Stat Med 1992; 11: 179-91.

End

## 12. REVISION HISTORY

| Version No. | Date | Prepared by | Revision |
|---|---|---|---|
| 0.10 | January 24, 2014 | EPS Kazumoto Hirabayashi | First version prepared |
| 0.20 | February 10, 2014 | EPS Kazumoto Hirabayashi | Revised according to findings of January 30 meeting |
| 0.30 | February 18, 2014 | EPS Kazumoto Hirabayashi | Revised according to February 13 findings |
| 1.00 | March 10, 2014 | EPS Kazumoto Hirabayashi | First version finalized |
